# A Multi‐Property Optimizing Generative Adversarial Network for de novo Antimicrobial Peptide Design

**DOI:** 10.1002/advs.202503443

**Published:** 2025-08-11

**Authors:** Jiaming Liu, Tao Cui, Tao Wang, Lishan Lin, Xi Zeng, Dazhi Lu, Shaoqing Jiao, Jun Wang, Xiaoyan Li, Shuyuan Xiao, Dongna Xie, Xuecheng Wang, Yongtian Wang, Xuequn Shang, Yinbo Niu, Zhongyu Wei, Jiajie Peng

**Affiliations:** ^1^ AI for Science Interdisciplinary Research Center, School of Computer Science Northwestern Polytechnical University NO. 1 Dongxiang Road Xi'an 710129 China; ^2^ Key Laboratory of Big Data Storage and Management, Northwestern Polytechnical University Ministry of Industry and Information Technology NO. 1 Dongxiang Road Xi'an 710129 China; ^3^ School of Life Sciences Northwestern Polytechnical University NO. 1 Dongxiang Road Xi'an 710129 China; ^4^ School of Software Northwestern Polytechnical University NO. 1 Dongxiang Road Xi'an 710129 China; ^5^ School of Data Science Fudan University NO. 220 Handan Road Shanghai 200433 China

**Keywords:** antimicrobial peptides, de novo design, generative adversarial network, multi‐property optimizing

## Abstract

Antimicrobial peptides (AMPs) play a crucial role in developing novel anti‐infective drugs due to their broad‐spectrum antimicrobial activity and lower likelihood of causing bacterial resistance. However, laboratory synthesis of AMPs is tedious and time‐consuming. Existing computational methods have limited capability in optimizing multiple desired properties simultaneously. Here, a Multi‐Property Optimizing Generative Adversarial Network (MPOGAN) is proposed to iteratively learn the relationship between peptides and multi‐properties with a dynamically updated dataset. With the increase of the dataset quality, the ability of the model to design AMPs with multiple desired properties is improved. Through extensive computational tests, MPOGAN exhibits superior performance in generating AMPs with multiple desired properties, including potent antimicrobial activity, reduced cytotoxicity, and increased diversity. Ten designed AMPs are chemically synthesized, nine of which exhibited antimicrobial activity and low cytotoxicity. Notably, two of these peptides showed potent broad‐spectrum antimicrobial activity coupled with reduced cytotoxicity, highlighting their potential for downstream applications.

## Graphical abstract

A Multi‐Property Optimizing Generative Adversarial Network (MPOGAN) is proposed to iteratively learn the relationship between peptides and multiple properties using a dynamically dataset. As the quality of the dataset improves, MPOGAN's ability to design antimicrobial peptides (AMPs) with multiple desired properties is enhanced. MPOGAN demonstrates superior performance in generating AMPs with potent antimicrobial activity, reduced cytotoxicity, and increased diversity.

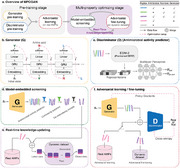

## 1 Introduction

As underlined by the World Health Organization's (WHO) caution, we are rapidly approaching a time when antibiotics may cease to be effective in treating infections.^[^ ^1^ ^]^ Antimicrobial‐resistant pathogens caused more than 2.8 million infections and over 35 000 deaths annually in the United States from 2012 to 2017.^[^ ^2^ ^]^ Increasing drug resistance of common pathogens urgently needs the discovery of new effective molecules.^[^ ^3^ ^]^ Antimicrobial peptides (AMPs), small amphipathic peptides with a net positive charge under physiological pH,^[^ ^4^ ^]^ are often hailed as “natural antibiotics” due to their broad‐spectrum antimicrobial activity, minimal toxicity, and lower likelihood of triggering bacterial resistance.^[^ ^5^, ^6^, ^7^, ^8^ ^]^ Therefore, AMPs emerge as potential game‐changers in addressing this problem.^[^ ^9^ ^]^

Discovery of AMPs in the biological laboratory is typically time‐consuming and costly, which has driven the study of efficient computational approaches for AMP design.^[^ ^10^, ^11^, ^12^ ^]^ One type of widely studied method is predictive models, which take peptides as input and predict specific properties (Antimicrobial activity,^[^ ^13^, ^14^, ^15^, ^16^, ^17^, ^18^ ^]^ toxicity,^[^ ^19^ ^]^ secondary structure,^[^ ^20^ ^]^ etc.) of these peptides. Employing predictive models trained on natural AMPs, some studies develop the quantitative structure‐activity relationship (QSAR) models to identify potential AMPs in a peptide database that have not been experimentally validated.^[^ ^4^, ^21^, ^22^ ^]^ These predictive models can identify existing peptides with desired properties, but cannot design novel AMPs. Another type of computational method is evolutionary algorithms,^[^ ^23^, ^24^, ^25^ ^]^ which guide peptide sequence evolution by randomly mutating existing AMPs and viability assessment. However, these methods are limited by the local search space, making it difficult to discover potential AMPs that are significantly different from known AMPs but are more effective. Recently, deep generative methods in machine learning, including Autoregressive Models (AMs),^[^ ^26^, ^27^, ^28^, ^29^ ^]^ Variational Autoencoders (VAEs),^[^ ^10^, ^11^, ^30^, ^31^, ^32^ ^]^ and Generative Adversarial Networks (GANs),^[^ ^7^, ^33^, ^34^, ^35^, ^36^, ^37^, ^38^, ^39^ ^]^ have emerged as efficient computational approaches to generate AMPs de novo.

The clinical application of AMPs requires potent antimicrobial activity and limited side effects. One prominent side effect is the toxicity against eukaryotic cells, which is a major obstacle to AMPs becoming therapeutic agents.^[^ ^6^, ^19^, ^39^, ^40^ ^]^ Many existing methods primarily focus on optimizing antimicrobial activity, which limits their ability to generate AMPs with multiple desired properties. Although some approaches^[^ ^4^, ^10^, ^11^, ^41^ ^]^ have incorporated in silico step‐by‐step screening to identify AMP candidates with these properties, the success rate of AMP wet‐laboratory verification remains constrained. Recent studies have explored innovative multi‐objective optimization approaches,^[^ ^25^, ^42^, ^43^ ^]^ while these methods face challenges in scalability to diverse optimization requirements. On the other hand, while properties such as antimicrobial activity and cytotoxicity are influenced by key physicochemical features like net charge and amphiphilicity,^[^ ^10^, ^44^ ^]^ optimizing multiple properties simultaneously through adjustments to these features remains challenging due to the inherent trade‐offs between them. For example, increasing net charge or hydrophobicity can enhance antimicrobial activity but also increase the risk of cytotoxicity to eukaryotic cells.^[^ ^44^, ^45^, ^46^ ^]^ In this context, a straightforward idea is to train a model using AMPs with multiple desired properties. Unfortunately, for most experimentally validated AMPs, only the antimicrobial activity validation is performed, resulting in the lack of training data for multi‐property optimization.^[^ ^47^ ^]^

This study introduces a Multi‐Property Optimizing Generative Adversarial Network (MPOGAN). We propose an extended feedback‐loop GAN framework, specifically designed to tackle the scarcity of high‐quality AMPs with multiple desired properties as training data. To fill this gap, multiple plug‐and‐play model‐embedded evaluators are designed to screen for high‐quality training data from generated peptides. Furthermore, a Real‐Time Knowledge‐Updating (RTKU) strategy is proposed to iteratively update the training dataset dynamically. By learning from iteratively updated training datasets to model the relationship between multiple properties and peptides, MPOGAN can design novel AMPs with potent antimicrobial activity, reduced cytotoxicity, and diversity. The extensive evaluation of AMP candidates indicates that MPOGAN outperforms other state‐of‐the‐art methods.^[^ ^10^, ^25^, ^26^, ^28^, ^30^, ^32^, ^33^, ^35^, ^36^ ^]^ Ten predicted candidates were chemically synthesized. Among these, nine demonstrated antimicrobial activity and low cytotoxicity. Notably, two peptides exhibited potent broad‐spectrum antimicrobial activity with even lower cytotoxicity against MC3T3‐E1 cells. Further studies for the most promising AMP (MPOP‐03) demonstrated strong safety and selectivity profiles, along with potent activity against drug‐resistant bacterial strains. In summary, MPOGAN offers a powerful computational approach for de novo design of AMPs with multiple desired properties, thereby advancing AI‐aided drug discovery.

## 2 Results

### 2.1 MPOGAN: A Deep Generative Framework for de Novo AMP Design

**Figure 1:**
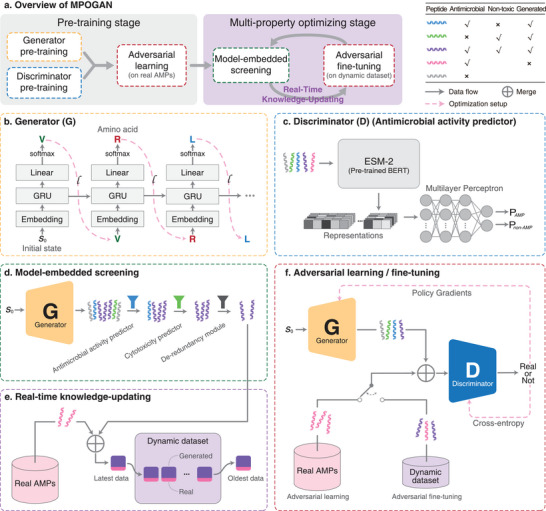
The workflow of MPOGAN. a) Overview of MPOGAN. The pre‐training stage (left) contains generator pre‐training (yellow), discriminator pre‐training (blue), and adversarial learning on experimentally validated AMPs (red); the multi‐property optimizing (MPO) stage (right) consists of multiple iterations of model‐embedded screening (green), real‐time knowledge‐updating (purple), and adversarial fine‐tuning on dynamic dataset (red). The color of the dashed box corresponds to b–f. b) The generator is based on a Recurrent Neural Network (RNN) consisting of three layers. c) The discriminator consists of a pre‐trained Bidirectional Encoder Representations from Transformers (BERT) (ESM‐2) with fixed parameters and a Multilayer Perceptron (MLP) with trainable parameters. The antimicrobial activity predictor shares the same architecture with the discriminator. d) A model‐embedded screening process comprises three plug‐and‐play evaluators (funnels), where the parameters of all evaluators are fixed. e) A real‐time knowledge‐updating strategy updates the dynamic dataset via first‐in‐first‐out, where the dynamic dataset has a fixed size. f) Adversarial learning on experimentally validated AMPs during the pre‐training stage, and adversarial fine‐tuning on the dynamic dataset during the MPO stage.

To de novo design AMPs with multiple desired properties, specifically potent antimicrobial activity, reduced cytotoxicity, and diversity, we propose the MPOGAN framework (**Figure** **1**), which consists of two main stages: a pre‐training stage (Figure 1a left) and a multi‐property optimizing (MPO) stage (Figure 1a right). In the pre‐training stage, MPOGAN undergoes generator pre‐training, discriminator pre‐training, and adversarial learning using experimentally validated AMPs and non‐AMPs. This process enables MPOGAN to learn the characteristics of experimentally validated AMPs and generate analogous peptides. Inspired by the previous study,^[^ ^48^ ^]^ MPOGAN consists of a generator (Figure 1b) based on a recurrent neural network (RNN) and a discriminator (Figure 1c) based on a protein large language model (ProLLM). The generator produces amino acid sequences from an initial input token. By modeling the generator with an RNN, MPOGAN can learn the semantic relationships between discrete amino acids. The discriminator consists of a pre‐trained ProLLM, ESM‐2,^[^ ^49^ ^]^ for feature encoding, and a Multilayer Perceptron (MLP) as prediction head for binary classification. The discriminator takes amino acid sequences as input and outputs the probabilities of being AMPs. ProLLM for feature encoding enables MPOGAN to leverage the inherent biological information between amino acids.^[^ ^39^ ^]^

In the MPO stage, we enhance MPOGAN with the ability to optimize multiple properties through a model‐embedded screening process (Figure 1d), a Real‐Time Knowledge‐Updating (RTKU) strategy (Figure 1e), and adversarial fine‐tuning (Figure 1f). First, to screen high‐quality candidates from MPOGAN‐generated peptides, the model‐embedded screening includes three plug‐and‐play evaluators: a ProLLM‐based antimicrobial activity predictor (LLM‐AAP) trained on the experimentally validated AMP dataset to identify highly potent AMP candidates, a cytotoxicity predictor^[^ ^19^ ^]^ to select candidates with reduced cytotoxicity, and a de‐redundancy module^[^ ^50^ ^]^ to ensure diversity among the candidates. Then, following the feedback‐loop architecture,^[^ ^36^ ^]^ we propose the RTKU strategy to guide the updating of a dynamic dataset. The data used for updating consists of two parts: a large portion of high‐quality candidates from the model‐embedded screening, which guides MPOGAN to learn high‐quality features, and a small portion from the experimentally validated AMP dataset, which ensures the generalization capability of MPOGAN. The RTKU strategy ensures MPOGAN captures new features without ignoring the existing ones. Finally, adversarial fine‐tuning is performed to optimize MPOGAN using the dynamic dataset, thereby fully leveraging the knowledge acquired by model‐embedded screening. In summary, we conceptualize the MPOGAN as a framework for modeling the properties‐peptides relationship: Initially, the generator is limited to creating analogs of existing AMPs. As a diverse range of peptide sequences with desired properties is continuously added to the dynamic dataset as positive samples, the discriminator is encouraged to shift its paradigm from merely “distinguishing whether a peptide is a experimentally validated AMP” to “determining whether a peptide is a multi‐property optimized AMP”. This ongoing interaction drives the generator to produce peptides that are increasingly difficult to classify—specifically, peptides optimized for multiple properties. Ultimately, the final generator is capable of generating peptides that exhibit desired properties. Further details about the MPOGAN framework can be found in the “Methods” section.

### 2.2 MPOGAN Outperforms the Baseline Methods in Generating AMPs de Novo

**Figure 2:**
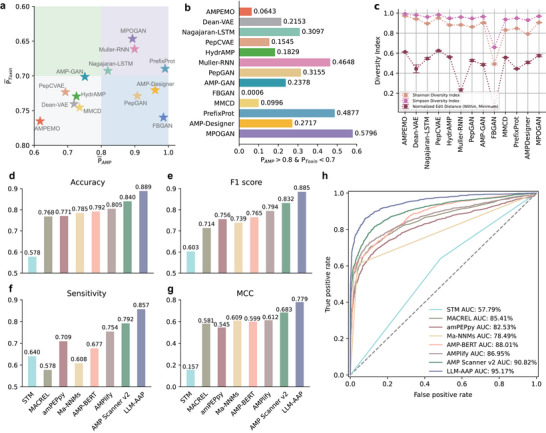
Comparison assessments of MPOGAN and generative baseline methods (a‐c), LLM‐AAP and predictive baseline methods (d‐h). Evaluations were performed on AMP candidates generated by the baseline models and MPOGAN. The probabilities of antimicrobial activity (*P*_AMP_) and cytotoxicity (*P*_AMP_) are predicted using predictive models from multiple previously published studies (see Note S2.2, Supporting Information). a) Comparison of the mean antimicrobial activity probabilities (x‐axis) and the mean cytotoxicity probabilities (y‐axis) of the candidates. Pentagrams of various colors are used to distinguish the models. Models located in areas with different background colors indicate varying generation preferences: gray (bottom‐left area) represents low activity and high toxicity; blue (bottom‐right area) represents high activity and high toxicity; green (top‐left area) represents low activity and low toxicity; purple (top‐right area) represents high activity and low toxicity. b) Comparison of the proportion (x‐axis) of the candidates (y‐axis) that meet the combined thresholds (*P*_AMP_ > 0.8 and *P*_Toxin_ < 0.7). The numbers on the right indicate the proportions of sequences. c) Comparison of the diversity of AMP candidates generated by MPOGAN and generative baseline methods (n=3 independent samples with three fixed random seeds). The evaluation metrics include the normalized Shannon diversity index, Simpson diversity index, and minimum within‐group edit distances. d–g) Predictive performance of LLM‐AAP, in comparison to STM, MACREL, amPEPpy, Ma‐NNMs, AMP‐BERT, AMPlify, and AMPScanner v2 on the independent test set: d) accuracy, e) F1 score, f) sensitivity, and g) Matthews correlation coefficient (MCC). h) Receiver‐operating characteristic (ROC) curves of LLM‐AAP and predictive baseline models. The diagonal dashed line indicates random prediction. AUC values for the models are labeled in the bottom right.

We compared the performance of MPOGAN with nine state‐of‐the‐art deep generative methods in generating high‐quality AMPs: AMPEMO,^[^ ^25^ ^]^ Dean‐VAE,^[^ ^32^ ^]^ Nagajaran‐LSTM,^[^ ^26^ ^]^ PepCVAE,^[^ ^30^ ^]^ HydrAMP,^[^ ^10^ ^]^ Muller‐RNN,^[^ ^28^ ^]^ PepGAN,^[^ ^35^ ^]^ AMP‐GAN,^[^ ^33^ ^]^ FBGAN,^[^ ^36^ ^]^ AMP‐Designer,^[^ ^51^ ^]^ MMCD,^[^ ^52^ ^]^ and PrefixProt.^[^ ^53^ ^]^ Following the evaluation protocols in the previously published study,^[^ ^10^ ^]^ we evaluated three key optimized properties of generated AMP candidates using several predictors,^[^ ^17^, ^18^, ^19^ ^]^ including antimicrobial activity, cytotoxicity, and diversity (**Figure** **2**a–c; Figures 7 and 8, Supporting Information). The details of comparison models and evaluation settings can be found in Note S2 (Supporting Information).

For the balance between antimicrobial activity and cytotoxicity, MPOGAN achieves the most effective trade‐off relative to other baseline methods (Figure 2a). The proportion of candidates meeting a combined threshold (*P*_AMP_ > 0.8 and *P*_Toxin_ < 0.7) (Figure 2b) reveals that MPOGAN achieves a high success rate, which is 9.19% higher than the second‐best method, PrefixProt. Considering the total number of sequences generated, MPOGAN generates 59 times more candidates that satisfy the combined threshold compared to Muller‐RNN. In contrast, FBGAN generates only a minimal proportion (0.06%) of candidates meeting the threshold, emphasizing the limitations of optimizing for a single property in isolation. The distribution of antimicrobial activity and cytotoxicity probabilities of candidates generated by each method (Figure S8, Supporting Information) show that MPOGAN generates a significantly larger number of candidates satisfying the combined threshold, with most AMPs concentrated in the high‐activity, low‐toxicity region (top‐right). In contrast, for most compared methods, the generated AMPs concentrated in the high‐activity, high‐toxicity region (bottom‐right). These results indicate that through synergistic optimization of antimicrobial activity and toxicity, MPOGAN can generate candidates with better multiple properties. To evaluate diversity, we calculated the normalized Shannon diversity index, Simpson diversity index, and minimum within‐group edit distances (see Note S2.2, Supporting Information) for the sequence groups generated by each method. The diversity metrics of MPOGAN outperform most baseline methods (Simpson diversity index of 0.91, Shannon diversity index of 0.97, minimum within‐group edit distance of 0.58) (Figure 2c). In contrast, although antimicrobial activity and toxicity are balanced, the sequence similarity generated by Muller‐RNN is relatively high.

### 2.3 Performance Evaluation of Antimicrobial Activity Predictor

Antimicrobial activity prediction can be regarded as an AMP identification task. We compared the performance of our ProLLM‐based Antimicrobial Activity Predictor (LLM‐AAP) with six state‐of‐the‐art AMP identification methods: STM,^[^ ^15^ ^]^ MACREL,^[^ ^13^ ^]^ amPEPpy,^[^ ^14^, ^54^ ^]^ Ma‐NNMs,^[^ ^21^ ^]^ AMP‐BERT,^[^ ^16^ ^]^ AMPlify,^[^ ^18^ ^]^ and AMP Scanner v2.^[^ ^17^ ^]^ We applied accuracy, sensitivity, F1 score, Matthews Correlation Coefficient (MCC), and Area Under the Curve (AUC) as the model evaluation metrics. Details of these methods and evaluation settings can be found in Note S3 (Supporting Information).

The experiment results show that LLM‐AAP achieves the highest performance among all methods in five evaluation metrics (Figure 2d–h; Table S1 and Figure S11, Supporting Information). Compared to other methods, the accuracy of our predictor is 4.87% higher than the runner‐up methods (from 0.8400 to 0.8887) (Figure **2**d), the F1 score is 5.31% higher (from 0.8319 to 0.8850) (Figure 2e), the sensitivity is 6.49% higher (from 0.7917 to 0.8566) (Figure 2f), the MCC is 9.57% higher (from 0.6832 to 0.7790) (Figure 2g), and the AUC is 4.35% higher (from 90.82% to 95.17%) (Figure 2h). The improvement in results is attributed to the fact that LLM‐AAP enables the extraction of complex intrinsic biological information from peptides using ProLLM. In conclusion, LLM‐AAP can achieve significant improvements in identifying AMP candidates compared to state‐of‐the‐art methods.

The RTKU strategy of MPOGAN updates the AMP candidates refined by the model‐embedded screening into the dynamic dataset. Therefore, the quality of these peptides directly affects the optimization direction for MPOGAN. If the model‐embedded screening cannot accurately identify the desired AMP candidates, MPOGAN will be fine‐tuned in an unknown direction. As the first evaluator of the model‐embedded screening process, the superior performance of LLM‐AAP ensures that MPOGAN continuously learns the features of AMPs with potent antimicrobial activity during adversarial fine‐tuning. Additionally, the MPOGAN discriminator has the same network architecture as LLM‐AAP, ensuring that the superior performance of LLM‐AAP translates to a highly effective MPOGAN discriminator with robust discrimination ability.

### 2.4 Features of AMP Candidates Generated by MPOGAN

In this section, we evaluated the distributions of key molecular features implicated in the antimicrobial nature of MPOGAN‐generated AMPs (MPOPs). Here, we compared the key molecular features of three sets, including the experimentally validated AMPs we collected (labeled as experimentally validated AMPs), the 50 000 AMP candidates directly generated by MPOGAN (labeled as MPOPs‐50k), and the 124 stringent AMP candidates (labeled as MPOPs‐124). MPOPs‐124 includes the most promising AMP candidates from MPOPs‐50k (Table S5, Supporting Information). Details on generating MPOPs‐124 can be found in “Methods” and Figure 16 (Supporting Information). The key molecular features analyzed include amino acid composition, charge, isoelectric point (pI), hydrophobic moment (HM), and instability index (II). These molecular features are implicated in the peptide‐membrane interaction and structural stability.^[^ ^11^, ^55^ ^]^

**Figure 3:**
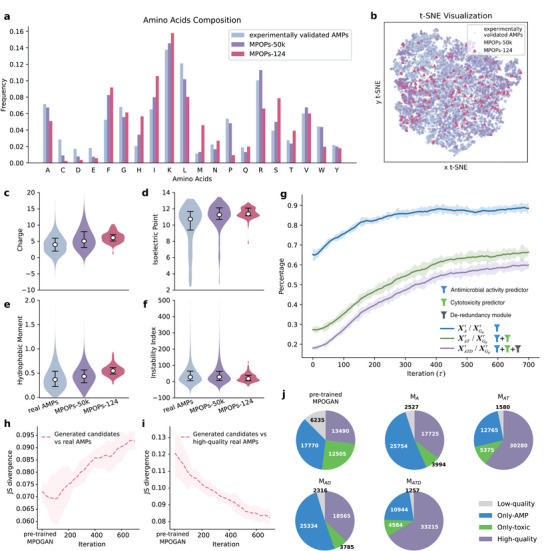
Characteristic analysis of MPOGAN‐generated AMP candidates. a, c–f) Comparison of a amino acid composition, c) charge, d) isoelectric point (pI), e) hydrophobic moment, and f instability index distribution of experimentally validated AMPs with MPOGAN‐generated AMP candidates (MPOPs‐50k: 50 000 peptides that are directly generated by MPOGAN; MPOPs‐124: 124 peptides that are obtained through the preliminary selection pipeline (see “Methods”)). All physicochemical properties are calculated using modlAMP. b) t‐SNE visualization. Nine physicochemical features (charge, isoelectric point, aromaticity, Eisenberg hydrophobicity, hydrophobic moment, hydrophobic ratio, charge density, instability index, and aliphatic index) of peptides are calculated and downscaled to 2 dimensions with a perplexity of 30. g) Percentage variation with τ in the size of AMP candidate sets that pass the model‐embedded evaluators relative to the size of the AMP candidate set generated by the MPOGAN‐generator. The translucent area outside the curve indicates the range of fluctuations in the data. h,i) Variation with τ in Jensen–Shannon (JS) divergence of the distribution of the AMP candidate set generated by the MPOGAN‐generator relative to the distribution of h the experimentally validated AMP dataset, and i) the high‐quality subset in the experimentally validated AMPs dataset. j) Ablation studies for various combinations of model‐embedded evaluators, with each generated set contains 50 000 peptides. The property distribution of AMP candidate sets generated by generators are presented, where the numbers indicate the subset sizes.

Compared to experimentally validated AMPs, both MPOPs‐50k and MPOPs‐124 contain higher proportions of Phenylalanine (F) and Isoleucine (I), which have hydrophobic side chains; higher proportions of Histidine (H) and Lysine (K), which have positively charged side chains; and lower proportions of Aspartic acid (D) and Glutamic acid (E), which have negatively charged side chains (**Figure** **3**a). These results demonstrate that MPOGAN can generate AMP candidates with enhanced lipophilicity and a stronger positive net charge. These two features help peptides better penetrate and disrupt the cell membrane of bacteria.^[^ ^8^ ^]^ The MPOPs‐124 contains a higher proportion of neutral amino acids (Methionine (M), Asparagine (N), Glutamine (Q), Threonine (T), Serine (S)) compared to both the experimentally validated AMPs dataset and MPOPs‐50k, as well as fewer Tryptophan (W), Arginine (R), and Proline (P) (Figure 3a). This indicates that MPOPs‐124 peptides exhibit a more balanced hydrophilic profile, potentially enhancing their solubility and stability in aqueous environments. Additionally, the reduction in W, R, and P might contribute to a lower likelihood of aggregation and structural rigidity, thereby improving their flexibility and ability to adapt to different bacterial membrane environments. The overall amino acid composition of MPOPs‐124 suggests a subtle optimization for antimicrobial activity, combining hydrophilic balance with enhanced peptide‐membrane interaction.

In terms of physicochemical features, MPOPs‐50k maintain features similar to experimentally validated AMPs, while MPOPs‐124 exhibit a more concentrated distribution across various physicochemical properties (Figure 3c–f; Figure S13, Supporting Information). In detail, for some features, including charge (Figure 3c), pI (Figure 3d), and HM (Figure 3e), the mean values of MPOPs‐50k are higher than those of experimentally validated AMPs, and the mean values of MPOPs‐124 are even higher. The enhancement of these properties is beneficial for peptide‐membrane interactions.^[^ ^55^ ^]^ Additionally, peptides with an II < 40 are considered stable.^[^ ^56^ ^]^ The mean value of II for MPOPs‐50k and MPOPs‐124 is lower than that of experimentally validated AMPs. The proportion of peptides with II < 40 in MPOPs‐50k (60.57%) increased by 0.5%, and in MPOPs‐124 (76.61%) increased by 16.54%, compared to experimentally validated AMPs (60.07%) (Figure 3f). This finding indicates that the generated AMP candidates, particularly those in the MPOPs‐124, are more stable than experimentally validated AMPs. We further evaluated the overall distribution of the three datasets across the nine physicochemical properties (Figure 3b). By reducing the dimensionality of the standardized nine physicochemical properties to two dimensions using t‐SNE,^[^ ^57^ ^]^ we observed that the distribution of MPOPs‐50k is similar to that of experimentally validated AMPs, while the distribution of MPOPs‐124 is more concentrated, aligning with previous findings. These observations demonstrate that MPOGAN is effective in generating AMP candidates with physicochemical features similar to those found in natural AMPs. The MPOPs‐124 refined the candidate pool to focus on peptides with more specific and desired features.

To compare the diversity and novelty of MPOGAN‐generated and experimentally validated AMP, the following analysis was carried out. The minimum normalized within‐group edit distance was used to assess sequence diversity, with higher values indicating higher diversity within the group. The diversity of MPOPs‐50k and MPOPs‐124 is significantly higher than that of experimentally validated AMPs, suggesting that MPOGAN can generate more diverse candidates than experimentally validated AMPs (Figure S14a, Supporting Information). Similarly, the minimum normalized inter‐group edit distance was used to evaluate the novelty of MPOGAN‐generated peptides relative to experimentally validated AMPs (Supplementary Figure 14b), with higher values indicating higher novelty. MPOPs‐50k exhibits high novelty, while MPOPs‐124 shows even higher novelty, suggesting that MPOGAN can generate novel candidates. In summary, through iterative learning and continuous refinement of high‐quality sequence features, MPOGAN is able to generate AMP candidates that exhibit better physicochemical properties, greater diversity and novelty compared to experimentally validated AMPs.

We also evaluated the homology of AMP candidates against experimentally validated AMPs (Figure S14c–e and Table S2, Supporting Information). We calculated the percentage of pMPOPs‐50k (50 000 AMP candidates generated by pre‐trained MPOGAN), MPOPs‐50k, and MPOPs‐124 in different categories of Expect value (E‐value).^[^ ^58^ ^]^ The E‐value for the match with the highest score was considered, as obtained by performing a BLAST similarity search^[^ ^59^ ^]^ against experimentally validated AMPs. A larger E‐value implies fewer repeated sequence fragments and a smaller likelihood of homology. Compared with pMPOPs‐50k, MPOPs‐50k exhibits higher homology against experimentally validated AMPs, and MPOPs‐124 exhibits the highest. This finding suggests that the MPO stage slightly increases the correlation between AMP candidates and experimentally validated AMPs. Meanwhile, the AMP candidate sequences generated by MPOGAN still maintain diversity (Figure S14b, Supporting Information), and the majority of E‐values are still higher than 0.01 (87.9% for MPOPs‐50k and 89.5% for MPOPs‐124), indicating that the MPO stage does not sacrifice sequence diversity while optimizing the molecular properties of AMP candidates. The analysis of sequence alignment (Figure S15, Supporting Information) also indicates that AMP candidates generated by MPOGAN display outstanding diversity and novelty.

### 2.5 Quantitative Analysis of MPO Stage

The main advantage of MPOGAN is that it optimizes multiple desired properties of AMP candidates in the MPO stage. In this stage, the model‐embedded screening is employed to obtain high‐quality AMP candidates from MPOGAN‐generated peptides at each iteration. To comprehensively assess the effectiveness of the MPO stage, we evaluated changes in four AMP candidate sets iteratively obtained in the model‐embedded screening throughout the MPO stage: (1) candidates generated by the MPOGAN generator ($X_{G_{\theta }}^{\tau }$), (2) candidates screened by the antimicrobial activity predictor ($X_{A}^{\tau }$), (3) candidates screened by the cytotoxicity predictor ($X_{AT}^{\tau }$), and (4) candidates screened by the de‐redundancy module ($X_{ATD}^{\tau }$). The iteration of MPOGAN optimization was performed for 700 epochs.

To assess the efficiency of the MPO stage, we analyzed the change in the number of AMP candidates passing every evaluator (Figure S5, Supporting Information). Specifically, the number of $X_{G_{\theta }}^{\tau }$, $X_{A}^{\tau }$, and $X_{AT}^{\tau }$ show a gradual downward trend during the iteration, with decreasing descent magnitude, indicating that the MPOGAN is gradually reaching convergence. The number of $X_{G_{\theta }}^{\tau }$ shows the most significant decrease (70.87%), indicating that the time consumption of the MPO stage is effectively controlled. In other words, as the iteration progresses, the MPOGAN generator needs to generate fewer AMP candidates to meet the requirements of the model‐embedded screening. Meanwhile, the number of $X_{ATD}^{\tau }$ remains stable across all iterations. These observations suggest that the RTKU strategy not only ensures that the amount of candidates meets the requirements for updating the dynamic dataset but also effectively controls the quantity of $X_{G_{\theta }}^{\tau }$, thus maximizing the efficiency of the plug‐and‐play model‐embedded screening.

To assess the fine‐tuning effect of MPOGAN during the MPO stage, we analyzed the percentage change of AMP candidates passing each evaluator relative to $X_{G_{\theta }}^{\tau }$ (Figure 3g). There is a consistent increase in the percentage of sequences passing through different model‐embedded evaluators with each iteration. Specifically, the percentage of AMP candidates passing the antimicrobial activity predictor ($X_{A}^{\tau }/X_{G_{\theta }}^{\tau }$) improves by 25.14% (from 62.13% to 87.27%), the cytotoxicity predictor ($X_{AT}^{\tau }/X_{G_{\theta }}^{\tau }$) improves by 41.89% (from 27.20% to 69.09%), and the de‐redundancy module ($X_{ATD}^{\tau }/X_{G_{\theta }}^{\tau }$) improves by 44.74% (from 18.53% to 63.27%). These observations indicate that the MPO stage has significantly enhanced the performance of MPOGAN in generating multi‐property superior AMP candidates. We also observed that, in every iteration, the percentage of $X_{AT}^{\tau }/X_{G_{\theta }}^{\tau }$ (candidates meeting both antimicrobial activity and cytotoxicity requirements) significantly decreases compared to those before the cytotoxicity screening ($X_{A}^{\tau }/X_{G_{\theta }}^{\tau }$, i.e., candidates meeting only the activity requirements), reducing by approximately 35% (before MPO stage) and 18% (after MPO stage). This implies that a substantial portion (approximately 56% (before MPO stage) and 21% (after MPO stage)) of peptides meeting the antimicrobial activity criteria cannot satisfy the cytotoxicity requirements. This phenomenon confirms the necessity of optimizing the cytotoxicity of AMPs in generative methods, and it also demonstrates that the model‐embedded screening can effectively remove candidates that do not meet multi‐property optimization objectives. Furthermore, we evaluated the diversity and novelty of MPOGAN‐generated sequences during the MPO stage (Figure S6, Supporting Information). Diversity, measured using the normalized Simpson and Shannon indices, remained stable across checkpoints for the standard MPOGAN, while the variant without the CD‐HIT deduplication module (MPOGAN w/o CD‐HIT) showed a decline after epoch 260, highlighting the CD‐HIT module's role in maintaining diversity by preventing redundant sequences. The novelty was measured by BLAST E‐values against experimentally validated AMPs. It increased significantly during optimization from approximately 0.95 to 0.99 and eventually stabilized. This suggests that MPOGAN effectively avoids overfitting to high‐scoring patterns while progressively learning a diverse range of functional features. These results confirm MPOGAN's ability to generate diverse and novel AMP candidates effectively.

To further assess the robustness of the RTKU strategy, we also analyzed how the data composition of the dynamic training set changes throughout the iterations (Note S4 and Figure S4, Supporting Information). The results indicate that the generated peptides in the dynamic dataset quickly reach the expected quantity ratio with the experimentally validated AMPs and then maintain the dynamic equilibrium. In conclusion, the RTKU strategy not only enhances the iterative efficiency but also ensures the stability of knowledge updates.

To strengthen our findings, we further evaluated the changes in the data distribution of AMP candidates generated by MPOGAN during the MPO stage, as compared to the experimentally validated AMP dataset (Figure 3h,i; Figure S12, Supporting Information). We employed trained models in different iterations to generate AMP candidates, ranging from the 0th iteration (i.e., the pre‐trained MPOGAN) to the 700th iteration (i.e., the final MPOGAN). The model parameters are saved every 20 iterations, resulting in a total of 36 models. Each model generates 5000 AMP candidates. We employed ESM‐2 (esm2_t6_8M_UR50D)^[^ ^49^ ^]^ to extract feature representations of the experimentally validated AMPs and all AMP candidates. We then downscaled the feature representations to two dimensions using Principal Component Analysis (PCA).^[^ ^60^ ^]^ We utilized Jensen‐Shannon (JS) divergence^[^ ^61^ ^]^ to evaluate the similarity between the feature distribution of candidates generated by 36 models in the MPO stage and the experimentally validated AMPs, as well as the similarity to high‐quality experimentally validated AMPs (i.e., peptides in the experimentally validated AMPs dataset that satisfy *P*_AMP_ > 0.8 and *P*_Toxin_ < 0.7). The smaller the value of JS divergence (ranging from [0, 1]), the closer the two distributions are. As the iterations progressed, the similarity between MPOGAN‐generated candidates and the experimentally validated AMPs gradually decreased, while the similarity to high‐quality true AMPs gradually increased. These observations indicate that MPOGAN can effectively utilize high‐quality peptide information during fine‐tuning in the MPO stage, and disregard information that resembles experimentally validated AMPs but does not meet multi‐property requirements.

### 2.6 Ablation Studies on MPOGAN

The optimization direction and performance of MPOGAN largely depend on the model‐embedded evaluators. To validate the effectiveness of the three model‐embedded evaluators (antimicrobial activity predictor (*f*_AMP_), cytotoxicity predictor (*f*_Toxin_), and de‐redundancy module (*f*_DR_)), we conducted comprehensive ablation studies. Specifically, we evaluated the impact of five evaluator combinations on the performance of MPOGAN. These combinations and their corresponding generative models include: (1) Pre‐trained MPOGAN without any evaluators; (2) Only *f*_AMP_ (*M*_A_); (3) Combines *f*_AMP_ and *f*_Toxin_ (*M*_AT_); (4) Combines *f*_AMP_ and *f*_DR_ (*M*_AD_); (5) Combines *f*_AMP_, *f*_Toxin_ and *f*_DR_ (*M*_ATD_, i.e., the final MPOGAN). All models were trained uniformly using identical hyperparameters, and each generated 50 000 AMP candidates for evaluation. We compared the percentages of AMP candidates that are non‐repetitive (*Unique*) and those not appearing in the experimentally validated AMP dataset (*Unseen*) for the five combinations (Figure S17c, Supporting Information). *M*_ATD_ achieved the best *Unique* and *Unseen* value. This indicates that the diversity and novelty of the AMP candidates are significantly improved during the MPO stage. We also observe that although *M*_A_ has a *Unique* value of 0.13% lower than pre‐trained MPOGAN, its *Unseen* value increased by 0.16%. This suggests that optimizing antimicrobial activity may slightly reduce the diversity of AMP candidates while increasing their novelty. Furthermore, the *Unique* values of *M*_AT_, *M*_AD_, and *M*_ATD_ have significantly increased compared to pre‐trained MPOGAN and *M*_A_. This indicates that the optimization of cytotoxicity and/or diversity completely offsets the negative impact of antimicrobial activity optimization on the diversity of AMP candidates. We further compared the minimum normalized within‐group edit distance distributions for the five combinations (Figure S17a, Supporting Information), and the inter‐group edit distance distributions relative to experimentally validated AMPs (Figure S17b, Supporting Information). The results indicate that combined evaluators can improve the diversity and novelty of generated AMPs.

We further compared the compositions of AMP candidates generated by the models corresponding to the five evaluator combinations mentioned above. We utilized LLM‐AAP and Toxinpred2^[^ ^19^ ^]^ to score these candidates, dividing them into four non‐overlapping groups: (1) Candidates that meet *P*_AMP_ ⩽ 0.8 and *P*_Toxin_ ⩾ 0.7, considered to neither satisfy antimicrobial activity nor cytotoxicity requirements (*Low‐quality* group); (2) Candidates that meet *P*_AMP_ > 0.8 and *P*_Toxin_ ⩾ 0.7, considered only to satisfy antimicrobial activity requirements, but not cytotoxicity (*Only‐AMP* group); (3) Candidates that meet *P*_AMP_ ⩽ and *P*_Toxin_ < 0.7, considered only to satisfy cytotoxicity requirements, but not antimicrobial activity (*Only‐toxin* group); (4) Candidates that meet *P*_AMP_ > 0.8 and *P*_Toxin_ < 0.7, considered to satisfy both antimicrobial activity and cytotoxicity requirements simultaneously (*High‐quality* group). Our goal is to obtain a larger proportion of the *High‐quality* group. We compared the composition of these four groups of AMP candidates generated by the five evaluator combinations (Figure 3j), where *M*_ATD_ achieves the best performance in the proportion of the *High‐quality* group. Compared to pre‐trained MPOGAN, the *High‐quality* group in *M*_A_ increased by 31.39%, and the *Only‐AMP* group increased by 44.93%. These findings suggest that *f*_AMP_ has no significant effect on the generation capacity of high‐quality AMP candidates. Merely optimizing antimicrobial activity is not sufficient to design AMPs with excellent properties. Compared to *M*_A_, the *High‐quality* group in *M*_AT_ increased by 70.83%, the *Only‐toxin* group increased by 34.58%, and the *Only‐AMP* group decreased by 50.43%. These observations illustrate that optimizing both antimicrobial activity and cytotoxicity can significantly improve the quality of AMP candidates, and significantly reduce the AMP candidates that only satisfy partial property requirements. The same conclusion can also be drawn by comparing *M*_AD_ with *M*_ATD_. Through the comparison between *M*_A_ and *M*_AD_, as well as between *M*_AT_ and *M*_ATD_, we observe that *f*_DR_ has no significant impact on the composition of the four groups. These observations indicate that optimizing sequence diversity can significantly enhance the diversity of AMP candidates while not affecting the quality of the AMP candidates. In conclusion, extensive ablation studies highlight that specialized optimization of antimicrobial activity, cytotoxicity, and diversity can effectively enhance the performance of MPOGAN in generating high‐quality AMP candidates.

To evaluate the effectiveness of the RTKU strategy, we conducted ablation experiments comparing the performance of MPOGAN with and without RTKU (referred to as MPOGAN w/o RTKU). First, we assessed the impact of RTKU on MPO efficiency by measuring the time required for sampling sequences from the generator during the MPO stage. In MPOGAN w/o RTKU, sequences generated by the model‐embedded screening pipeline were updated to the dynamic dataset in a rigid first‐in‐first‐out manner, leading to an unoptimized sampling time that stagnated at approximately 6 seconds per iteration and a total sampling time of 4027 seconds. In contrast, MPOGAN, which incorporates the RTKU strategy, dynamically adjusted the number of samples based on the dataset update needs, achieving a significantly reduced sampling time of approximately 2 seconds per iteration, with a total sampling time of 1388 seconds—a 66% reduction compared to MPOGAN w/o RTKU (Figure S18a, Supporting Information). These results demonstrate that the RTKU strategy significantly enhances MPO efficiency. Second, we investigated the role of RTKU in mitigating catastrophic forgetting by analyzing the sequence length entropy and distribution of generated candidates. MPOGAN‐generated AMP candidates exhibited greater sequence length entropy and flatter distribution patterns, whereas MPOGAN w/o RTKU demonstrated lower sequence length entropy, with a large proportion of sequences concentrated near the maximum length (25aa) (Figure 18b,c, Supporting Information). This indicates that without RTKU, the model forgets the knowledge from earlier stages while learning new knowledge. The RTKU strategy ensures the diversity of sequence length for the generated AMP candidates.

### 2.7 Wet‐Laboratory Validation

To further evaluate the MPOGAN‐generated AMP candidates, we conducted wet‐laboratory validation. The top ten high‐confidence peptides screened out through the preliminary selection pipeline (see “Methods” and Figure S16, Supporting Information), were synthesized via solid‐phase synthesis, and all purities are ⩾95%. All ten AMPs are novel and cannot be found in the existing UniProt^[^ ^62^ ^]^ database (Table S4, Supporting Information). We also synthesized a well‐known AMP, LL‐37,^[^ ^63^ ^]^ as a reference control due to its extensive clinical research for treating bacterial infections;^[^ ^22^ ^]^ and a low‐score peptide MPOP‐Neg selected from MPOPs‐50k, as a negative control

**Figure 4:**
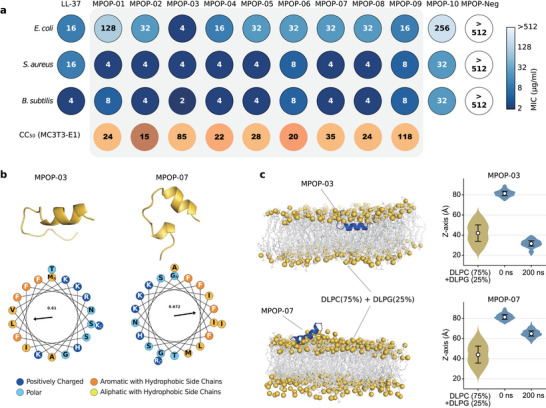
Wet‐laboratory validation and molecular dynamics simulation of the MPOGAN‐generated AMPs. a) MIC values of synthesized AMPs (x‐axis) tested against pathogens including *E. coli*, *S. aureus*, and *B. subtilis* measured in duplicates (n=3 independent experiments), as well as CC_50_ of synthesized AMPs (x‐axis) tested against MC3T3‐T1 cells. b) 3D structures of two potent AMPs (MPOP‐03 and MPOP‐07), obtained from 200 ns all‐atom molecular dynamics (MD) simulations in 20% TFE solution starting from an unstructured coil conformation (top), along with their α‐helical wheels (bottom). c) Left: 3D structures of MPOP‐03 and MPOP‐07 obtained from 200 ns all‐atom MD simulations in a membrane‐mimicking environment (75% DLPC and 25% DLPG bilayer), starting with the peptides initially placed above the simulated bacterial membrane in an α‐helical conformation. Right: Density comparison along the z‐axis between 0 and 200 ns during MD simulations of MPOP‐03 and MPOP‐07.

To evaluate the antimicrobial activity, we tested the minimum inhibitory concentrations (MICs) of these 12 peptides (Table S3 and Figures 23–25, Supporting Information) against Gram‐negative bacteria *E. coli* and two Gram‐positive bacteria, *S. aureus* and *B. subtilis* (see “Methods”). A lower MIC value indicates a stronger bacterial inhibition effect. All ten positive peptides exhibit potent antimicrobial activities with MIC values of ⩽256µg/mL, while the MPOP‐Neg shows MIC values of ⩾512µg/mL (**Figure** **4**a). All of these peptides show MIC values of ⩽32µg/mL against *S. aureus* and *B. subtilis*. These observations indicate that the peptides generated by MPOGAN have broad‐spectrum antimicrobial potential.

To evaluate the cytotoxicity, we tested the half maximal cytotoxic concentration (CC_50_) of nine AMPs (MPOP‐01 to MPOP‐09) against MC3T3‐E1 cells (See “Methods” and Figure S22, Supporting Information). These nine peptides exhibit a better inhibitory effect than LL‐37 against some pathogens. All these nine MPOPs exhibit CC_50_/MIC⩾2.5 against *S. aureus* and *B. subtilis*, suggesting that the peptides generated by MPOGAN have the potential to treat Gram‐positive bacterial infections without damaging eukaryotic cells.

Furthermore, to study the structure of MPOGAN‐generated AMPs and the mechanism of membrane‐peptide interactions, we predicted 3D structures of the two most promising AMPs, MPOP‐03 and MPOP‐07, using AlphaFold3^[^ ^64^ ^]^ (Figures S19 and S20, Supporting Information). We then conducted two molecular dynamics (MD) simulations for MPOP‐03 and MPOP‐07 using Gromacs 2024.2^[^ ^65^ ^]^ software (see Note S5, Supporting Information). First, starting from an unstructured coil conformation, we performed 200 ns MD simulations in 20% TFE solution. Both MPOP‐03 and MPOP‐07 exhibited stable α‐helical conformations at the end of the simulation (Figure 4b top). The α‐helical wheels (Figure 4b bottom) show strong amphiphilicity: positively charged amino acids (H, K, R) and polar amino acids (N, Q, S, T) aggregate to form the hydrophilic side, and hydrophobic amino acids (A, I, L, V, M, F, Y) aggregate to form the hydrophobic side. This amphiphilic distribution enables AMPs to selectively bind and disrupt bacterial cell membranes, enhancing their antimicrobial efficacy. Second, to investigate the mechanism of membrane‐peptide interaction, we performed 200 ns MD simulations in a membrane‐mimicking environment, starting with the peptides in an ‐helical conformation initially placed above a simulated bacterial membrane (75% DLPC and 25% DLPG bilayer). During the simulations, both peptides gradually moved closer to the bacterial membrane and eventually embedded themselves into the membrane (Figure 4c; Figure S21, Supporting Information). The results indicate that both MPOP‐03 and MPOP‐07 can maintain a stable α‐helical structure in TFE solutions or membrane‐mimicking environments.

**Figure 5:**
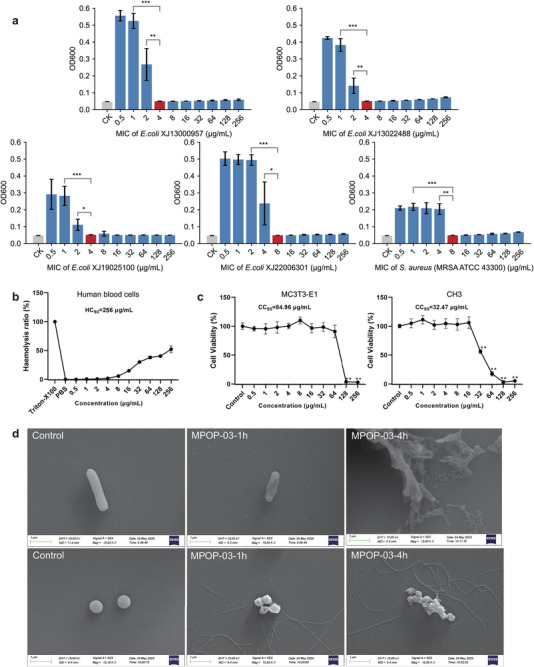
Biological properties of the most promising AMP candidate (MPOP‐03) in vitro. a) MIC values of MPOP‐03 against five drug‐resistant pathogens. Red bars represent MIC values, and CK (control check, grey bars) indicates blank medium. b) Hemolysis assay of MPOP‐03 on human blood cells. c) Cytotoxicity assay of MPOP‐03 on mouse osteoblasts MC3T3‐E1 (left) and bone marrow mesenchymal stem cells CH3 (right). d) Morphological changes of *E. coli* BL21(DE3) (top) and *S. aureus* BNCC186335 (bottom) were examined by scanning electron microscopy at 1 and 4 h post‐treatment with MPOP‐03. Scale bar =1 µm. All data are shown as the mean ± standard deviation (s.d.) from three independent experiments. P‐values were calculated using one‐way ANOVA. *: *p* < 0.05, **: *p* < 0.01, and ***: *p* < 0.001.

Due to its potent antimicrobial activity and low cytotoxicity, MPOP‐03 was subjected to further experimental validation. First, to study its broad‐spectrum antimicrobial effects against resistant bacteria, we assessed the MIC of MPOP‐03 against clinically relevant drug‐resistant strains, including *E. coli* XJ13000957, *E. coli* XJ13022488, *E. coli* XJ19025100, *E. coli* XJ22006301, and *MRSA* ATCC43300 (**Figure** **5**a). MPOP‐03 exhibited potent antibacterial activity against these resistant strains, with MIC values ⩽8 µg/mL. Second, to study its hemolysis, we performed a human blood hemolysis assay using various concentrations of MPOP‐03 (ranging from 0.5 to 256 µg/mL) (Figure 5b). The results indicated that the HC_50_ value (the concentration of MPOP‐03 required to induce 50% hemolysis of red blood cells) was determined to be 256 µg/mL. Additionally, the MIC of MPOP‐03 was measured as ⩽4 µg/mL against both *E. coli* BL21(DE3) and *S. aureus* BNCC186335, yielding a calculated selectivity index (SI) of ⩾64 in both cases. Generally, peptides with an HC_50_ ⩾100 µg/mL and an SI ⩾10 are regarded as having excellent safety profiles. These findings suggest that MPOP‐03 exhibits minimal hemolytic risk at its effective antimicrobial concentration. To evaluate its cytotoxicity on cell types critical for bone repair, we selected osteoblast MC3T3‐E1 and bone marrow mesenchymal stem cells CH3 (Figure 5c). The CC_50_ value for MC3T3‐E1 was determined to be 84.96 µg/mL, while for CH3, the CC_50_ value was found to be 34.27 µg/mL. Third, to study the membrane‐disrupting activity of MPOP‐03 against bacteria, scanning electron microscopy was utilized (Figure 5d). Unprocessed *E. coli* BL21(DE3) exhibited a characteristic short rod morphology, with rounded ends and a smooth, intact outer membrane surface. Following exposure to MPOP‐03 for 1 hour, cytoplasmic leakage was evident, and the cell walls degenerated into shrunken, empty husks. After 4 hours of treatment with MPOP‐03, most bacterial cells were reduced to fragmented membranes or unstructured debris, with surfaces appearing denatured and molten. Additionally, multiple lysed cells aggregated into clusters, obscuring individual cell boundaries and rendering them indistinguishable. Similarly, untreated *S. aureus* BNCC186335 displayed characteristic spherical morphologies with smooth surfaces and a consistent size distribution. Following 1 hour of exposure to MPOP‐03, localized disintegration of the *S. aureus* BNCC186335 cell wall was observed, leading to ring‐like damages featuring concave centers and elevated edges. After 4 hours of treatment with MPOP‐03, the cytoplasmic membranes of *S. aureus* BNCC186335 ruptured, resulting in a significant loss of intracellular contents, and numerous lysed cellular fragments aggregated to form amorphous masses with poorly defined boundaries.

## 3 Conclusion

In this study, we develop and establish MPOGAN, a novel GAN framework for de novo design of AMPs with potent antimicrobial activity, reduced cytotoxicity, and diversity. The MPOGAN framework consists of two learning stages, including the pre‐training stage and the multi‐property optimizing stage. The pre‐training stage enables MPOGAN to learn the general characteristics of experimentally validated AMPs. In the multi‐property optimizing stage, we constructed a dynamic dataset for the adversarial fine‐tuning of MPOGAN. We designed a model‐embedded screening process that combines multiple plug‐and‐play attribute evaluators, providing high‐quality generated AMP candidates with multiple desired properties for the dynamic dataset. We also proposed a robust real‐time knowledge‐updating strategy to guide the updating of the dynamic dataset, thereby encouraging MPOGAN to iteratively fine‐tune on new high‐quality data while avoiding forgetting the old ones. Through the two learning stages, MPOGAN enhances the ability to generate AMPs that meet the antimicrobial activity, cytotoxicity, and diversity requirements.

The key innovations of MPOGAN are threefold. First, it addresses multi‐property optimization with limited data by integrating pre‐trained property evaluators during the model‐embedded screening process. These evaluators abstract deep feature representations of individual properties, enabling the model to indirectly learn multi‐property characteristics without requiring large‐scale multi‐attribute datasets. This design mitigates overfitting while enhancing the generation of AMPs with superior therapeutic profiles. Second, the framework demonstrates plug‐and‐play extensibility, allowing seamless integration of new property evaluators without structural modifications. For instance, we incorporated a third‐party hemolysis evaluator (HemoPI2^[^ ^66^ ^]^) to create the MPOGAN+hemo variant, which achieved a 15.83% higher success rate in generating AMPs optimized for antimicrobial activity, cytotoxicity, and hemolysis (Figure S9, Supporting Information). Third, the Real‐Time Knowledge‐Updating (RTKU) strategy dynamically updates the training dataset by iteratively incorporating high‐quality generated AMP candidates while retaining experimentally validated AMPs, preventing the loss of prior knowledge and enabling continuous model refinement.

With these advancements, MPOGAN significantly outperforms baseline methods in generating AMPs de novo. Among the AMP candidates synthesized in the laboratory, nine out of ten exhibited broad‐spectrum antimicrobial activity and low cytotoxicity. Additionally, two of these candidates demonstrated broad‐spectrum antibacterial effects. These results validate the framework's ability to balance multiple therapeutic requirements while maintaining functional diversity.

The contributions of this study extend beyond antimicrobial peptide design. MPOGAN advances multi‐property optimization by overcoming data scarcity challenges, achieving a higher success rate in generating AMPs with desired attributes compared to state‐of‐the‐art methods. Its practical applicability is underscored by the high wet‐lab validation rate, bridging the gap between computational design and experimental implementation. Furthermore, the framework's modular architecture and dynamic updating protocol provide a generalizable framework for multi‐property optimization tasks. This extensibility aligns with the growing demand for adaptable AI tools in various scientific domains.

Despite its promising performance, MPOGAN has certain limitations. The current framework relies on curated datasets, which may still introduce subtle biases despite efforts to balance positive and negative samples. For instance, the distinction between experimentally validated AMPs and non‐AMPs may not fully capture the nuanced spectrum of antimicrobial activity. To address this, we have attempted to incorporate low‐activity peptides into the negative set, which improved the model's ability to distinguish between high‐activity and low‐activity peptides (Figure S10, Supporting Information). Further refinement of the dataset and property evaluators is necessary to enhance the robustness and generalizability of the framework. Additionally, while MPOGAN demonstrates strong computational performance, its practical utility requires further validation through extensive wet‐lab experiments, particularly for assessing long‐term stability, hemolysis, antimicrobial resistance, and other side effects of the generated AMPs. In the future, we aim to expand the framework by integrating additional property evaluators, such as those for hemolysis and half‐life, to address broader therapeutic requirements. We will prioritize experimental validation of more generated AMPs to ensure their translational potential and to refine the framework based on real‐world feedback. These efforts will contribute to the continued evolution of MPOGAN as a reliable tool for multi‐property AMP design and beyond.

## 4 Experimental Section

### Data Collection

Our work's training data comprises 30 812 peptide sequences, including an equal number of AMPs (15 406) and non‐AMPs (15 406). AMPs are collected from known AMP databases, and non‐AMPs are collected from the UniProt database.^[^ ^62^ ^]^ To ensure both the availability of training data and the practicality of peptide synthesis in the laboratory, we limit the selected peptides to a maximum length of 25 amino acids following existing studies.^[^ ^7^, ^10^ ^]^

For positive samples (i.e., experimentally validated AMPs), we collect experimentally validated peptides from SATPdb,^[^ ^67^ ^]^ CAMP_R4_,^[^ ^68^ ^]^ dbAMP,^[^ ^69^ ^]^ DRAMP,^[^ ^47^ ^]^ and LAMP^[^ ^70^ ^]^ databases. We remove duplicate peptides and those ⩾25 amino acids or containing non‐standard amino acids. Negative samples (i.e., non‐AMPs) are manually selected through the search criteria of UniProt.^[^ ^62^ ^]^ These criteria necessitate the subcellular location to be the cytoplasm and exclude any features such as antimicrobial, antibiotic, antiviral, antifungal, effector, and excreted.^[^ ^10^ ^]^ To increase the diversity of non‐AMPs, negative samples with ⩾40% sequence similarity are discarded and replaced with representative sequences from the cluster using CD‐HIT.^[^ ^50^ ^]^ Following the same method used in ref. [10], the negative samples retain the same length distribution and size as the positive samples, thus reducing the bias between the two sample types.

Furthermore, to train and evaluate the antimicrobial activity predictor, we divide the dataset into training, validation, and test sets in an 8:1:1 ratio. Each subset maintains an equal balance of positive and negative samples. The amino acid composition and length distribution of the dataset are shown in Figure S1 (Supporting Information).

### Antimicrobial Activity Predictor

To identify the highly potent AMP candidates, we construct an antimicrobial activity predictor, called LLM‐AAP, based on ESM‐2.^[^ ^49^ ^]^ As a ProLLM, ESM‐2 has demonstrated excellent performance in various downstream tasks.^[^ ^7^, ^25^, ^71^ ^]^ It leverages a BERT^[^ ^72^ ^]^ encoder and is trained with 150 million parameters unsupervised on the Uniref50^[^ ^73^ ^]^ dataset. The LLM‐AAP is modeled as a downstream task of the pre‐trained ESM‐2 encoder network. We employ ESM‐2 to encode peptides into feature representations, which are subsequently fed into a Multilayer Perceptron (MLP) to predict the probabilities of peptides being AMPs. In detail, the peptide sequence, denoted as *x* and with a length of *L* amino acids, is encoded by ESM‐2 to generate residue‐level representations with a dimension of (*L* + 2, *E*). Subsequently, an element‐wise average operation is performed across the length dimension to produce the global sequence representation with a dimension of (1, *E*). This global representation effectively captures the comprehensive features of the protein sequence. The sequence representations are further processed through three fully connected layers with rectified linear unit (ReLU)^[^ ^74^ ^]^ activations and batch normalization. The input layer of MLP has the same number of neurons as the output dimension from ESM‐2. The final layer of MLP uses a softmax function to enable class prediction. The LLM‐AAP employs an Adam optimizer and trains on binary cross‐entropy loss for 200 epochs. And the ESM‐2 model parameters remain fixed throughout the training process.

We employ the optimal hyperparameters obtained from the grid search, featuring a learning rate of 5 × 10^−5^, a batch size of 256, and three hidden layers within the MLP, containing 256, 128, and 64 neurons, respectively. We set the dropout rate at a consistent 0.1. LLM‐AAP was trained for a total of 200 epochs (Figure S3, Supporting Information).

### Pre‐training

MPOGAN is an extended GAN model comprised of a generator *G*_θ_ (Figure 1b) and a discriminator *D*_ϕ_ (Figure 1c), with model parameters represented as θ and ϕ respectively. Specifically, *G*_θ_ generates a sequence of amino acid tokens from a start token *s*_0_, while *D*_ϕ_ assesses whether the sequence is real. *G*_θ_ and *D*_ϕ_ engage in adversarial training until *G*_θ_ can generate sequences that are real enough that *D*_ϕ_ cannot distinguish whether they are real or generated sequences.

To enable MPOGAN to learn the features of experimentally validated AMPs and generate peptides similar to experimentally validated AMPs, we establish a pre‐training stage that includes: generator pre‐training, discriminator pre‐training, and adversarial learning of *G*_θ_ and *D*_ϕ_.

### Generator Pre‐Training

*G*_θ_ is a Recurrent Neural Network (RNN) with three layers. The first layer is a 3D embedding layer for each amino acid, leveraging word embeddings to capture semantic relationships among amino acids and reduce computational complexity. The next layer is a Gated Recurrent Unit (GRU) with 128 units. By introducing an update gate and a reset gate, the GRU retains historical information while controlling the impact of new inputs. Finally, the model ends with a fully connected layer using a softmax activation. This layer maps the GRU output to all possible amino acids, and softmax activation ensures these predictions sum to one, which can be interpreted as probabilities for each amino acid to be the next one in the sequence. We define the initial state of *G*_θ_ as *s*_0_ = 0.

We pre‐train *G*_θ_ on the experimentally validated AMP dataset, enabling it to have basic generative abilities. We also use maximum likelihood estimation to minimize the negative log‐likelihood loss. The Adam optimizer is employed to update the model parameters, with an initial learning rate of 0.0001. *G*_θ_ is trained for a total of 1000 epochs (Figure S3, Supporting Information).

### Discriminator Pre‐Training

*D*_ϕ_ functions as a binary classifier that distinguishes between real and generated sequences. Instead of additionally pre‐training a *D*_ϕ_ separately, we directly employ the parameters of the trained LLM‐AAP as ϕ.

### Adversarial Learning

To generate peptides that can deceive *D*_ϕ_, *G*_θ_ acts as an actor and outputs a complete peptide sequence *x* starting from *s*_0_ to maximize the expected cumulative reward. To avoid the differentiation difficulty associated with discrete data in conventional GAN, the *G*_θ_ is trained via policy gradient.^[^ ^48^ ^]^ We calculate the loss of *G*_θ_ as follows:

(1) $$J\left(\theta \right)=E\left[R|s_{0},\theta \right]=\sum_{x\in X}G_{\theta }\left(x|s_{0}\right)D_{\phi }\left(x\right)$$

where *R* stands for the reward of a complete AMP sequence, and X stands for the entire sequence space. The reward function *R* is defined as the positive probability output of *D*_ϕ_ for the generated sequence.

The optimization objective of *D*_ϕ_ is to maximize the probability of accurately classifying both real and generated peptides. We calculate the loss of *D*_ϕ_ as follows:

(2) $$\min_{D}-E_{x∼p_{\text{real}}}\left[\log D_{\phi }\left(x\right)\right]-E_{x∼p_{G_{\theta }}}\left[\log\left(1-D_{\phi }\left(x\right)\right)\right]$$

where *p*_real_ stands for the distribution of experimentally validated AMPs, and $p_{G_{\theta }}$ stands for the distribution of *G*_θ_. In our work, the adversarial learning process is conducted for 1000 epochs (Figure S3, Supporting Information).

### Multi‐Property Optimizing

To further enhance the ability of the pre‐trained MPOGAN to generate AMP candidates with multiple desired properties, we establish a multi‐property optimizing (MPO) stage, including model‐embedded screening, Real‐Time Knowledge‐Updating (RTKU), and adversarial fine‐tuning. Model‐embedded screening is proposed to identify high‐quality peptides that meet the multiple desired properties from generated AMP candidates. The RTKU strategy implements an extended feedback‐loop mechanism, which combines AMP candidates screened from model‐embedded screening and a random set of experimentally validated AMPs. It further updates these two sets into the dynamic dataset and adjusts the size of the AMP candidates set fed into the model‐embedded screening in the next iteration. Adversarial fine‐tuning is designed to enable MPOGAN to iteratively learn the features of high‐quality AMP candidates on dynamic datasets, improving the performance of generating multiple desired properties.

### Model‐Embedded Screening

The prerequisite for fine‐tuning MPOGAN on the dynamic dataset is to ensure data quality. In our work, we introduce model‐embedded screening to address this challenge.

When the τth iteration begins, a total of *N*_τ_ peptides (*x*) are sampled from *G*_θ_. We abstract these peptides as a set $X_{G_{\theta }}^{\tau }=\left\{x_{1},x_{2},\text{…},x_{N_{\tau }}\right\}$. Deep generative models are highly susceptible to the impact of noise interference.^[^ ^39^ ^]^ Therefore, the peptides utilized to update the dynamic dataset should be of high confidence to avoid unexpected bias. To screen out high‐quality AMP candidates from $X_{G_{\theta }}^{\tau }$, we consider the antimicrobial activity, cytotoxicity, and diversity as the key properties to be optimized.

First, we employ the antimicrobial activity predictor (*f*_AMP_) to screen the antimicrobial activity of $X_{G_{\theta }}^{\tau }$, given its fundamental importance for AMPs. Candidates with a predicted probability of antimicrobial activity >0.8 are retained.

Second, the avoidance of toxic peptides, which poses a significant challenge in AMP research,^[^ ^75^ ^]^ necessitates further prediction of cytotoxicity for qualifying peptides. We employ ToxinPred2^[^ ^19^ ^]^ (see Note S1.1, Supporting Information) as the cytotoxicity predictor (*f*_Toxin_). Candidates with a predicted probability of cytotoxicity <0.7 are retained.

Third, to remove redundancy from candidates and enrich the diversity of the dynamic dataset, we utilize CD‐HIT^[^ ^50^ ^]^ (see Note S1.2, Supporting Information) as the de‐redundancy module, represented as the de‐redundancy function *f*_DR_. Representative candidates with sequence similarity <60% are retained.

Finally, we define the model‐embedded screening as follows:

(3) $$X_{\text{hq}}^{\tau }=f_{\text{DR}}\left(f_{\text{Toxin}}\left(f_{\text{AMP}}\left(X_{G_{\theta }}^{\tau }\right)\right)\right)$$

where $X_{\text{hq}}^{\tau }$ stands for the high‐quality AMP candidates outputted from model‐embedded screening in the τth iteration. By embedding the frozen, pre‐trained evaluators directly into the screening process, the model‐embedded screening process ensures that only generated peptides meeting strict activity and toxicity criteria are used to update the dynamic dataset. This process maintains data quality and avoids the biases that can arise from adjusting evaluator parameters during training.

### Real‐Time Knowledge‐Updating Strategy

In the τth iteration of the MPO stage, we define the dynamic dataset as $X^{\tau }$, and the peptides to be updated as $X_{n}^{\tau }$. To ensure the robustness of the update strategy, we fix the size of the dynamic dataset to 1000 ($\left|X^{\tau }\right|=1000,\;\tau \geq 0$), and the size of the peptides to be updated to 250 ($\left|X_{n}^{\tau }\right|=\left|X_{n}\right|=250,\;\tau \geq 1$). Specifically, $X^{0}$ is randomly initialized with the experimentally validated AMP dataset ($X_{\text{real}}$). The RTKU strategy for $X^{\tau }$ adheres to the first‐in‐first‐out (FIFO) principle:

(4) $$X^{\tau }=X^{\tau -1}⊖X_{o}^{\tau -1}⊕X_{n}^{\tau },\;\tau \geq 1$$

where ⊖ stands for the set difference operation, ⊕ stands for the set union operation, and $X_{o}^{\tau -1}$ ($\left|X_{o}^{\tau -1}\right|=250,\tau \geq 1$) stands for the oldest updated peptides in $X^{\tau -1}$. More details of the update strategy of $X^{\tau }$ can be found in Note S1.3 and Figure S2 (Supporting Information).

Similar to incremental learning (IL),^[^ ^76^ ^]^ the original feedback‐loop mechanism in FBGAN^[^ ^36^ ^]^ continuously extracts knowledge from the new data generated by GAN. However, this paradigm may lead to two problems: (1) Mode collapse. This occurs when the GAN generates a limited variety of outputs, often producing the same or very similar results repeatedly, which reduces the diversity of generated peptides. The homogenization of peptides updated into the dynamic dataset makes it difficult for FBGAN to learn diverse knowledge and generate diverse peptides. (2) Catastrophic forgetting. Directly optimizing the network with new knowledge will erase the knowledge from earlier stages and result in irreversible performance degradation.^[^ ^77^ ^]^ In this study, we address the mode collapse problem by introducing a de‐redundancy module in the model‐embedded screening. To address the catastrophic forgetting problem, the RTKU strategy fine‐tunes MPOGAN using both new data from peptides generated by MPOGAN and old data from $X_{\text{real}}$, inspired by replay‐based IL.^[^ ^76^ ^]^ Specifically, we introduce a hyperparameter α = 0.7 to balance the relative size of new data ($X_{nn}^{\tau }$) and old data ($X_{no}^{\tau }$) in $X_{n}^{\tau }$:

(5) $$X_{nn}^{\tau }=\left\{x_{i}\in X_{\text{hq}}^{\tau }\;|\;1\leq i\leq \min\left(\left|X_{\text{hq}}^{\tau }\right|,\alpha \left|X_{n}\right|\right)\right\}$$

(6) $$X_{no}^{\tau }=\left\{x_{i}\in X_{\text{real}},x_{i}\notin X_{nn}^{\tau }\;|\;1\leq i\leq \left|X_{n}\right|-\left|X_{nn}^{\tau }\right|\right\}$$

(7) $$X_{n}^{\tau }=X_{nn}^{\tau }⊕X_{no}^{\tau }$$

To ensure the robustness and efficiency of the RTKU strategy, *N*_τ_ is designed as a value that varies with iteration to ensure that model‐embedded screening outputs a sufficient amount of new knowledge while minimizing screening time as follows:

(8) $$N_{\tau +1}=\frac{\alpha \left|X_{n}\right|}{\left|X_{\text{hq}}^{\tau }\right|}N_{\tau }$$

### Adversarial Fine‐Tuning

The adversarial fine‐tuning of MPOGAN is performed on the dynamic dataset $X^{\tau }$, with all sequences in the dataset considered positive samples. The training of *G*_θ_ and *D*_ϕ_ is consistent with the pre‐training stage. *G*_θ_ is trained to generate high‐quality AMP candidates, while *D*_ϕ_ is trained to distinguish AMPs and non‐AMPs.

Through repeated iterations of model‐embedded screening and adversarial fine‐tuning, MPOGAN continuously learns the features of AMP candidates with potent antimicrobial activity, low cytotoxicity, and high diversity simultaneously. On the one hand, *D*_ϕ_ learns the features of high‐quality AMP candidates, which leads to a stricter definition of “positive samples” and continuous updates to the parameters ϕ, resulting in more precise discrimination of the sequences generated by *G*_θ_. On the other hand, *G*_θ_ continuously updates the parameters θ based on the feedback from *D*_ϕ_, optimizing the generation preferences to generate peptides that are more similar to the dynamic dataset, i.e., peptides that possess high‐quality features. In the MPO stage, a total of 700 epochs of iterative training were conducted (Figure S3, Supporting Information).

### Preliminary Selection of Potential Candidates for Wet‐Laboratory Validation

We established a preliminary selection pipeline (Figure S16, Supporting Information) to select the most promising AMP candidates generated by MPOGAN for wet‐laboratory validation, thereby saving experimental costs.

Specifically, for the AMP candidates generated by MPOGAN, we first remove the sequences that already exist in the experimentally validated AMP dataset. To preliminarily exclude sequences that do not conform to expert experience, we perform the following biological experience screening:^[^ ^4^, ^10^ ^]^ (1) screening AMP candidates with a positive net charge; (2) excluding AMP candidates in which there are more than three positively charged amino acids (K, R) in a window of five amino acids; (3) excluding AMP candidates in which occur three hydrophobic amino acids in a row. We consider amino acids as hydrophobic based on the Eisenberg scale: A, V, L, I, M, F, W. To further ensure the key properties of AMP candidates, we introduce the auxiliary classifier screening. In detail, to ensure that the AMP candidates have high antimicrobial activity, we filter the AMP candidates using multiple antimicrobial activity predictors, including LLM‐AAP, AMPScanner v2^[^ ^17^ ^]^ and AMPlify.^[^ ^18^ ^]^ We retain sequences with a probability of being an AMP ⩾0.99 predicted by all three predictors. To ensure that the AMP candidates have low cytotoxicity, we filter the sequences using the cytotoxicity predictor ToxinPred2,^[^ ^19^ ^]^ retaining sequences with a cytotoxicity probability ⩽0.5. To obtain sequences containing the α‐helix conformation, which is a common structure of AMPs known for their high activity, stability, amphipathicity, and clear membrane action mechanisms, we filter the sequences using the HeliQuest^[^ ^78^ ^]^ web server. The parameters are set as follows: Hydrophobicity: ‐1 ⩽*H* ⩽ 0.6; Hydrophobic moment: 0.4 ⩽*M* ⩽ 1.2; Net charge: 0 ⩽*Ch* ⩽ 5; Minimal number of polar residues + glycine: 0; Minimal number of uncharged residue: 1; Minimal number of glycine: 0; Maximal number of charged residue: 12; Is proline accepted? (at i, i+3 / n‐3, n): Yes; Is cysteine accepted?: Yes.

Finally, to select the most promising AMP candidates for wet‐laboratory validation, we calculate the weighted score (*S*) for each sequence (*x*). *S* is designed to prioritize sequences with higher predicted antimicrobial activity, lower toxicity, shorter length, and a higher proportion of α‐helix structure—all desirable features in effective and safe AMPs. The weighted score is calculated as follows:

(9) $$S=\frac{1}{3}\left(Avg\left(f_{\text{AMP}}\left(x\right)\right)+\left(1-f_{\text{Toxin}}\left(x\right)\right)+L\right)$$

where *Avg*(*f*_AMP_(*x*)) stands for the average score of the three antimicrobial activity predictors (LLM‐AAP, AMP Scanner v2, AMPlify), *f*_Toxin_(*x*) stands for the score of the cytotoxicity predictor. The length evaluation score (*L*) integrates sequence compactness and helical content through a normalized metric:

(10) $$L=\frac{1}{2}\left(\left(1-\frac{l}{L_{\max}}\right)+\frac{h}{l}\right)$$

where *l* stands for the length of *x*, *h* stands for the length of the predicted α‐helix segment in *x*, and *L*_max_ stands for the maximum sequence length in the AMP candidate set (25 amino acids, derived from the training data distribution and generation constraints of MPOGAN). The term $1-\frac{l}{L_{\max}}$ penalizes longer sequences on a normalized scale [0, 1), favoring shorter peptides to reduce synthesis costs and potential toxicity. The term $\frac{h}{l}$ rewards sequences with higher helical content relative to their total length, emphasizing structural functionality.

For the 50 000 AMP candidates generated from MPOGAN, the number of candidates is reduced to 49 538 after de‐duplication. Then, with biological experience screening,^[^ ^4^, ^10^ ^]^ the candidate pool is further decreased to 18,153. Next, auxiliary classifier screening is conducted, and the number of candidates declines to 429. After further evaluating the secondary structure, we leave 124 peptides (Table S5, Supporting Information) as stringent AMP candidates for physicochemical features analysis. Finally, we select the top 10 candidates with the highest weighted score for wet‐laboratory validation (Figure S16, Supporting Information).

### Materials

Dulbecco's Modified Eagle Medium (DMEM) was obtained from Biosharp (Beijing, China), and fetal bovine serum (FBS) was sourced from VivaCell (Shanghai, China). Dimethyl sulfoxide (DMSO) and Triton X‐100 were purchased from Shanghai Aladdin Reagent Co., Ltd. *E. coli* BL21(DE3) was acquired from Tsingke Biotechnology (Beijing, China). *S.aureus* BNCC186335 and *B.subtilis* BNCC109047 strains were provided by the Beijing National Culture Collection (BNCC, Beijing, China). Clinical isolates, including drug‐resistant strains *E. coli* XJ13000957, *E. coli* XJ13022488, *E. coli* XJ19025100, and *E. coli* XJ22006301, were obtained from the clinical laboratory of Xijing Hospital (Xi'an, China). Methicillin‐resistant *Staphylococcus aureus* (MRSA) ATCC 43300, mouse osteoblast MC3T3‐E1 cells, and bone marrow mesenchymal stem cells C3H were sourced from the American Type Culture Collection (ATCC, USA). Baird‐Parker agar was purchased from Sangon Biotech (Shanghai) Co., Ltd. Human whole blood samples were obtained from Huizhi Heyuan Biotechnology (Suzhou) Co., Ltd. All other reagents used in this study were of analytical grade and were utilized without further purification.

### Antimicrobial Activity Assay

The antimicrobial activity of the synthesized AMPs was evaluated against several strains, including *E. coli* BL21(DE3), *S. aureus* BNCC186335, *B. subtilis* BNCC109047, and drug‐resistant strains such as *E coli* XJ13000957, *E. coli* XJ13022488, *E. coli* XJ19025100, *E. coli* XJ22006301, and MRSA ATCC43300. All strains are stored at ‐80°C and, before testing, were transferred into fresh Mueller‐Hinton Medium and incubated for 24 h at 37°C. Fresh cultures were used for the antimicrobial assays. The MIC values were determined using the broth microdilution method according to the Clinical and Laboratory Standards Institute (CLSI) protocol. Initial bacterial inoculums (5× 10^5^ colony forming units (CFU)/mL) in Mueller‐Hinton Broth were exposed to varying concentrations of the compounds (0.5‐512 µg/mL) and incubated for 18 h at 37°C. The experiments are conducted on 96‐well microtiter plates with a final volume of 100 µL. The MIC was defined as the lowest concentration of the compound that inhibits visible bacterial growth. Additionally, the MBC was evaluated. After determining the MIC, samples from the MIC well and two higher concentrations are plated on agar plates. The MBC was recorded as the lowest concentration at which no visible bacterial colonies were observed. All experiments were performed in triplicate, with positive controls (ensuring proper bacterial growth) and negative controls (ensuring sterility) included.

### Cytotoxicity Assay

The assessment of cytotoxicity primarily relies on alterations in cell membrane permeability. To evaluate the cytotoxicity of AMPs, we employed the Cell Counting Kit‐8 (CCK‐8) assay, which is widely recognized as one of the most frequently used methods for assessing cytotoxicity. The CCK‐8 assay utilizes WST‐8 [2‐(2‐Methoxy‐4‐nitrophenyl)‐3‐(4‐nitrophenyl)‐5‐(2,4‐disulfophenyl)‐2H‐tetrazolium Sodium Salt] for rapid detection based on the principle that intracellular dehydrogenase‐mediated biological reduction of WST‐8 generates an orange formazan dye. This dye can dissolve in the medium, and its quantity is directly proportional to the number of viable cells. For the cytotoxicity test, 2 000 MC3T3‐E1 cells or CH3 cells were seeded into each well of 96‐well plates and cultured in DMEM medium containing 10% FBS and 1% penicillin‐streptomycin at 37 °C and 5% CO_2_overnight. Then the culture medium was removed, and the AMPs at the various concentrations in the media were added to each well. The cells were further incubated for 48 h, followed by the addition of 20 L of CCK‐8 and subsequent incubation for 1 h. The absorbance of the solution was measured at 450 nm by a microplate reader. The cell viability was calculated as follows (A represents absorbance):

(11) $$\%\text{Cell viability}=\frac{A_{\text{sample}}-A_{\text{blank}}}{A_{\text{negative}}-A_{\text{blank}}}\times 100$$

The data were plotted as AMP concentration versus the percentage of living cells using GraphPad Prism 8.0.

### Hemolysis Assay

Anticoagulant whole blood was diluted with PBS at a ratio of 1:1 and subsequently centrifuged at 1500×g for 10 minutes. The supernatant and white blood cell layer were discarded, while the red blood cells were washed three times with PBS until the supernatant became colorless and transparent. A 2% red blood cell suspension was then prepared using PBS. For the positive control, 0.2 mL of 1% Triton X‐100 was mixed with 0.8 mL of the red blood cell suspension. The negative control consisted of 0.2 mL of PBS combined with 0.8 mL of the red blood cell suspension. In the experimental group, 0.2 mL of MPOP‐03 (prepared at gradient concentrations) was added to 0.8 mL of the red blood cell suspension. Three replicates were prepared for each group. All samples were incubated at 37°C in a constant temperature water bath for 3 hours. Following incubation, the samples were centrifuged at 1500×g for 5 minutes, and the supernatants were carefully aspirated and transferred to new centrifuge tubes. The optical density values of the samples were measured at a wavelength of 524 nm using a microplate reader. The hemolysis ratio was calculated as follows (A represents absorbance):

(12) $$\%\text{Hemolysis ratio}=\frac{A_{\text{sample}}-A_{\text{negative}}}{A_{\text{positive}}-A_{\text{negative}}}\times 100$$

### Morphology Observation by SEM

The *E. coli* BL21(DE3) and *S. aureus* BNCC186335 strains were cultured until they reached the logarithmic growth phase. The bacteria were then harvested by centrifugation at 8000×g for 10 minutes, resuspended in culture medium, and adjusted to a concentration of 10 CFU/mL. For the experimental group, four times the MIC of the antimicrobial peptide was added, followed by incubation at 37°C for 1 hour and 4 hours. In the control group, an equal volume of PBS was added, and samples were incubated under identical conditions. The samples were then fixed with 4% paraformaldehyde overnight. They were dehydrated in 30%, 50%, 70%, 80%, and 90% ethanol for 10 minutes each, and finally dehydrated in 100% ethanol three times, each for 15 minutes. The ethanol was replaced with isopentyl acetate, and the bacteria were placed on a silicon wafer. The dried samples were adhered to a sample stage fixed with conductive tape and gold‐coated. The morphological characteristics of the bacteria were characterized by scanning electron microscopy (Zeiss Gemini 500 Field Emission Scanning).

### Statistical analysis

All statistical analyses and visualizations were performed using Python (v3.8.0) and GraphPad Prism 8.0. In violin box plots, the white dots indicate the median of each distribution, and the black short lines denote the interquartile range of each distribution. Sample sizes (n) are provided in the figure legends. Statistical significance is indicated by *p*‐values, denoted as *p < 0.05, ** *p* < 0.01, and *** *p* < 0.001, calculated using one‐way ANOVA. Error bars represent standard deviations.

## Conflict of Interest

The authors declare no conflict of interest.

## Supporting information

Supporting Information

## Data Availability

The data that support the findings of this study are available in the supplementary material of this article.
