# Supplementary material for: A Multi‐Property Optimizing Generative Adversarial Network for de novo Antimicrobial Peptide Design
Source: Adv Sci (Weinh). 2025 Aug 11;12(38):e03443. doi: 10.1002/advs.202503443 (PMC12520537; doi:10.1002/advs.202503443)
Supplement: Supplementary file 1 — Supporting Information [file ADVS-12-e03443-s001.pdf]

ADVANCED SCIENCE
Open Access

Supporting Information

for *Adv. Sci.*, DOI 10.1002/advs.202503443

A Multi-Property Optimizing Generative Adversarial Network for de novo Antimicrobial Peptide Design

Jiaming Liu, Tao Cui, Tao Wang, Lishan Lin, Xi Zeng, Dazhi Lu, Shaoqing Jiao, Jun Wang, Xiaoyan Li, Shuyuan Xiao, Dongna Xie, Xuecheng Wang, Yongtian Wang, Xuequn Shang, Yinbo Niu, Zhongyu Wei* and Jiajie Peng**

Supplementary information of
A Multi-Property Optimizing Generative Adversarial Network for de novo Antimicrobial Peptide Design

Contents

Supplementary Note 4
1 Supplementary Methods 4
1.1 Details of the cytotoxicity predictor 4
1.2 Details of the sequence de-redundancy module 4
1.3 More information on the Real-Time Knowledge-Updating (RTKU) strategy 4
2 Baseline methods and evaluation settings for AMP generation task 5
2.1 Baseline methods 5
2.2 Evaluation settings 7
3 Baseline methods and evaluation settings for AMP identification 8
3.1 Baseline methods 8
3.2 Evaluation settings 10
4 Robustness assessment of MPO stage 10
5 Molecular dynamic simulations 11
5.1 Molecular dynamics simulation of unstructured coil conformation AMPs in TFE solution 11
5.2 Molecular dynamics simulation of AMPs in membrane-mimicking environment 11
Supplementary Tables 13
1 Performance evaluation of AMP prediction methods on independent test set 13
2 Percentage of AMP candidates generated by (pre-trained) MPOGAN across different E-value categories 13
3 Sequence information and physicochemical properties of synthesized peptides 14
4 BLAST sequence similarity search of synthesized peptides in the UniProt database 15
5 AMP candidates information after preliminary selection 16
Supplementary Figures 19
1 Amino acid composition and length distribution of the AMP and non-AMP dataset 19
2 The real-time knowledge-updating strategy for the dynamic dataset 20
3 Training loss curve of MPOGAN (Pre-training, adversarial learning, and adversarial fine-tuning) 21
4 Changes in the composition of the dynamic training dataset during the MPO stage 22
5 Changes in the number of successfully filtered sequences in the model-embedded screening pipeline 23
6 Changes in diversity and novelty of the MPOGAN-generated sequences during the MPO stage 24
7 Supplementary comparative evaluation of MPOGAN and generative baseline methods on generating AMPs de novo 25
8 Supplementary comparative evaluation of MPOGAN and generative baseline methods on the comprehensive properties of generated AMPs 26
9 Analysis of plug-and-play extensibility of MPOGAN in hemolysis optimization 27
10 Performance comparison of MPOGAN and LLM-AAP after training on the improved dataset 28
11 Confusion matrix of methods for AMPs prediction on the independent test set 29
12 Visualization of experimentally validated AMPs and generated peptides from various training stages 30

13 Comparison of 5 physicochemical properties between MPOGAN generated sequences (MPOPs-50k, MPOPs-124) and experimentally validated AMPs 32
14 Supplementary analysis of the diversity and novelty of MPOGAN-generated sequences 33
15 Violin plots of match score distributions from peptide group comparisons 34
16 Preliminary selection pipeline before wet-experiment validation 35
17 Supplementary ablation studies of model-embedded evaluators for various combinations 36
18 Supplementary ablation studies for the RTKU strategy 37
19 Alpha-helical wheels of 12 synthesized peptides 38
20 3D structures of synthesized peptides predicted using AlphaFold3 39
21 Density changes along the Z-axis during peptide-membrane molecular dynamics simulations of MPOP-03 and MPOP-07 40
22 Cell viability of MPOPs on MC3T3-E1 at various concentrations 41
23 MIC values of 12 synthesized peptides 42
References 45

Supplementary Note

1 Supplementary Methods

1.1 Details of the cytotoxicity predictor

In our work, we utilized a hemolytic toxicity predictor to evaluate the cytotoxicity of peptides during the multi-property optimization (MPO) process, guiding MPOGAN to learn the features of peptides with low cytotoxicity. We employ the trained ToxinPred2 (*1*) as our cytotoxicity predictor. ToxinPred2 is an enhanced method designed to predict the toxicity of peptides and small proteins, advancing on the original ToxinPred (*2*). ToxinPred2 employs techniques including Basic Local Alignment Search Tool-based similarity, Motif-EmeRging and with Classes-Identification-based motif search, along with prediction models. By combining these strategies, ToxinPred2 achieves high accuracy, maintaining a balance between sensitivity and specificity.

1.2 Details of the sequence de-redundancy module

During the model-embedded screening process, it is crucial to constantly update the dynamic dataset in real-time, enabling MPOGAN to learn the features of high-quality peptides. However, if a peptide has excellent properties, it is likely that peptides with similar sequences have similar properties. This can result in a higher similarity between high-quality peptides identified by both the antimicrobial activity predictor and the cytotoxicity predictor during the model-embedded screening process. If we employ peptides screened by the initial two screening modules directly to update the dynamic dataset, it may diminish the diversity of high-quality peptides, leading to a homogenization of features learned by MPOGAN. To address this issue, we incorporate a sequence de-redundancy module as the final component of the model-embedded screening process. Given its impact on screening efficiency, we utilize CD-HIT (*3*) as the sequence de-redundancy module.

CD-HIT, an advanced program for biological sequence clustering, significantly speeds up the clustering process through a unique parallelization strategy and other enhancements. By streamlining core steps, implementing comprehensive parallelization with multiple threads, and integrating improvements like quicker file reading and advanced filtering, CD-HIT achieves notable acceleration. This enables efficient clustering of vast datasets in a fraction of the time compared to earlier versions.

1.3 More information on the Real-Time Knowledge-Updating (RTKU) strategy

The dynamic dataset is updated with iterations of the MPO stage. In our work, as shown in Supplementary Figure 1, the dynamic dataset consists of 1000 peptides, with the oldest 250 peptides being updated in each iteration. We divided the dataset into 4 groups of equal size, each containing 250 peptides. With this grouping, only the currently oldest sequence needs to be substituted with the latest peptides in each iteration. In the dynamic dataset, the entry time of each peptide is recorded and updated in sync with the dataset. Before the MPO stage, the dynamic dataset is initialized with 1000 randomly selected data from the experimentally validated AMPs, and the entry time of all the peptides is initialized to 0. Beginning from the first iteration, the oldest 250 peptides are removed in each round based on a first-in-first-out principle,

and subsequently replaced with 250 new peptides. The entry time of the newly added peptides is set to the current iteration. The entire dynamic dataset will be completely updated once every 4 iterations.

2 Baseline methods and evaluation settings for AMP generation task

2.1 Baseline methods

In the AMP generation task, we compared nine baseline methods, which are AMPEMO (*4*), Dean-VAE (*5*), Nagajaran-LSTM (*6*), PepCVAE (*7*), HydrAMP (*8*), Muller-RNN (*9*), PepGAN (*10*), AMP-GAN (*11*), FBGAN (*12*), AMP-Designer(*13*), MMCD(*14*), and PrefixProt(*15*).

AMPEMO. AMPEMO is an evolutionary multi-objective approach developed for the de novo design of AMPs. This method simultaneously optimizes two key objectives: antimicrobial activity and diversity among the identified AMPs. To achieve this, AMPEMO employs a deep learning model that accurately predicts a peptide's antimicrobial activity, and a niche sharing method that estimates a peptide's density in the sequence space to promote diversity. The approach utilizes a decomposition-based evolutionary multi-objective algorithm with an elite archive to efficiently search for AMPs with high activity and diversity. Additionally, a local search strategy is integrated to enhance the quality of the generated peptides. Experimental results demonstrate that AMPEMO outperforms state-of-the-art designs by generating various AMPs that possess high antimicrobial activities and are distinct from each other and from known AMPs in existing datasets.

Dean-VAE. Dean-VAE trains a variational autoencoder (VAE) on a database of known AMPs and their scrambled counterparts to generate a continuous latent space representation of AMPs. The encoder network converts peptide sequences into latent space vectors and the decoder network converts these vectors back into peptide sequences. New AMP sequences are generated by sampling points in the latent space near known active AMPs. The model’s capability to generate novel active AMPs is validated by testing the antimicrobial activity of interpolated sequences between known active and inactive peptides.

Nagajaran-LSTM. Nagajaran et al. implement a computational approach that employs a long short-term memory (LSTM) language model to design antimicrobial peptides. The LSTM model is applied to analyze the arrangement and frequencies of amino acid residues in known antimicrobial peptide sequences, interpreting them as words of a 20-alphabet language. Based on the output of the LSTM network, ten of LSTM-generated peptides are synthesized and tested against bacterial pathogens. The study demonstrates the effectiveness of this LSTM-based peptide design approach in generating peptides with broad-spectrum antimicrobial activity, including against multidrug-resistant clinical isolates of bacteria.

PepCVAE. PepCVAE is a semi-supervised VAE. This framework aims to design novel AMPs sequences by learning a rich latent space of biological peptide context from unlabeled peptide sequences. By leveraging feedback from a jointly trained AMPs classifier using limited labeled instances, the model further learns a disentangled antimicrobial attribute space, allowing for controllable generation of AMPs. The PepCVAE architecture demonstrates superior performance compared to a plain VAE by generating novel AMPs with higher long-range diversity while remaining closer to the training distribution of biological peptides.

Since the code, trained model, training data, and generated peptides for this work are not available, and Szymczak et al. (*8*) reproduced it successfully, the results reproduced by Szymczak et al. are used for comparison in our work.

HydrAMP. HydrAMP is a conditional variational autoencoder (cVAE) designed for AMPs generation. This model learns a lower-dimensional, continuous representation of peptides, disentangling their antimicrobial properties. It can generate analogues of existing peptides with specified antimicrobial conditions and also generate peptides de novo. HydrAMP leverages a continuous peptide representation with disentangled antimicrobial conditions, allowing for both analogue and unconstrained generation. The model benefits from a temperature parameter controlling creativity in analogue generation and has been trained specifically for the task of analogue generation, including generating both positive and negative analogues. In addition, the model introduces a preselection procedure based on external classifiers and molecular dynamics simulations, enhancing the experimental validation rate of the generated peptides.

Muller-RNN. Muller et al. developed a generative LSTM recurrent neural network (RNN) based on amino acid sequences of helical AMPs. The trained model was used for de novo sequence generation, resulting in 82% of the generated sequences predicted to be active antimicrobial peptides. Furthermore, the generated sequences were observed to be more similar to the training data than manually designed amphipathic helices. This showcases the potential of LSTM in constructing new amino acid sequences for peptide and protein design without the need for exhaustive enumeration of sequence libraries.

PepGAN. PepGAN is a peptide-specialized generative adversarial network (GAN) designed to generate AMPs with high activity. Unlike traditional GANs that aim to cover active peptides broadly, PepGAN introduces an activity-aware reward function that balances the generation of active peptides while avoiding non-active ones. This is achieved by integrating a separately trained activity predictor into the reward function, which guides the generator to produce sequences with desired antimicrobial properties. As a result, PepGAN exhibits superior statistical fidelity concerning physicochemical descriptors such as charge, hydrophobicity, and molecular weight. In experimental validation, six top peptides generated by PepGAN were synthesized, and one demonstrated exceptional antimicrobial activity with a MIC of 3.1 μg/mL, making it twice as potent as ampicillin.

AMP-GAN. AMP-GAN is a deep convolutional generative adversarial network (DCGAN) designed to generate novel AMPs. The model is trained on a dataset of known AMPs, encoded using a protein-encoding method called PC6, which transforms each peptide into a matrix that captures the order of amino acids and their physicochemical properties. By inputting random noise, the generator produces new peptide sequences. These generated peptides are first evaluated in silico using AI4AMP to predict their antimicrobial activity. From the generated candidates, eight peptides were selected and synthesized for experimental validation. Seven out of the eight synthesized peptides exhibited antibacterial activity. Notably, two peptides demonstrated broad-spectrum antibacterial effects, including effectiveness against antibiotic-resistant strains such as methicillin-resistant S. aureus and carbapenem-resistant P. aeruginosa.

FBGAN. FBGAN is a feedback-loop generative adversarial network developed to generate synthetic DNA sequences encoding proteins with desired properties, such as antimicrobial activity and specific secondary structures like α-helices. The architecture combines a Wasserstein GAN as the generator and an external function analyzer that evaluates and assigns favorability scores to the generated sequences. Unlike traditional GANs, FBGAN does not require the analyzer to be differentiable, allowing for the integration of any black-box predictor, including web servers or non-differentiable models. The feedback mechanism involves sampling sequences from the generator, scoring them with the analyzer, and incorporating high-scoring sequences back into the discriminator's training dataset. This iterative process guides the generator to produce sequences that are enriched for the desired properties. Applied to generate genes coding for AMPs and optimizing for α-helical content, FBGAN demonstrates its capability to produce synthetic genes resulting in proteins with desirable biophysical properties.

AMPDesigner. AMP-Designer is an innovative LLM-based framework for the rapid de novo design of AMPs with desired properties, integrating advanced techniques such as GPT-based foundation modeling, contrastive prompt learning, knowledge distillation, and reinforcement learning. By leveraging AMP-GPT and AMP-Prompt for transfer learning and efficient sequence generation, AMP-Designer excels in designing potent AMPs even with limited labeled data. Its plug-and-play design enables task-specific AMP generation within days, as demonstrated by the discovery of KW13 and AI18, two highly effective and low-toxicity AMPs validated in vitro and in vivo within 48 days. This framework represents a breakthrough in addressing antibiotic resistance through efficient and precise peptide design.

MMCD. MMCD (Multi-Modal Contrastive Diffusion) is an innovative generative model designed for therapeutic peptide generation, uniquely integrating sequence and structure modalities to co-generate residue arrangements and backbone coordinates. MMCD employs a novel inter-intra contrastive learning strategy at each diffusion timestep: inter-modal contrastive learning aligns sequence and structure embeddings to ensure consistency, while intra-modal contrastive learning differentiates therapeutic from non-therapeutic peptides to enhance generalization. This dual strategy enables MMCD to outperform state-of-the-art methods across key metrics such as antimicrobial/anticancer scores, diversity, and peptide-docking, demonstrating its superior capability in generating high-quality therapeutic peptides.

PrefixProt. PrefixProt is a controllable protein design method that leverages prefix-tuning to learn virtual tokens as control tags for specific protein properties, enabling precise and flexible prompting of pre-trained protein language models. Unlike traditional fine-tuning, PrefixProt optimizes significantly fewer parameters by freezing the ProtLM and training only the prefix tokens, making it especially effective in low-data settings. This approach allows for the generation of proteins with tailored structures and functions, including combinations of multiple properties (e.g., antimicrobial and anticancer functions). As the first method to apply prefix-tuning in protein sequence generation, PrefixProt represents an innovative step forward in drug discovery and protein engineering.

2.2 Evaluation settings

AMP candidates for comparison. For Dean-VAE, Nagajaran-LSTM, and Muller-RNN, we collected the generated AMP candidates provided in their Supplementary Materials; For HydrAMP and PepCVAE, we

used Supplementary Algorithm six of (8) to obtain their AMP candidates; For AMPEMO and PepGAN, we retrained the models using their open-source code and data; For AMP-GAN, Since the training data (https://symbiosis.iis.sinica.edu.tw/PC_6/data/example.txt) of this work are not available, we retrained this model using the training data from MPOGAN and generated 50,000 sequences for model comparison; For FBGAN, we used the training data from MPOGAN, train the model with our trained LLM-AAP as the functional analyzer, and generated 50000 sequences for model comparison. For AMP-Designer and PrefixProt, we utilized the AMP sequences provided in their publicly available datasets, each containing 1,000 generated AMP candidates. Additionally, we reproduced the MMCD and generated 1,000 AMP sequences for further comparison. Our evaluation for MPOGAN does not involve any screening processes but directly assesses the model-generated peptides. For MPOGAN, we sampled 50,000 peptides directly from the generator. To ensure a fair and consistent comparison, we excluded peptides longer than 25 amino acids and those containing non-standard amino acids from sequences generated by baseline models. The final number of AMP candidates we collected for comparison is as follows: 23188 for AMPEMO, 2,973 for Dean-VAE, 25,415 for Nagajaran-LSTM, 1,052 for Muller-RNN, 42,410 for PepGAN, 50,000 for HydrAMP, 20,478 for PepCVAE, 49,999 for AMP-GAN, 50,000 for FBGAN, 743 for MMCD, 527 for PrefixProt, 920 for AMP-Designer and 50,000 for MPOGAN.

Evaluation metrics. In this evaluation, we apply three predictive models to evaluate the antimicrobial activity (AMPlify (16) and AMP Scanner v2 (17)) and cytotoxicity (ToxinPred2 (1)) of AMP candidates. Specifically, we assess the probability that AMP candidates have antimicrobial activity ($P_{AMP} \in [0,1]]$) by averaging the probabilities predicted by the two antimicrobial activity predictors. These predictors were chosen considering that they showed the best performance on the same independent test dataset (see Figure 2d-h and Supplementary Table 1). A larger P_{AMP} value indicates a higher probability that an AMP candidate has antimicrobial activity. $P_{AMP} > 0.8$ is considered to have a high probability of having antimicrobial activity. Toxinpred2 is used to assess the probability that AMP candidates have peptide toxicity ($P_{Toxin} \in [0,1]$). A smaller P_{Toxin} value indicates a lower probability that an AMP candidate has peptide toxicity. $P_{Toxin} < 0.7$ is considered to have a low probability of having peptide toxicity. We expect to obtain AMP candidates that exhibit both high antimicrobial activity and low peptide toxicity, so the AMP candidates that meet both $P_{AMP} > 0.8$ and $P_{Toxin} < 0.7$ are considered high-quality AMP candidates. To evaluate sequence diversity, we calculate the minimum normalized within-group edit distance of a given set. Specifically, for each sequence in the set, we compute its Levenshtein distance to every other sequence and normalize these distances to account for length-related biases. The smallest of these normalized values is then identified. This approach measures the similarity between the most closely related sequences in the set, providing an indicator of the overall diversity within the collection. To further evaluate the diversity of the generated AMP candidates, we also calculated the Simpson diversity index and the Shannon diversity index, and performed normalization (18).

3 Baseline methods and evaluation settings for AMP identification

3.1 Baseline methods

In the AMP identification task, we compared seven baseline methods, which are MACREL (19), amPEPpy (20), Ma-NNMs (21), AMPlify (16), STM (22), AMP Scanner v2 (23), and AMP-BERT (24).

MACREL. MACREL is a pipeline for predicting AMPs from genomes and metagenomes, utilizing two feature-based classifiers to predict AMP and hemolytic activity based on 22 descriptors. The classifiers incorporate a combination of local and global features, including amino acid distribution patterns, physiochemical properties, solubility, and composition of amino acid groups. Training sets for the classifiers are carefully curated to ensure accuracy, with AMP prediction using a random forest classifier and hemolytic activity prediction based on the HemoPI-1 dataset. MACREL processes metagenomic reads, assembles contigs, predicts genes, and classifies AMP sequences based on their characteristics. The pipeline demonstrates high precision and sensitivity in identifying high-quality AMP candidates, making it a valuable tool for extracting AMPs from genomic and metagenomic data.

amPEPpy. amPEP (*25*) is a computational method that predicts AMPs using the random forest algorithm. The method utilizes distribution patterns of amino acid properties along the sequence to develop a prediction model. A large and diverse dataset of AMP and non-AMP sequences is used to evaluate different random forest classifiers with varying positive:negative data ratios. The optimal model, amPEP, achieves high accuracy, Matthew's correlation coefficient (MCC), area under the receiver operating characteristic curve (AUROC), and the Kappa statistic. Feature analysis is conducted to identify a minimal set of 23 features for AMP prediction with high accuracy. The method outperforms existing approaches in terms of accuracy, MCC, and AUROC when tested on benchmark datasets.

In our paper, we compare model performance using amPEPpy, which is the Python 3 implementation of amPEP. amPEPpy has better portability and provides a command-line user interface designed to efficiently process genome-scale data.

Ma-NNMs. Ma et al. integrate multiple natural language processing neural network models, including LSTM, Attention, and BERT, to establish a unified pipeline for identifying candidate AMPs from human gut microbiome data. By treating peptide sequences as text data and leveraging large datasets, they use these neural network models (NNMs) to predict AMPs effectively. They optimize predictive performance by combining the strengths of each model and achieve high precision and recall rates in distinguishing AMPs from non-AMPs. Additionally, they mine metagenomic and metaproteomic data to filter and select potential AMP candidates for further validation, demonstrating the power of machine learning approaches in discovering functional peptides from metagenome data.

AMPlify. AMPlify is an attentive deep learning model for the prediction of AMPs. AMPlify utilizes a bidirectional long short-term memory (Bi-LSTM) layer to encode positional information, followed by a multi-head scaled dot-product attention layer for refined sequence representation. A context attention layer generates a summary vector by learning contextual information. The model is trained on known AMPs and non-AMPs, incorporating ensemble learning to enhance performance. Additionally, attention mechanisms are applied to improve in silico AMP prediction, with a focus on discovering novel peptide-based alternatives to conventional antibiotics.

STM. The "Sense the Moment" (STM) prediction system aims to differentiate between AMPs and shuffled

versions by using hydrophobic moment values. Unlike deep learning methods that require large amounts of training data, STM focuses on the distinctive features of AMPs and shuffled peptides. By calculating the geometric average of hydrophobic moments measured on different scales, the system demonstrates high accuracy and sensitivity in predicting AMPs. This approach underscores the potential of utilizing specific physicochemical properties, such as hydrophobic moment, as effective classifiers in antimicrobial peptide prediction, presenting a promising alternative to traditional machine learning algorithms.

AMP Scanner v2. The Antimicrobial Peptide Scanner v2 (AMP Scanner v2) utilizes a deep neural network (DNN) model with convolutional and recurrent layers to identify AMPs based on primary sequence composition. The DNN model processes peptide sequences that are encoded into uniform numerical vectors, which are then fed through an embedding layer, convolutional layer, and LSTM layer to capture position-invariant patterns and automatically extract features. This model's architecture enables efficient recognition of AMPs without requiring domain experts to construct features beforehand. Moreover, the model learns a reduced alphabet representation of peptide sequences to further improve the accuracy of AMP recognition.

AMP-BERT. AMP-BERT is an advanced AMP classification model that utilizes a fine-tuned Bidirectional Encoder Representations from Transformers (BERT) architecture. Designed to extract structural and functional information from input peptide sequences, AMP-BERT distinguishes between AMPs and non-AMPs with superior accuracy. By leveraging the attention mechanism inherent in BERT, the model not only improves prediction performance but also provides interpretability by identifying specific amino acid residues that contribute to antimicrobial function. This attention-based analysis offers valuable insights into the structural properties of AMPs, facilitating the identification of candidate peptides for functional validation and drug development.

3.2 Evaluation settings

The calculation of evaluation metrics for the AMP identification methods is as follows:

$$\text{Accuracy} = \frac{TP + TN}{TP + TN + FP + FN}$$

$$\text{Sensitivity} = \frac{TP}{TP + FN}$$

$$\text{F1 score} = \frac{2 \times \text{Precision} \times \text{Recall}}{\text{Precision} + \text{Recall}}$$

$$\text{MCC} = \frac{TP \times TN - FP \times FN}{\sqrt{(TP + FP)(TP + FN)(TN + FP)(TN + FN)}}$$

where TP, TN, FP, and FN stand for true positive, true negative, false positive, and false negative, respectively. Precision is defined as $\frac{TP}{TP+FP}$, and Recall is defined as $\frac{TP}{TP+FN}$. The predictor is trained on the training set and evaluated on the validation set. The final performance is reported on the test set.

4 Robustness assessment of MPO stage

During the MPO stage, the dynamic dataset is continuously updated. At each update, the newly added

data changes the composition of the dataset before the update. We aim to avoid undesired biases and ensure that the model still learns the features of experimentally validated AMPs. As shown in Supplementary Figure 2, we evaluated the changes in the composition of the dynamic training dataset during the MPO stage. The dynamic training dataset is initialized with experimentally validated AMPs before the training starts, and the number of generated sequences and experimentally validated AMPs change rapidly in the first 4 iterations, and remain stable in the subsequent iterations, with slight fluctuations between 650-700 and 300-350, respectively. This proves that the MPO process is robust, and the dataset is able to maintain a stable compositional state while being dynamically updated.

5 Molecular dynamic simulations

5.1 Molecular dynamics simulation of unstructured coil conformation AMPs in TFE solution

To study the behavior of the generated AMPs in TFE solution, molecular dynamics (MD) simulations of the two most promising peptides, MPOP-03 and MPOP-07, were performed. The 3D structures of the peptides were predicted using AlphaFold3 (*26*), and the peptides were placed in a water box, heated to 500 K to completely disrupt their original structures, resulting in unstructured coil conformations. These unstructured conformations were then positioned at the center of a box containing 20% TFE solution, with K^+ and Cl^- ions added to maintain the system's electroneutrality. Steric clashes were eliminated through energy minimization using the steepest descent algorithm. The simulations were conducted using a time step of 2 femtoseconds, ensuring accuracy in the integration of Newton's equations of motion. This simulation was executed utilizing Gromacs 2024.2 (*27*) software. The LINCS algorithm (*28*) was used to constrain bonds involving hydrogen atoms. Electrostatic interactions were calculated using the Particle-Mesh Ewald (PME) algorithm (*29*), with a real-space cut-off for pairs more than 1.2 nm apart. Lennard-Jones interactions were gradually switched to zero between 1 and 1.2 nm using the force-switch algorithm (*30*). During the equilibration stages, pressure coupling was managed by the Berendsen barostat (*31*), which was later transitioned to the Parrinello-Rahman barostat (*32*) for all production stages. The barostat time constant and compressibility factor were uniformly set to 5 ps and 4.5×10^{-5} bar^{-1}, respectively. The v-rescale thermostat (*33*) maintained the temperature at 310.15 K. After a 200ns simulation period, the MD simulation results were then visualized using PyMOL (*34*).

5.2 Molecular dynamics simulation of AMPs in membrane-mimicking environment

To study the behavior of the generated AMPs in membrane-mimicking environment, 200 ns MD simulations were performed using the *α*-helical structures of MPOP-03 and MPOP-07 predicted by AlphaFold3 as the initial conformations. The AMPs were initially placed above the membrane with their axes parallel to the membrane surface. The CHARMM36 (*35*) force field was used, and the bilayer membrane was prepared using CHARMM-GUI (*36*), consisting of 75% DLPC and 25% DLPG with shorter tails to accelerate the simulation, alongside a smaller water box (*37*). The box dimensions were set to 98 Å, and 15% KCl was added to neutralize the system. The simulations were conducted with a time step of 2 femtoseconds. The Verlet cutoff scheme was employed, with a neighbor list update frequency of 20, a cutoff radius of 1.2 nm, and van der Waals interactions handled using the force-switch modifier between 1.0 and 1.2 nm. Electrostatic interactions were calculated using the PME method with a cutoff of 1.2 nm. The v-rescale

thermostat maintained the temperature at 310.15 K. Pressure coupling was semi-isotropic, controlled by the C-rescale barostat with a coupling constant of 5.0 ps and a compressibility of 4.5×10^{-5} bar^{-1} in both directions, maintaining a reference pressure of 1.0 bar. Hydrogen bond constraints were applied using the LINCS algorithm. The results were visualized using PyMOL.

Supplementary Tables

1 Performance evaluation of AMP prediction methods on independent test set

Supplementary Table 1 Performance evaluation of methods for predicting AMPs on the independent test set. Bolded numbers are the best performance.

Methods	Accuracy (%)	Precision (%)	F1 score (%)	Sensitivity (%)	Specificity (%)	AUC	MCC (%)
STM	57.79	56.92	60.27	64.05	51.52	0.5779	15.70
MACREL	76.83	93.39	71.37	57.75	95.91	0.8541	58.06
amPEPpy	77.06	80.84	75.56	70.93	83.19	0.8253	54.53
Ma-NNMs	78.49	**94.08**	73.87	60.80	**96.17**	0.7849	60.91
AMP-BERT	79.17	87.87	76.47	67.68	90.66	0.8801	59.94
AMPlify	80.47	83.90	79.43	75.41	85.53	0.8695	61.25
AMP Scanner v2	84.00	87.64	83.19	79.17	88.84	0.9082	68.33
LLM-AAP (Ours)	**88.87**	91.54	**88.50**	**85.66**	92.08	**0.9517**	**77.90**

2 Percentage of AMP candidates generated by (pre-trained) MPOGAN across different E-value categories

Supplementary Table 2 Percentage of AMP candidates generated by pre-trained MPOGAN (top row) and MPOGAN (bottom row) in different categories of Expect value (E-value). The E-value for the match with the highest score was considered, as obtained by performing a BLAST similarity search against the experimentally validated AMPs dataset. Each model generates 50,000 AMP candidates for validation.

E-value	<=0.0001	<=0.001	<=0.01	<=0.1	<=1	<=10	>10
Pre-trained MPOGAN	3.41	1.40	3.16	5.43	13.14	35.16	38.30
MPOGAN	2.83	3.22	6.16	11.70	25.17	36.16	14.77

3 Sequence information and physicochemical properties of synthesized peptides

Supplementary Table 3 Sequence information and physicochemical properties of synthesized peptides.

Name	Sequence	length	Charge	Isoelectric point	Aromaticity	Eisenberg hydrophobicity	Hydrophobic moment	Hydrophobic ratio	Charge density	Instability index	Aliphatic index
LL-37	LLGDFFRKSKEKIGKEFKRIVQRIKDFLRNLVPRTES	37	7	11.1514	0.1081	5.7999	0.5624	0.3514	0.0013	23.3432	89.4595
MPOP-01	DPFGIMSKLQQFIRKFYQSLKHLKT	25	5.094	10.7490	0.16	-0.0624	0.4582	0.36	0.0013	69.6404	78
MPOP-02	LHQIKSVIKTAMNVLSGLFSAIKKK	25	6.094	11.2788	0.04	0.1256	0.4815	0.48	0.0018	-0.588	124.8
MPOP-03	MSKFKHFFNAVKSIFRGLTK	20	6.094	11.7510	0.2	0.0025	0.6101	0.45	0.0021	13.085	58.5
MPOP-04	GGGGMLKYFKTAIHTIKKIGQKIVN	25	6.093	10.9063	0.08	0.1316	0.5456	0.36	0.0019	3.336	93.6
MPOP-05	VMKRIGTILSGLHSLLSKIF	20	4.094	11.5723	0.05	0.3005	0.5557	0.5	0.0014	26.94	151
MPOP-06	KLFRVVKKMFHSVFSSIHKYFR	22	7.192	11.5869	0.2273	-0.0382	0.7441	0.4545	0.0022	44.3227	75
MPOP-07	GIGKFLHSFTKFFSKIMNAIR	21	5.094	11.6768	0.1905	0.1776	0.6721	0.4762	0.0017	-2.6048	79.0476
MPOP-08	GLFDVIKKIAEMVSNGYHTVKKKF	24	4.095	10.2998	0.125	0.0638	0.5509	0.4167	0.0011	-1.35	89.1666
MPOP-09	IKGIIKTIKKMASHFLHSAS	20	5.193	11.1567	0.05	0.1435	0.5760	0.45	0.0019	5.56	107.5
MPOP-10	IGSFKHVFKRITSMAKAIR	19	6.094	12.1797	0.1053	-0.0132	0.4946	0.4737	0.0023	17.3053	87.3684
MPOP-Neg	LARGDCIMKLL	11	1.929	8.7432	0	0.1664	0.3288	0.6364	0.0008	-6.3455	150.9091

4 BLAST sequence similarity search of synthesized peptides in the UniProt database

Supplementary Table 4 BLAST sequence similarity search of synthesized peptides in the UniProt database.

Peptide	Min E-value
MPOP-01	0.059
MPOP-02	0.62
MPOP-03	0.16
MPOP-04	**3.4**
MPOP-05	**5.0**
MPOP-06	0.84
MPOP-07	**2.5**
MPOP-08	0.24
MPOP-09	**3.4**
MPOP-10	**1.1**

* E-value < 1×10^{-100}: Identical sequences.
1×10^{-100} < E-value < 1×10^{-50}: Almost identical sequences.
1×10^{-50} < E-value < 1×10^{-10}: Closely related sequences, could be a domain match or similar.
1×10^{-10} < E-value < 1: Could be a true homologue but it is a gray area.
1 < E-value < 10: Proteins are most likely not related
E-value > 10: Hits are most likely junk unless the query sequence is very short.

5 AMP candidates information after preliminary selection

Supplementary Table 5 AMP candidates information after preliminary selection.

Id	Sequence	LLM-AAP score	AMP Scanner v2 score	AMPlify score	Toxinpred2	Weighted score
**1**	DPFGIMSKLQQFIRKFYQSLKHLKT	0.9934	0.9978	0.9999	0.2510	0.7420
**2**	LHQIKSVIKTAMNVLSGLFSAIKKK	1.0000	0.9997	0.9955	0.3170	0.7205
**3**	MSKFKHFFNAVKSIFRGLTK	1.0000	1.0000	1.0000	0.4200	0.7183
**4**	GGGGMLKYFKTAIHTIKKIGQKIVN	1.0000	0.9978	0.9977	0.3260	0.7175
**5**	VMKRIGTILSGLHSLLSKIF	0.9989	1.0000	1.0000	0.4240	0.7169
**6**	KLFRVVKKMFHSVFSSIHKYFR	0.9999	1.0000	0.9999	0.3890	0.7161
**7**	GIGKFLHSFTKFFSKIMNAIR	1.0000	0.9989	1.0000	0.4240	0.7106
**8**	GLFDVIKKIAEMVSNGYHTVKKKF	1.0000	1.0000	1.0000	0.3700	0.7097
**9**	IKGIIKTIKKMASHFLHSAS	0.9997	1.0000	1.0000	0.4490	0.7086
**10**	IGSFKHVFKRITSMAKAIR	0.9997	1.0000	1.0000	0.4690	0.7082
11	GKTVSHVGNKIMSVIKHFVH	1.0000	0.9966	0.9971	0.4550	0.7060
12	TKAIQHNIMRFVSKFFQKIKKVR	0.9997	1.0000	0.9995	0.3870	0.7031
13	FLSHIKGMFSSFFGKILNRV	1.0000	1.0000	0.9998	0.4410	0.7030
14	ILGKIGSVLSTIHASVNHLMKKFRR	1.0000	0.9998	0.9998	0.3710	0.7030
15	LFNIIKRIYSTIAGLIRNIMHQPRG	0.9984	0.9999	1.0000	0.2930	0.7021
16	YHHFMRKVINAMKKIWKFIP	1.0000	1.0000	1.0000	0.4700	0.7017
17	QFLHEMNKMFRRITRWIRK	1.0000	0.9998	0.9995	0.4910	0.7008
18	KIKSQIGKASTMLKGFFSEIFRKVH	1.0000	0.9997	0.9998	0.3810	0.6996
19	ALHKIVSVAGRMLSTIFSSVKK	1.0000	0.9998	0.9996	0.3990	0.6975
20	ESWAQWVRYFMRSFRRFIRSVR	1.0000	1.0000	1.0000	0.4530	0.6948
21	ARAINRIYQGVKHIAKHIMGK	1.0000	0.9999	1.0000	0.4720	0.6947
22	KKESLIHMLKKLWQILKNYL	0.9923	0.9998	0.9986	0.4650	0.6940
23	SKKKIVEAMKKFLKKIANHVLGQIT	0.9999	1.0000	1.0000	0.3600	0.6933
24	GSWVKITSHVMQTIKKIARLWNRK	1.0000	0.9999	0.9998	0.4230	0.6920
25	WSLFSRIRELFRQIMRYLRQRG	0.9999	1.0000	0.9985	0.4380	0.6920
26	RRFKQIVRRIYSSILSTIRRLLRRM	1.0000	1.0000	0.9998	0.3840	0.6920
27	FPFTIWQNRVRTYIRKMVKKIK	1.0000	0.9995	0.9913	0.3910	0.6917
28	SFLSMIKHALNHALKTLKALGHN	0.9998	0.9997	1.0000	0.4560	0.6874
29	KKLMKKALQFGKHFLHSFKKIFRQY	1.0000	1.0000	1.0000	0.4180	0.6873
30	ATIMGTVANHLTKFLKGIMTSLKQ	1.0000	1.0000	0.9974	0.4430	0.6851
31	WARHMTRLWRYFMRWARSFLR	1.0000	0.9999	0.9914	0.4990	0.6848
32	TIKQFIKKIGKALGSLMKIASHFVP	1.0000	1.0000	0.9909	0.4240	0.6843
33	FVSQMKGLLKSALKTIAGHLAK	1.0000	1.0000	0.9999	0.4880	0.6831
34	GIHQIWHKMKTYLKKFTKHIKGVKK	1.0000	0.9999	0.9996	0.3520	0.6826
35	LFRLISRMTRLAKGMFSAFSSIF	0.9971	1.0000	1.0000	0.4740	0.6811
36	FWGRYFSGIMHRVRSIVRTIVRRVV	1.0000	0.9984	0.9993	0.4360	0.6811
37	SSSMLKHLKSVFKKIAQWLKEIIKK	1.0000	0.9996	0.9979	0.4360	0.6811
38	GIFSSIKSLGKKVVKGVAHHLM	1.0000	1.0000	1.0000	0.4950	0.6808
39	FPMHVYSHRIWTAIKHFGKFRRAWV	0.9974	0.9994	1.0000	0.4000	0.6796
40	SLSKVLSRAKKFLSSIFNVMKNK	1.0000	1.0000	1.0000	0.4800	0.6794

41	KKLFKKFLHFGTGALHAASNIM	0.9999	1.0000	0.9999	0.4990	0.6794
42	LLHKLGSVLKAGMNSLLKGISSK	1.0000	0.9981	0.9997	0.4800	0.6792
43	GMFGTFKGLVEAIKSAIRGYKKVI	0.9997	1.0000	1.0000	0.4620	0.6790
44	FFPIIGKILSTMLSSFHKLFKKAKQ	1.0000	0.9997	0.9972	0.3620	0.6790
45	RLAHVIKHLLSSAVRYLTSMFCKIT	0.9909	0.9997	0.9997	0.3610	0.6786
46	MLPSYLKIARNIARAIHRKIQNQR	0.9922	0.9997	0.9982	0.4620	0.6780
47	GTLIYKLMSRGKKLFKGISKF	0.9998	0.9993	0.9961	0.4970	0.6779
48	MGKAASTVVSTVAKVASKVINKL	1.0000	1.0000	0.9998	0.4860	0.6774
49	GIGKFLHSAKKFAKKILNSIMQHF	1.0000	1.0000	0.9998	0.4690	0.6767
50	DWVSLRSRIYNVFRMFRRWRR	1.0000	0.9993	0.9928	0.5000	0.6766
51	RLGKIFKRAMKIGSHIISTVHKHVN	1.0000	1.0000	1.0000	0.4520	0.6760
52	GIGKAHTLWSKAVQMIKSIGKRV	0.9998	1.0000	1.0000	0.4940	0.6747
53	FLSHIMGKLGNIFRGAIKTIIHHVN	1.0000	0.9981	0.9983	0.4560	0.6743
54	RLKKIFKKIMSAVRRMYSSIMHHFK	1.0000	1.0000	1.0000	0.3990	0.6737
55	GLFSIISKIVSHLGHFAKKAMNKIS	1.0000	1.0000	0.9997	0.4190	0.6736
56	GNLRGILSKFEHVFRGVSKIMSKVF	1.0000	0.9995	0.9980	0.4620	0.6724
57	IWKHIMKSIIKTVTHFFNTFKKW	1.0000	0.9994	1.0000	0.4580	0.6722
58	TLKKILKKILHHLIKSIHKMF	1.0000	0.9975	0.9922	0.4900	0.6717
59	MNWQRVMKTFQKFWKYLNKYKW	1.0000	0.9999	0.9995	0.4770	0.6715
60	GVSKLKSVSKTVMHLWKHALNKFK	1.0000	1.0000	1.0000	0.4870	0.6707
61	KILKRIATIFSHIKNFFRWIKNMRI	1.0000	1.0000	0.9962	0.4690	0.6699
62	GLPPLKSVFRGIKGVASHFFNSIM	0.9966	1.0000	0.9999	0.4260	0.6698
63	RIGSFLSSAMGTVVGAISTIIKKLL	1.0000	0.9998	0.9973	0.4720	0.6690
64	GLFDVIKKIGSILHSFGQLAKNIKM	1.0000	1.0000	0.9976	0.4730	0.6687
65	GIGKFFHAGKSMGKKILSTITHLK	1.0000	0.9973	0.9928	0.4920	0.6679
66	IKGIIKKFAKIISHIASHIMNHIHH	1.0000	1.0000	0.9995	0.4820	0.6659
67	TLTQWMNKLLKKFLKKIGHIWKIL	0.9999	0.9963	0.9977	0.4580	0.6658
68	ITWKKYVQGAIQSLKKFAKKFM	1.0000	1.0000	0.9999	0.4770	0.6640
69	KRVVRKALKGITHTFTSMLRSLSKK	1.0000	1.0000	1.0000	0.4880	0.6640
70	GLLDVLKNVVSHVAETIMNSIKEK	1.0000	0.9999	1.0000	0.4240	0.6639
71	GLFDVIKKWVHSGYRGWYRPMYRQR	0.9998	1.0000	0.9992	0.4280	0.6639
72	NMIKGIIHHAGKLFRSIVKDLSKLF	1.0000	1.0000	1.0000	0.4940	0.6620
73	GIGKFLHSAKKFFKGIAEGLKNIMR	1.0000	1.0000	0.9950	0.4930	0.6618
74	KKIMQQIGKYFGTFMKTFKKIAKLK	1.0000	0.9995	0.9994	0.4970	0.6609
75	IGVKFTKSFFRMIRTIYRRLKK	1.0000	0.9998	1.0000	0.4870	0.6607
76	GWMRSIWSFIKSAAQHFFKAIHATH	1.0000	0.9978	0.9951	0.4560	0.6605
77	IKCFHKMFERVRRIVHGVRQAIKRW	1.0000	1.0000	1.0000	0.4990	0.6603
78	KVFRRVYSGVLRYAATMYKNLGKPI	0.9934	0.9999	1.0000	0.4970	0.6603
79	INWKKIVETGMQFLKNFVKKIVNN	0.9999	1.0000	1.0000	0.4980	0.6601
80	HGLANFMKAAKKALNHIANQVSSLF	1.0000	1.0000	1.0000	0.5000	0.6600
81	ALGSIWSMAKHIAKSVAKKVTNHF	1.0000	1.0000	0.9999	0.5000	0.6594
82	AKHNFGRLFRTFMTLGKKFTNKIKK	1.0000	1.0000	1.0000	0.4820	0.6593
83	VMKSLASILRTVGSLGKTVHMGINF	0.9962	0.9999	0.9998	0.4220	0.6589
84	FFGRIPKMFSAISHLAKKIASKFQR	1.0000	1.0000	0.9996	0.4440	0.6586
85	LSFLPMMKSVLSSVLKKVFNAIKNH	0.9999	1.0000	1.0000	0.4090	0.6570
86	AKKMFKKVFKSYTKSFLGLLSNAGN	0.9992	0.9998	0.9996	0.4710	0.6562

87	GLFRRIKSIFKSVFKTVAHHAMKKV	1.0000	1.0000	1.0000	0.4920	0.6560
88	KLHKYLGHIWKFIKKFFKGGGMQVA	1.0000	0.9978	0.9942	0.4160	0.6538
89	SFLGMIGKLGKHVLRHVSSVFPSMI	0.9999	0.9993	0.9960	0.4640	0.6515
90	FFKHIFRNIGSMASKIPSAFCKIFR	0.9995	1.0000	0.9945	0.4270	0.6503
91	VHNVMKHVKQAVKHVLCTVSAYGYG	1.0000	1.0000	0.9996	0.4490	0.6503
92	KKLFKKIMSSFFHKFPHFICSLTKK	0.9957	0.9984	1.0000	0.4090	0.6497
93	GIWSKIVGAIKHVMKTVAPHFHS	1.0000	1.0000	0.9996	0.4830	0.6494
94	FLSHIKSMWSKIKGLISAIHRLGAG	0.9999	0.9999	1.0000	0.4960	0.6480
95	IYQTQFVGKLAGKWTTMAKKIKNLF	0.9999	0.9999	0.9998	0.4780	0.6473
96	GLRKSIFSKWVRAFTRMIRYVRKCW	1.0000	0.9999	1.0000	0.4990	0.6470
97	KFFRRVVSSFLRYIHRLVYRMFCWI	0.9999	0.9995	0.9982	0.4210	0.6461
98	LIKWFKMFKKFFHKISSLFRGITGS	1.0000	1.0000	1.0000	0.4710	0.6430
99	LLKIISTIMKHLVSHVKQIIKKIW	0.9999	1.0000	1.0000	0.4890	0.6423
100	KRSVLRPVMRVARHVLNTAKSTYNH	1.0000	1.0000	0.9998	0.4740	0.6420
101	KKMFKKILSSFKHSFKKFVRGIVHR	1.0000	0.9999	0.9992	0.4550	0.6416
102	DTLMYLLKRVAKLGKNLISSVFDHI	0.9982	0.9996	0.9907	0.4550	0.6404
103	IKIRVRIWKIIKRISHFLHNAQRMP	0.9973	0.9999	0.9999	0.4580	0.6403
104	GLFRSVLGAGKKIGKVGHSHVSSMK	1.0000	0.9925	0.9907	0.4740	0.6401
105	MHQVKHVFKTIGKVLSGIFPAIHGL	1.0000	0.9998	0.9995	0.4610	0.6396
106	FFSAIKRMVSGIFSAFKHFFKKIMH	1.0000	0.9999	0.9990	0.4820	0.6392
107	ILGPVLKHIASTAESLANHIYNTIF	0.9987	0.9973	0.9977	0.4810	0.6390
108	LRAKPRIFGYVKHFLRNVVGGLFRM	0.9999	1.0000	0.9921	0.4440	0.6378
109	FLKMIGKILGSLFRTVSSLFRGIVH	1.0000	1.0000	1.0000	0.4700	0.6367
110	STLKLWHKMFKKAKTVIKSIVSGLT	1.0000	1.0000	1.0000	0.4920	0.6360
111	FFGSLLRGIATTIIKAIHTLMSKIR	1.0000	1.0000	0.9959	0.4730	0.6352
112	LFYKFLQLLKRLIKRILHTFRQSVR	1.0000	1.0000	1.0000	0.4980	0.6340
113	KFVKKIFKKIFQTFHHFFDRFGHFM	1.0000	0.9999	1.0000	0.4980	0.6340
114	FLSMIGKVFNHALSHVLGKIFKKIH	1.0000	1.0000	0.9995	0.4980	0.6339
115	FHALKFIKRFFKNFMHKIPKMHEFK	1.0000	0.9997	0.9999	0.4810	0.6330
116	KPGVVGSMAKYFKIIKKIIKRIYRA	0.9994	1.0000	1.0000	0.4860	0.6313
117	GWLNWIKTVAKHFGKSAFGKIMNSI	1.0000	0.9997	0.9994	0.4910	0.6296
118	PGVFTWFKFLHIAKGISRGIRTVMT	0.9993	0.9993	0.9998	0.4740	0.6285
119	TWSHFFQRIRQVVRRATKRFMNRVL	1.0000	0.9999	1.0000	0.4760	0.6280
120	KFKKFFKKIFKYATRGITTYGYRMR	1.0000	1.0000	1.0000	0.4780	0.6273
121	VKRFIKFFKKFFRAIHHLFHRYMRR	1.0000	0.9999	0.9939	0.4980	0.6266
122	WGLMRILTKIVQFHKKFLKWIPALK	1.0000	0.9998	0.9995	0.4910	0.6229
123	DWPRFKKFMRWFHSFLRTFFRVIHT	1.0000	1.0000	0.9998	0.5000	0.6200
124	KRMKQFWKSFGAKFFHWFGKLLSRS	1.0000	0.9981	0.9958	0.5000	0.6193

Supplementary Figures

1 Amino acid composition and length distribution of the AMP and non-AMP dataset

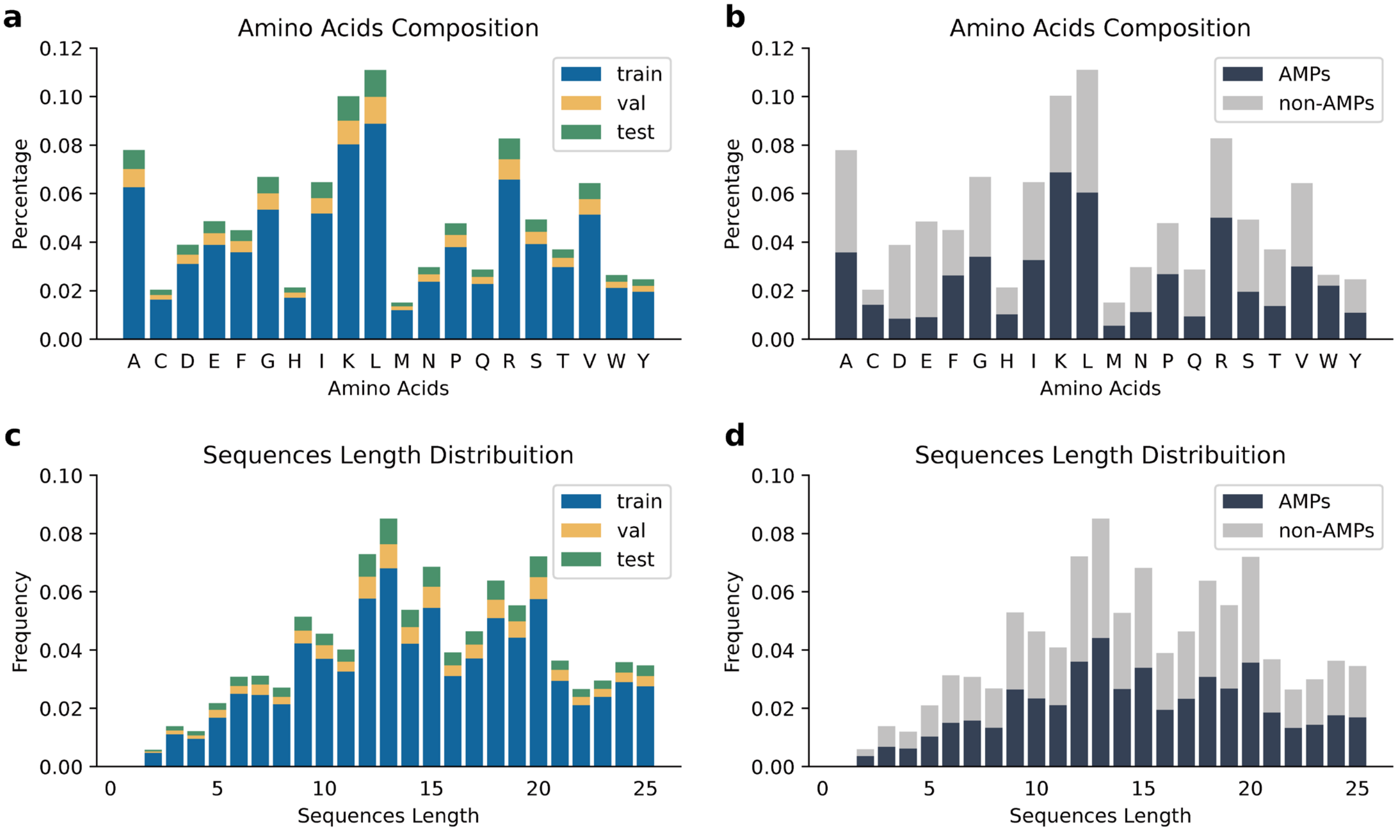

Supplementary Figure 1 Amino acid composition and length distribution of the AMP and non-AMP dataset. **a-b** Distribution of amino acid composition divided by **a** training/validation/test set and **b** AMP/non-AMP. **c-d** Sequence length distribution divided by **c** training/validation/test set and by **d** AMP/non-AMP.

2 The real-time knowledge-updating strategy for the dynamic dataset

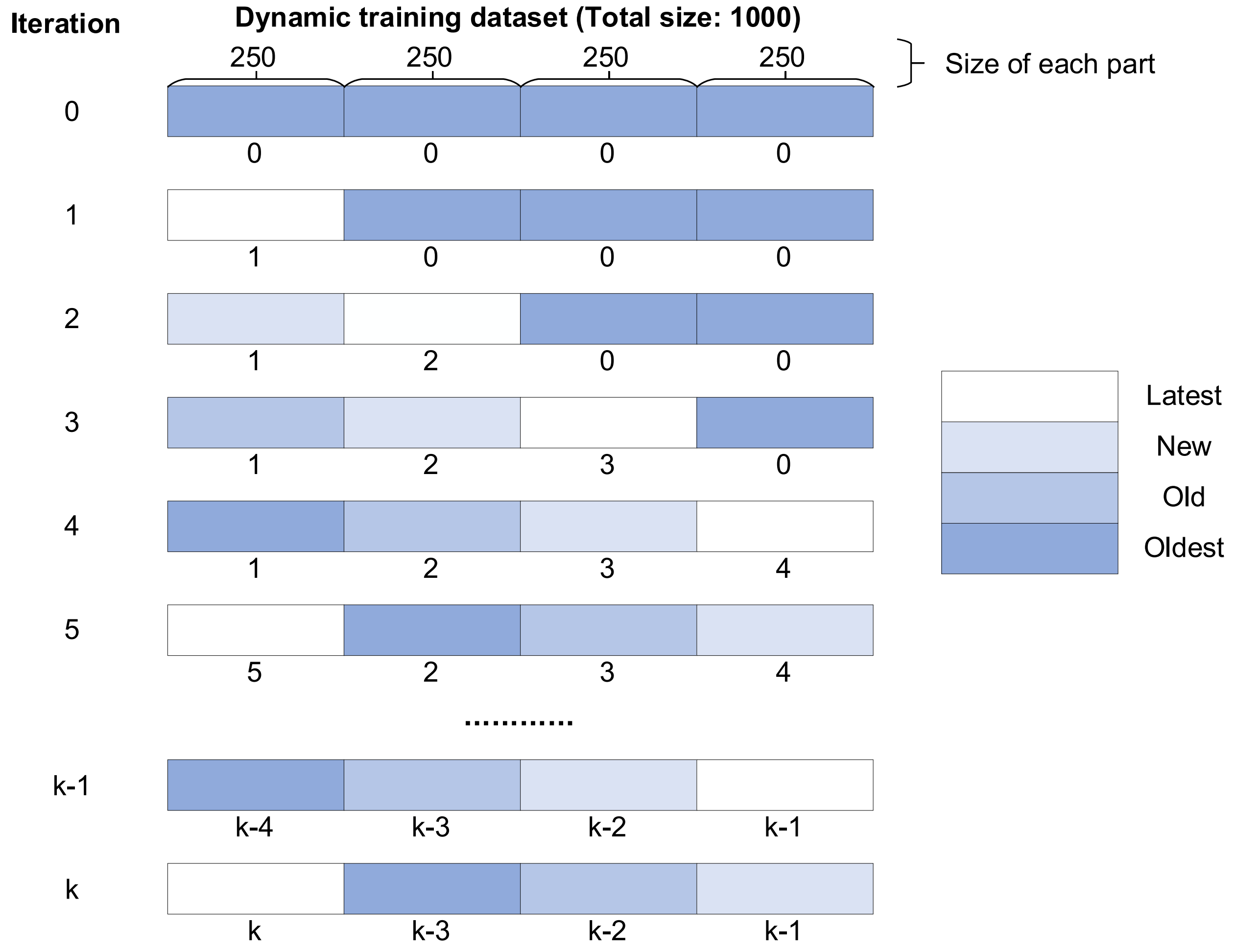

Supplementary Figure 2 The real-time knowledge-updating strategy for the dynamic dataset. Each row shows the state of the dataset after each iteration of updates. The number below each set of data represents the iteration in which the set was updated.

3 Training loss curve of MPOGAN (Pre-training, adversarial learning, and adversarial fine-tuning)

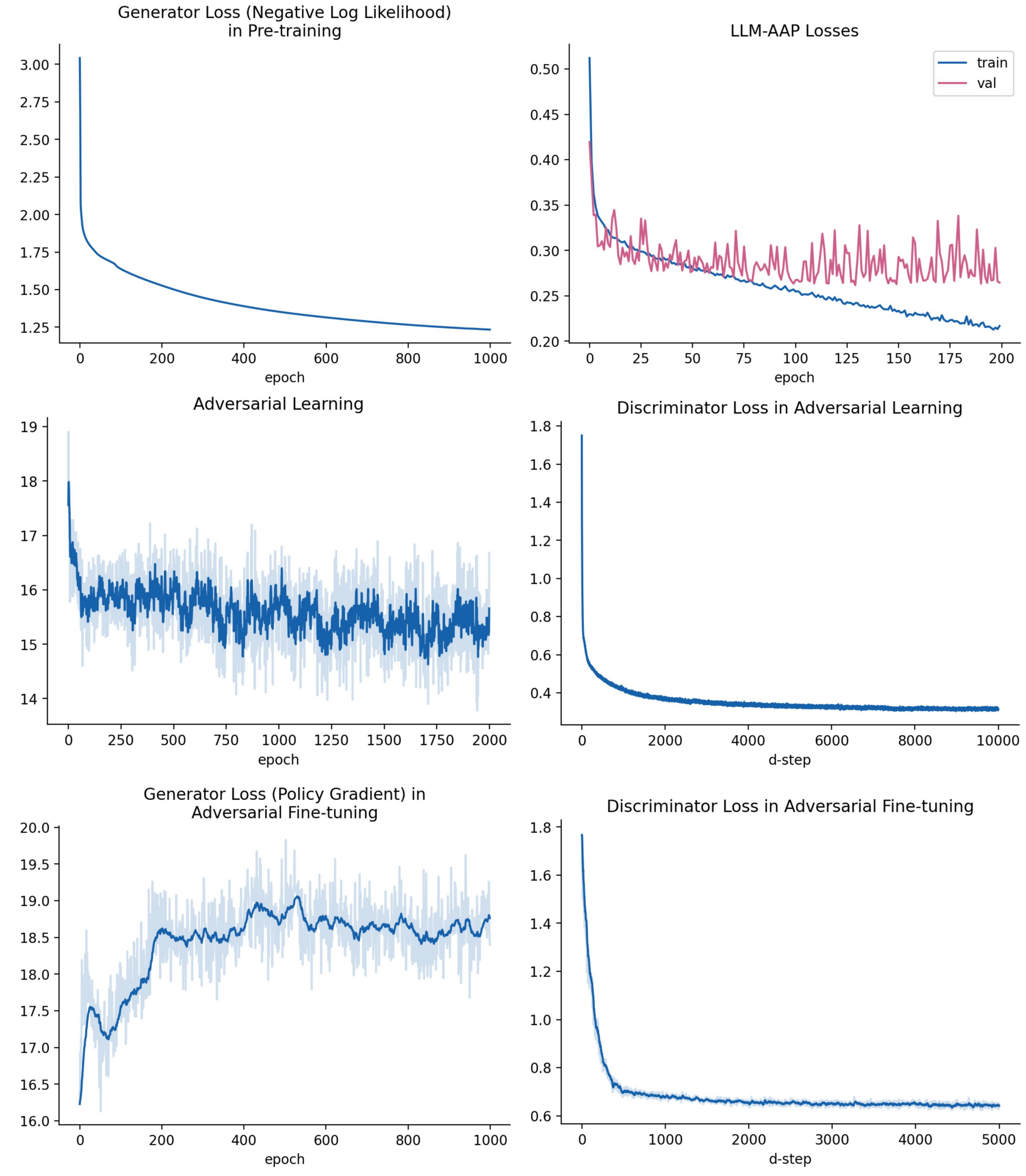

Supplementary Figure 3 Training loss curve of MPOGAN (Pre-training, adversarial learning, and adversarial fine-tuning). The ratio of training steps between generator and 138 discriminator was set to 1:5.

4 Changes in the composition of the dynamic training dataset during the MPO stage

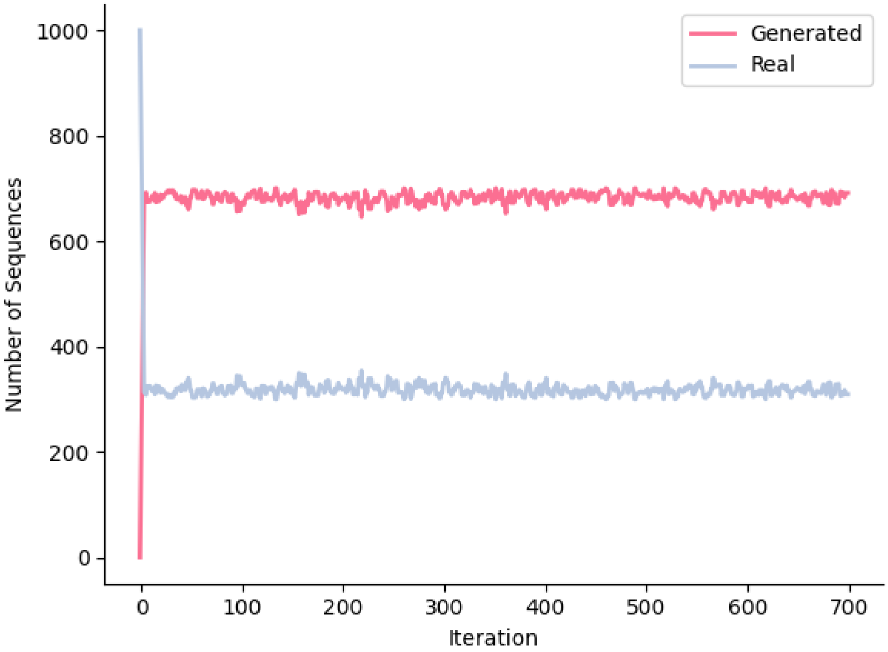

Supplementary Figure 4 Changes in the composition of the dynamic training dataset during the MPO stage.

5 Changes in the number of successfully filtered sequences in the model-embedded screening pipeline

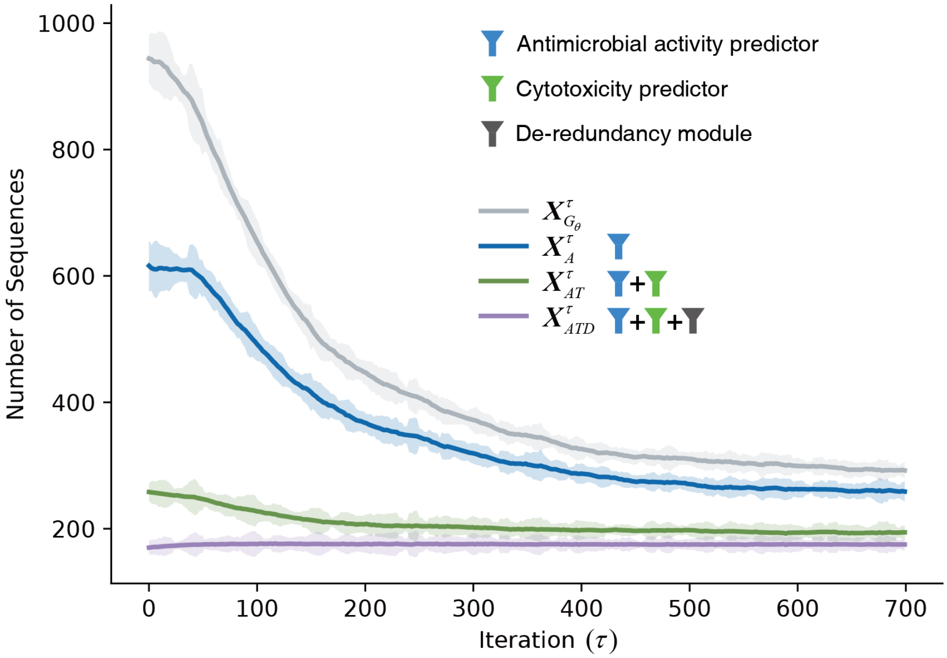

Supplementary Figure 5 Variation with iteration (τ) in the size of the AMP candidate set generated by the MPOGAN-generator ($\boldsymbol{X}^{\tau}_{G_\theta}$), along with the size of AMP candidate sets that pass through each step of the model-embedded evaluators in the screening process ($\boldsymbol{X}^{\tau}_{A}$, $\boldsymbol{X}^{\tau}_{AT}$ and $\boldsymbol{X}^{\tau}_{ATD}$). The translucent area around the curve represents the range of data fluctuations.

6 Changes in diversity and novelty of the MPOGAN-generated sequences during the MPO stage

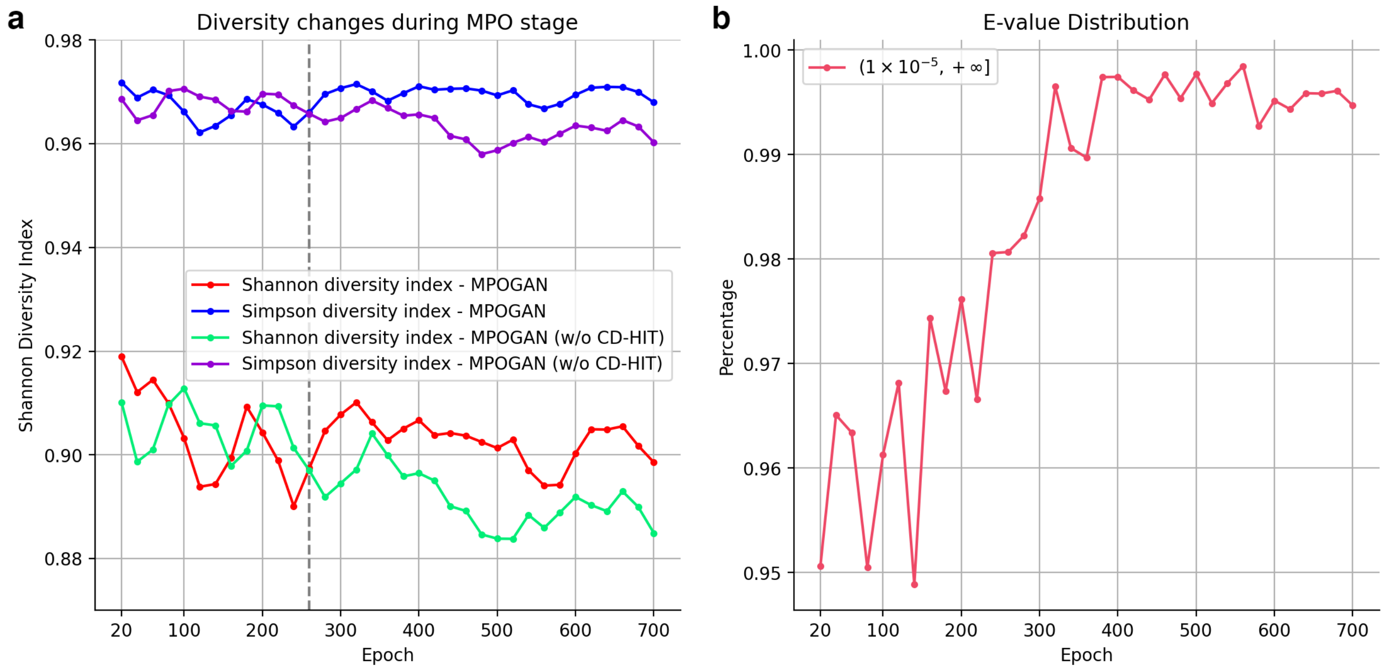

Supplementary Figure 6 Changes in **a** diversity and **b** novelty of the MPOGAN-generated AMP candidates during the MPO stage. Generator was saved every 20 training epochs, and 1,000 AMP candidates were generated at each checkpoint. Diversity was measured using the Simpson and Shannon indices, while novelty was assessed via BLAST E-values against experimentally validated AMPs.

7 Supplementary comparative evaluation of MPOGAN and generative baseline methods on generating AMPs de novo

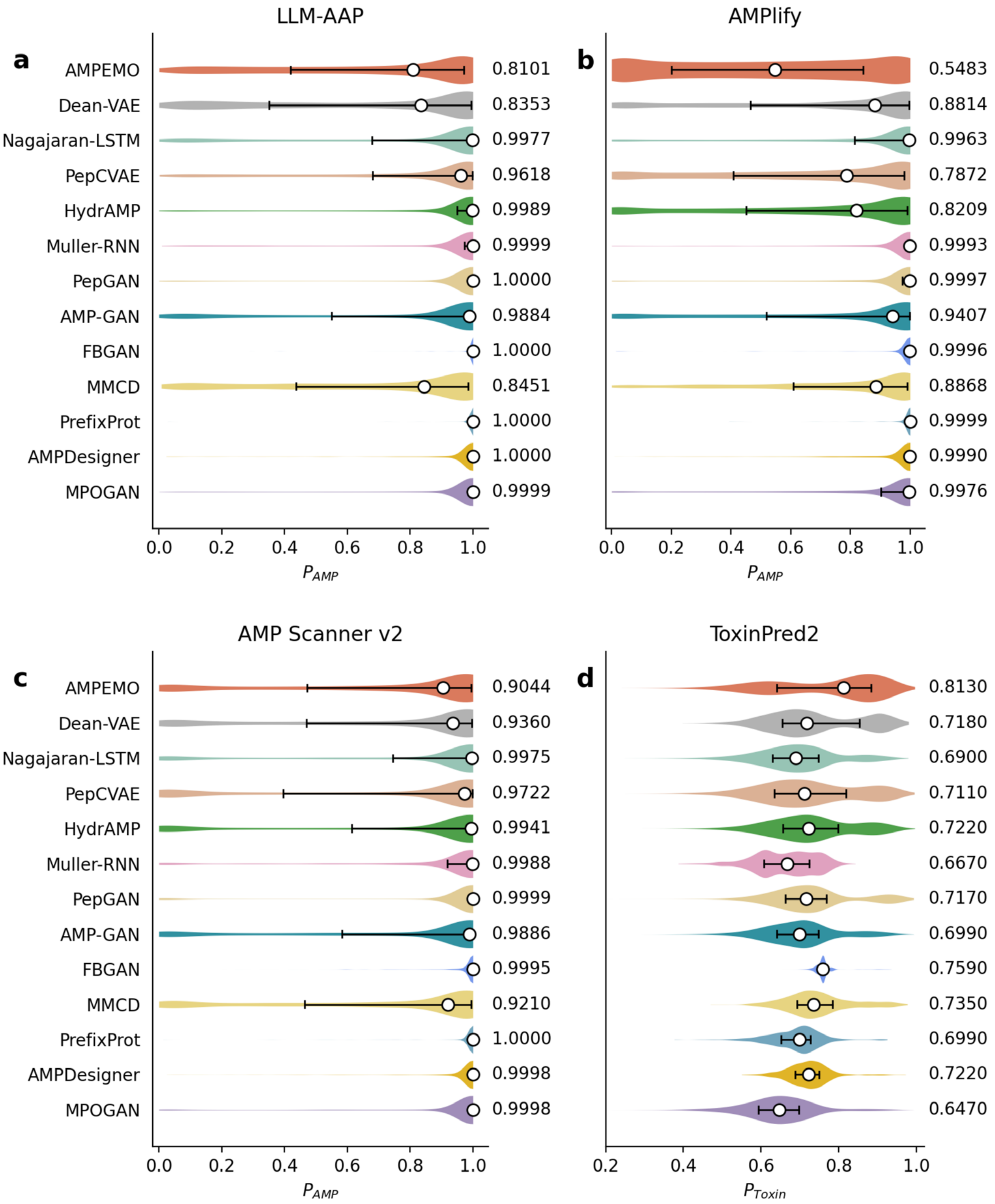

Supplementary Figure 7 Supplementary comparative evaluation of MPOGAN and generative baseline methods on generating AMPs de novo. **a-c** Probability distribution of antimicrobial activity (evaluated by **a** LLM-AAP, **b** AMPlify, **c** AMP Scanner v2 respectively). **d** Probability distribution of cytotoxicity (evaluated by ToxinPred2). The white dots indicate the median of each distribution, and the black vertical lines denote the interquartile range of each distribution.

8 Supplementary comparative evaluation of MPOGAN and generative baseline methods on the comprehensive properties of generated AMPs

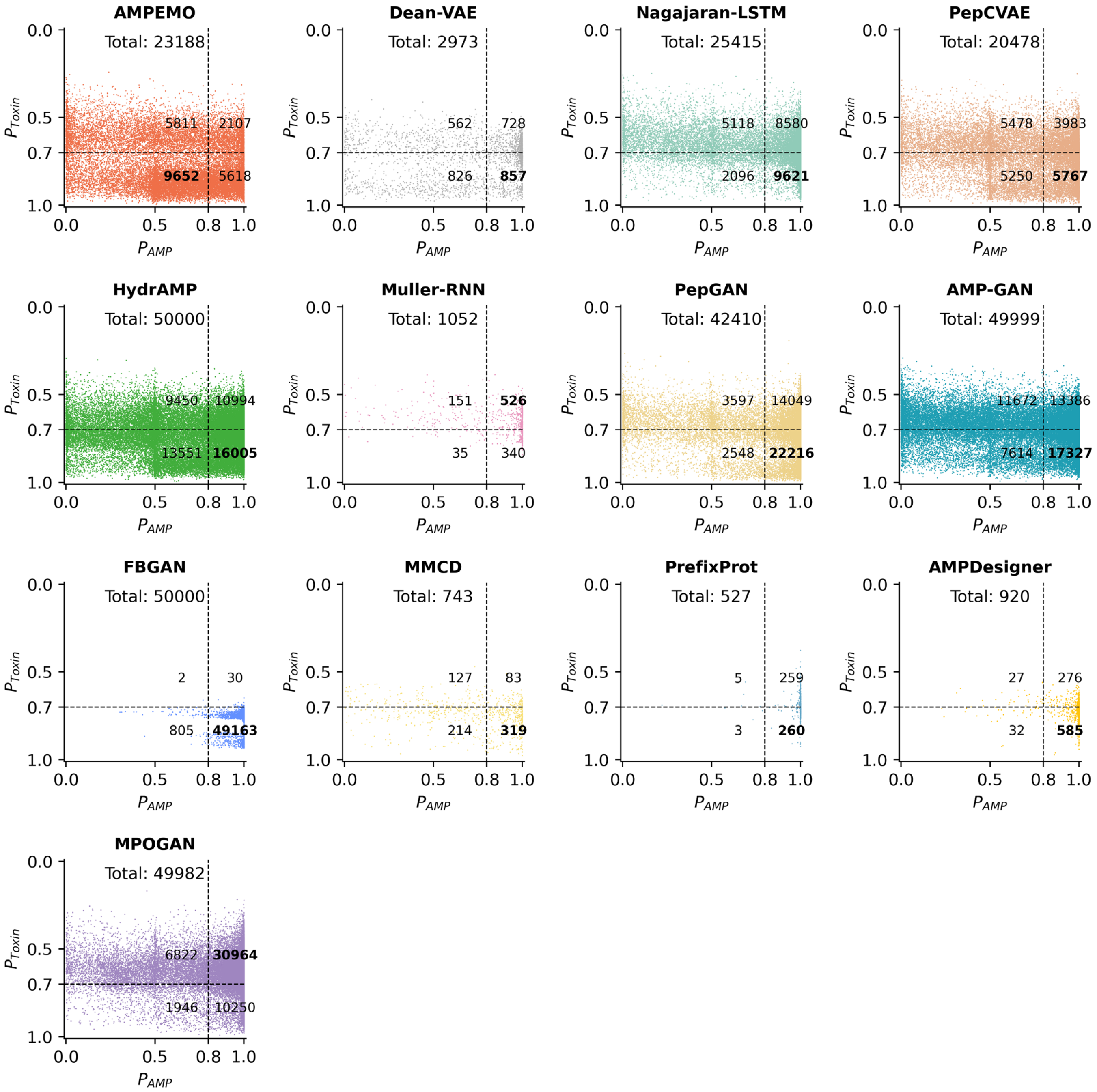

Supplementary Figure 8 Antimicrobial activity-cytotoxicity dot plots of the candidates. In each subfigure, the black horizontal dashed line indicates the cytotoxicity threshold, with low toxicity above and high toxicity below. The black vertical dashed line indicates the antimicrobial activity threshold, with high activity on the right and low activity on the left. The numbers represent the number of (P_{AMP}, P_{Toxin}) points in each area, with bold numbers highlighting the region containing the most points for a model. P_{AMP} represents the

average score predicted by two third-party antimicrobial activity predictors, AMPlify and AMP Scanner v2.

9 Analysis of plug-and-play extensibility of MPOGAN in hemolysis optimization

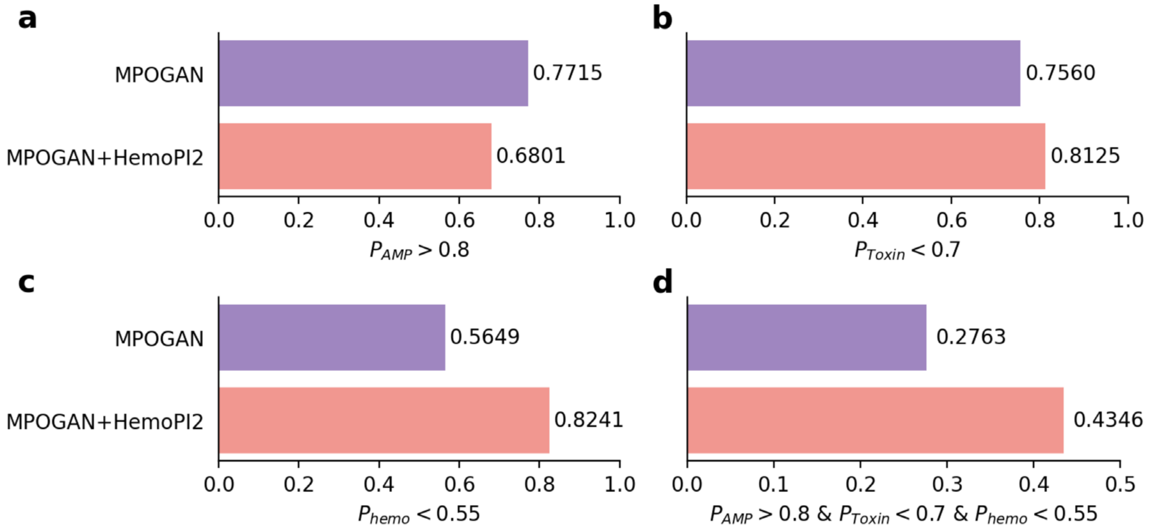

Supplementary Figure 9 The analysis of plug-and-play extensibility of MPOGAN in hemolysis optimization by comparing the original MPOGAN with its variant, MPOGAN+HemoPI2, which integrates a hemolysis evaluator (HemoPI2 (*38*)) into the model-embedded screening pipeline. This evaluation focuses on the proportion of generated sequences that exhibit **a** high antimicrobial activity, **b** low cytotoxicity, **c** low hemolytic activity, and **d** the ability to simultaneously meet all three criteria.

10 Performance comparison of MPOGAN and LLM-AAP after training on the improved dataset

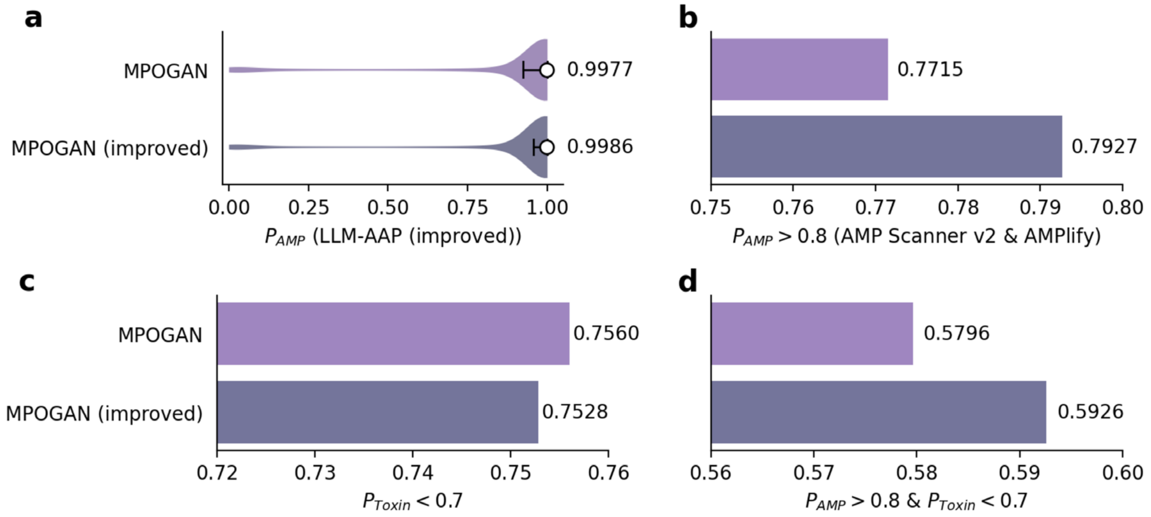

e

Methods	Accuracy (%)	Precision (%)	F1 score (%)	Sensitivity (%)	Specificity (%)	AUC	MCC (%)
STM	54.89	21.13	32.63	71.60	51.88	0.6174	16.89
MACREL	89.01	60.27	69.54	82.17	90.25	0.9290	64.15
amPEPpy	77.36	39.65	55.52	92.60	74.62	0.9169	50.47
Ma-NNMs	82.95	46.93	61.65	89.83	81.71	0.9201	56.57
AMP-BERT	83.71	48.09	61.41	84.94	83.49	0.9123	55.46
AMPlify	81.90	45.51	61.39	94.32	79.66	0.9379	57.21
AMP Scanner v2	83.45	47.87	63.62	94.85	81.40	0.9471	59.70
LLM-AAP	79.68	42.75	59.49	**97.75**	76.43	0.9493	55.97
LLM-AAP (improved)	**91.27**	**65.76**	**75.74**	89.30	**91.63**	**0.9544**	**71.80**

Supplementary Figure 10 Performance comparison of MPOGAN and LLM-AAP after training on the improved dataset. The training dataset for LLM-AAP (improved) and MPOGAN (improved) was constructed by re-evaluating positive samples from our experimentally validated AMP dataset using two third-party predictors, AMPlify and AMPScanner v2. Sequences with antimicrobial activity probabilities ⩾0.5 from both models (10,616 sequences) were retained as positive samples, while the remaining sequences (4,790) were reassigned to the negative dataset. The final training dataset comprised 8,492 positive samples and 16,156 negative samples. **a-d** Analysis of the antimicrobial activity distribution of the MPOGAN (improved) compared to **a** the original MPOGAN (using LLM-AAP (improved)), **b** the proportion of sequences with high antimicrobial activity, **c** the proportion with low cytotoxicity, and **d** the proportion simultaneously meeting both high antimicrobial activity and low cytotoxicity criteria. **e** Performance evaluation of methods for predicting AMPs on the GRAMPA dataset. Bolded numbers are the best performance. For the GRAMPA dataset, we utilized the GRAMPA database (39), selecting sequences with MIC ⩽32 μg/ml as positive samples and those with MIC >32 μg/ml as negative samples. Additional negative samples were sourced from the UniProt dataset. After ensuring no overlap with the training data, the test dataset consisted of 757 positive samples and 4,204 negative samples.

11 Confusion matrix of methods for AMPs prediction on the independent test set

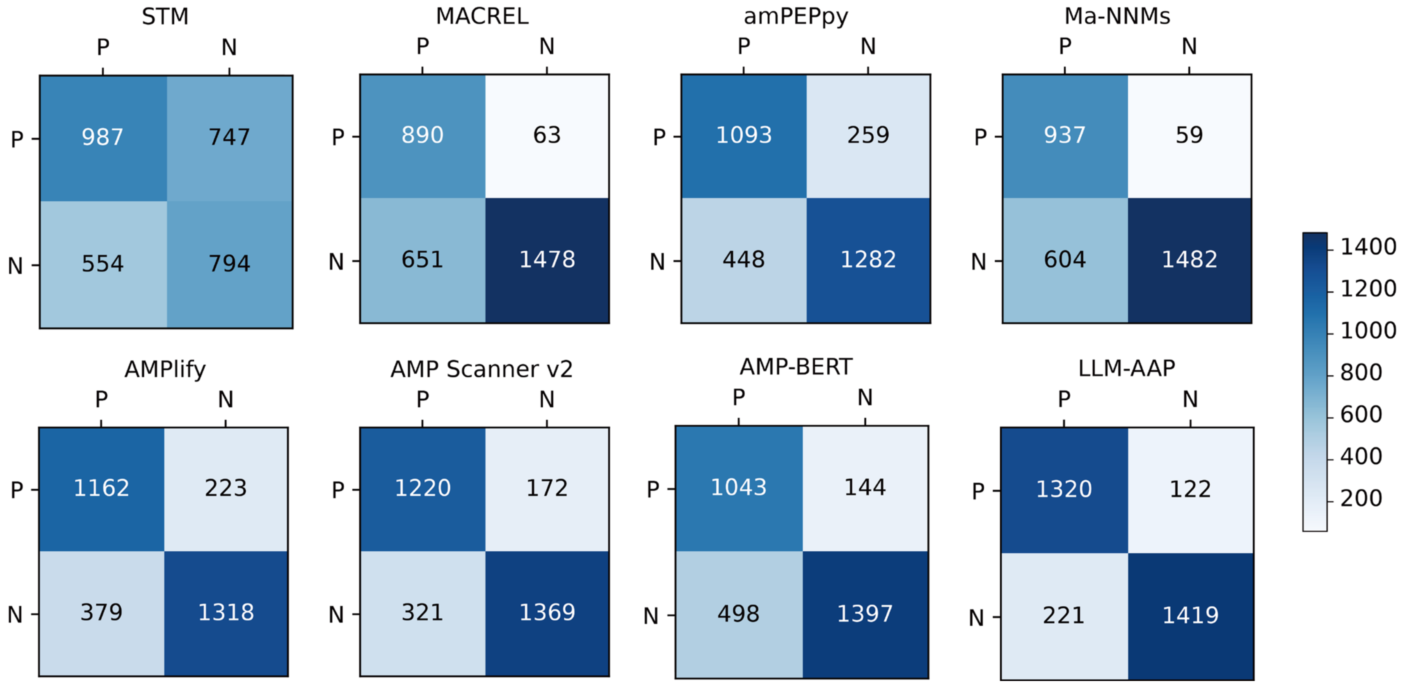

Supplementary Figure 11 Confusion matrix of methods for AMPs prediction on the independent test set. The x-axis represents the true label, and the y-axis represents the predicted label.

12 Visualization of experimentally validated AMPs and generated peptides from various training stages

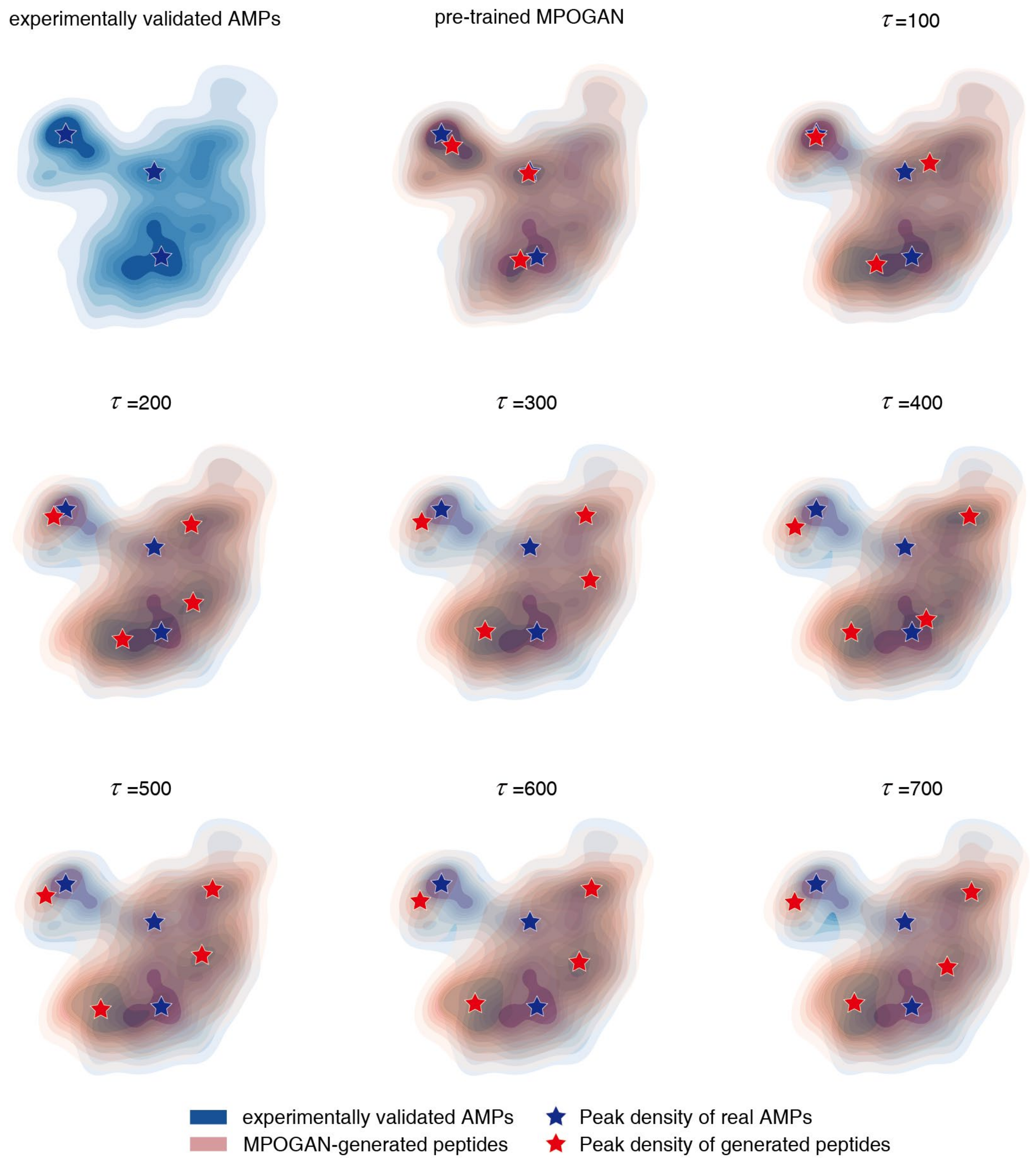

Supplementary Figure 12 Visualization of experimentally validated AMPs and generated peptides from various training stages using Uniform Manifold Approximation and Projection (UMAP) kernel density plot. The data comprises the experimentally validated AMPs dataset (15406 sequences), peptides generated by the pre-trained MPOGAN (5000 sequences), and peptides generated at various iterations (τ) in the MPO stage (100, 200, 300, 400, 500, 600, and 700 epochs, each with 5000 sequences). Nine physicochemical

properties (charge, isoelectric point (pI), aromaticity, Eisenberg hydrophobicity, hydrophobic moment, hydrophobic ratio, charge density, instability index, and aliphatic index) are calculated for all sequences using modlAMP and then standardized. Subsequently, these properties are reduced to a common two-dimensional space. In the visualization, the AMPs dataset is represented in blue, while peptides generated at different stages are in red, with darker regions indicating higher data density. Peaks of data density for the AMPs dataset and generated peptides are denoted by blue and red pentagrams, respectively. It is observed that throughout the training process, the generated peptides exhibit a similar data manifold to the experimentally validated AMPs. Moreover, the pre-trained MPOGAN effectively captures the density distribution of the AMPs dataset, demonstrating the effectiveness of the pre-training stage. During the MPO stage, the peak of data density for generated sequences gradually shifts, stabilizing after ~500 iterations, indicating the convergence of the MPO process. In conclusion, MPO-GAN is able to generate peptides resembling the true AMPs distribution but exhibiting preference differences.

13 Comparison of 5 physicochemical properties between MPOGAN generated sequences (MPOPs-50k, MPOPs-124) and experimentally validated AMPs

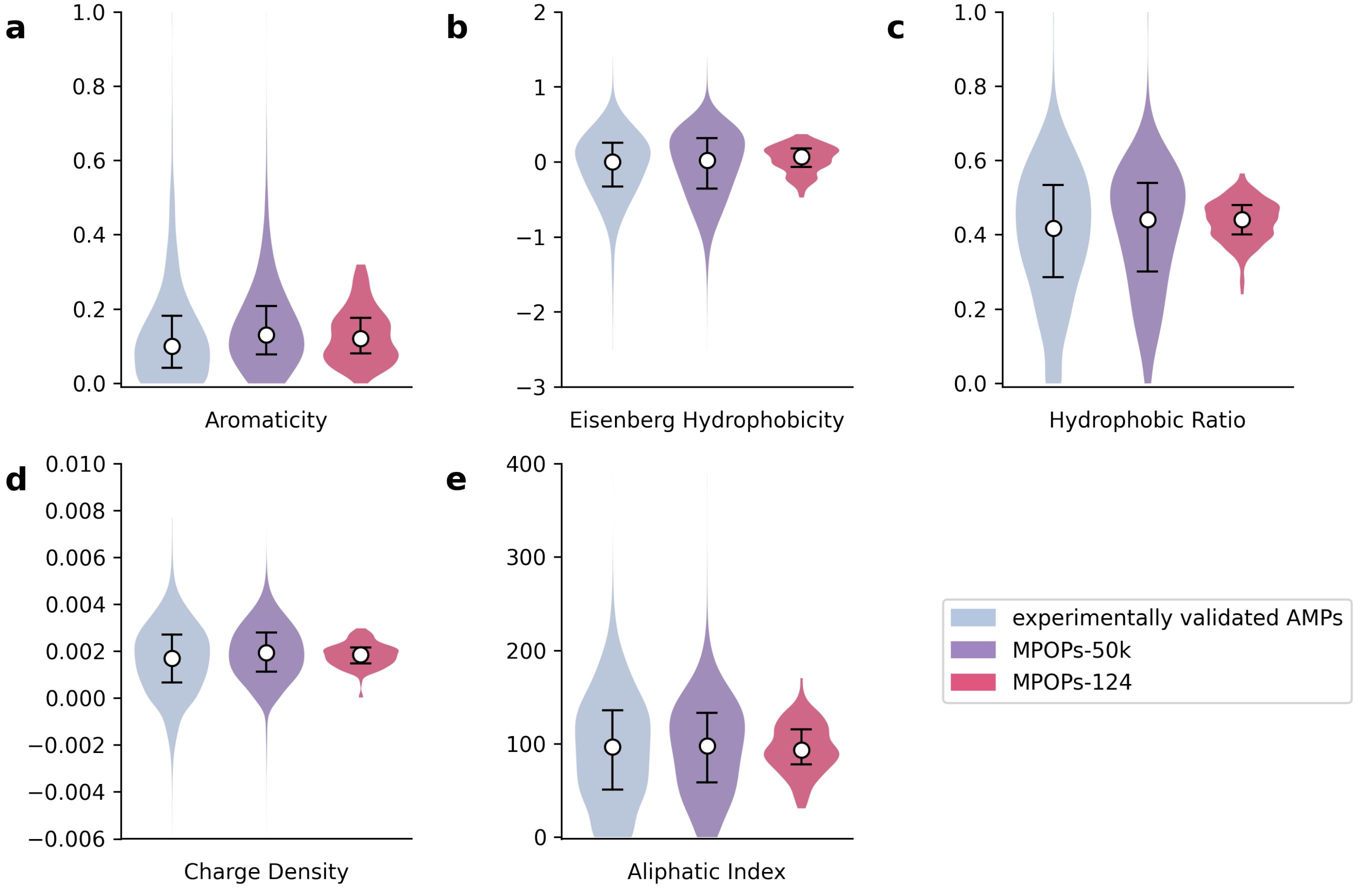

Supplementary Figure 13 Comparison of 5 physicochemical properties between MPOGAN generated sequences (MPOPs-50k, MPOPs-124) and experimentally validated AMPs. The white dots indicate the median of each distribution, and the black vertical lines denote the interquartile range of each distribution.

14 Supplementary analysis of the diversity and novelty of MPOGAN-generated sequences

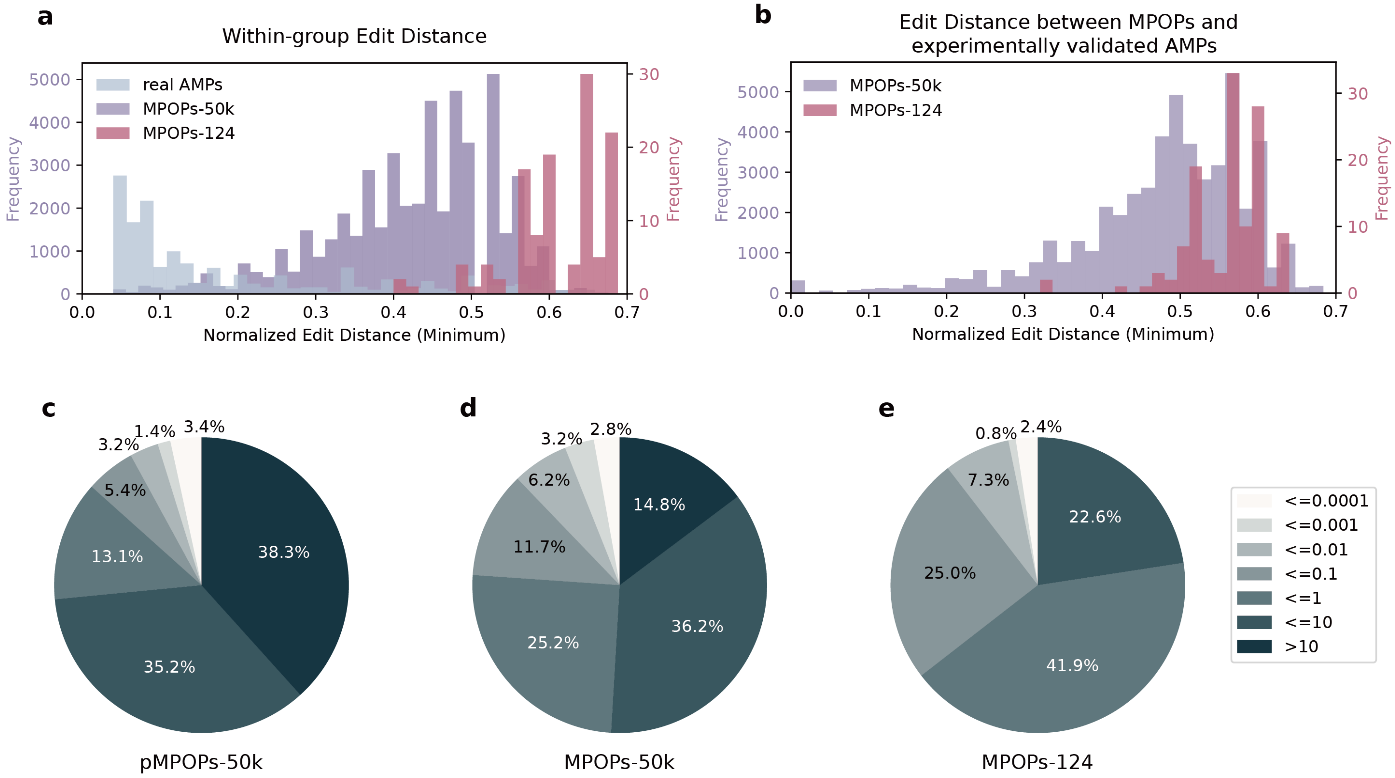

Supplementary Figure 14 Supplementary analysis of the diversity and novelty of MPOGAN-generated AMP candidates. **a-b** The minimum normalized edit distance for experimentally validated AMPs, MPOPs-50k and MPOPs-124: **a** within-group edit distance (relative to themselves) and **b** inter-group edit distance (relative to experimentally validated AMPs). **c-e** The Expect value (E-value) distribution of pMPOPs-50k (50,000 AMP candidates that are directly generated by **c** pre-trained MPOGAN), **d** MPOPs-50k, and **e** MPOPs-124.

15 Violin plots of match score distributions from peptide group comparisons

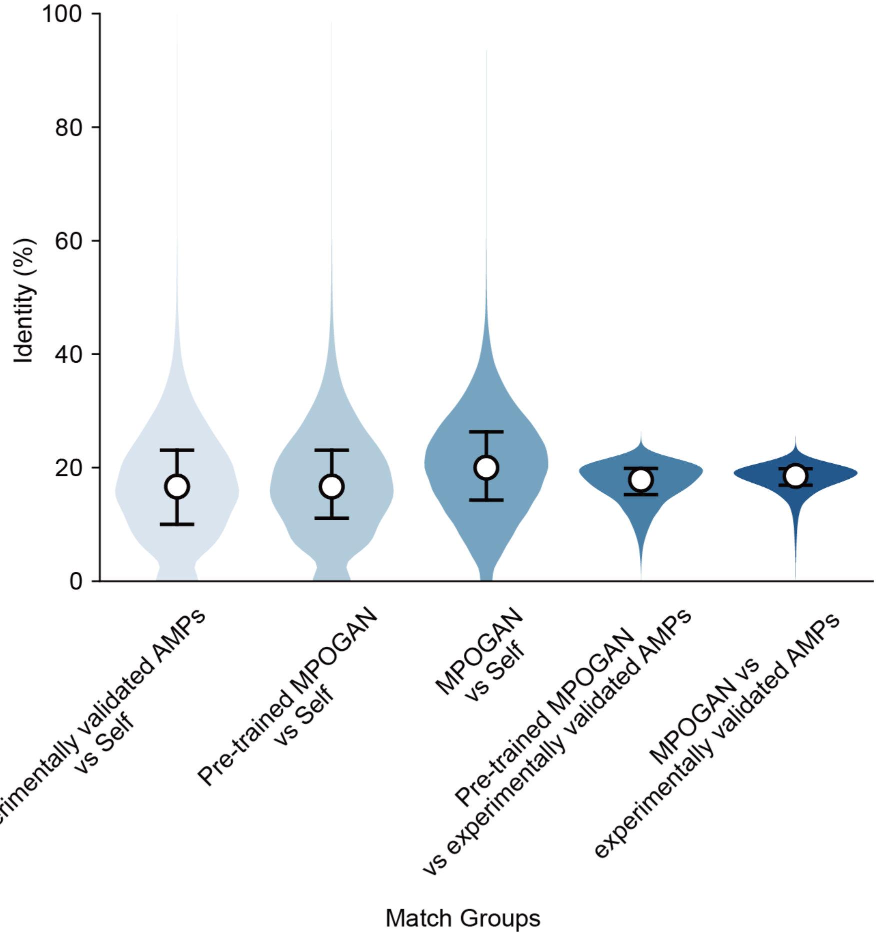

Supplementary Figure 15 Violin plots showing distributions of match scores obtained from comparisons between different groups of peptides. Identity is obtained through pairwise sequence alignment of amino acid sequences. The white dots indicate the median of each distribution, and the black vertical lines denote the interquartile range of each distribution.

16 Preliminary selection pipeline before wet-experiment validation

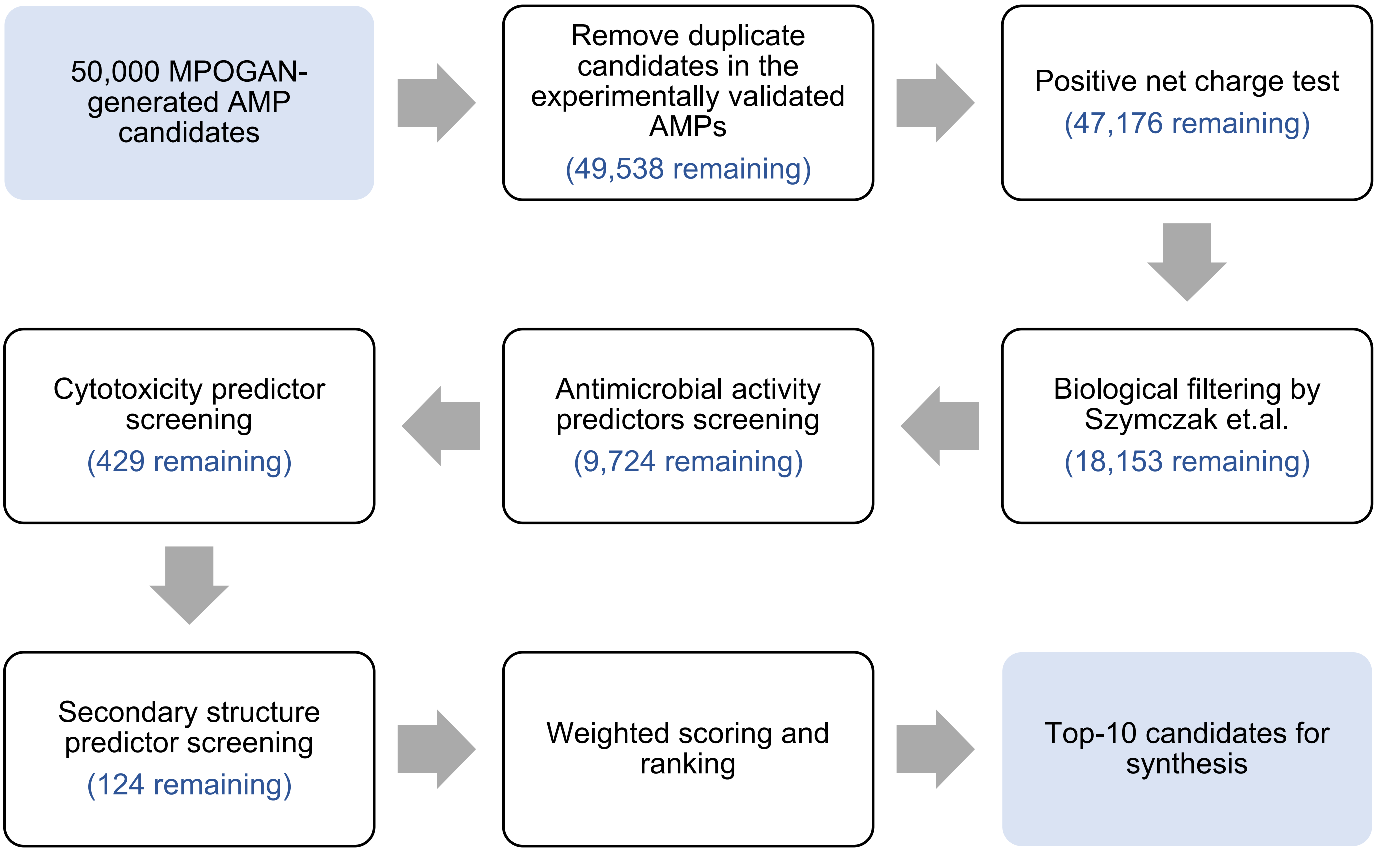

Supplementary Figure 16 Preliminary selection pipeline before wet-experiment validation.

17 Supplementary ablation studies of model-embedded evaluators for various combinations

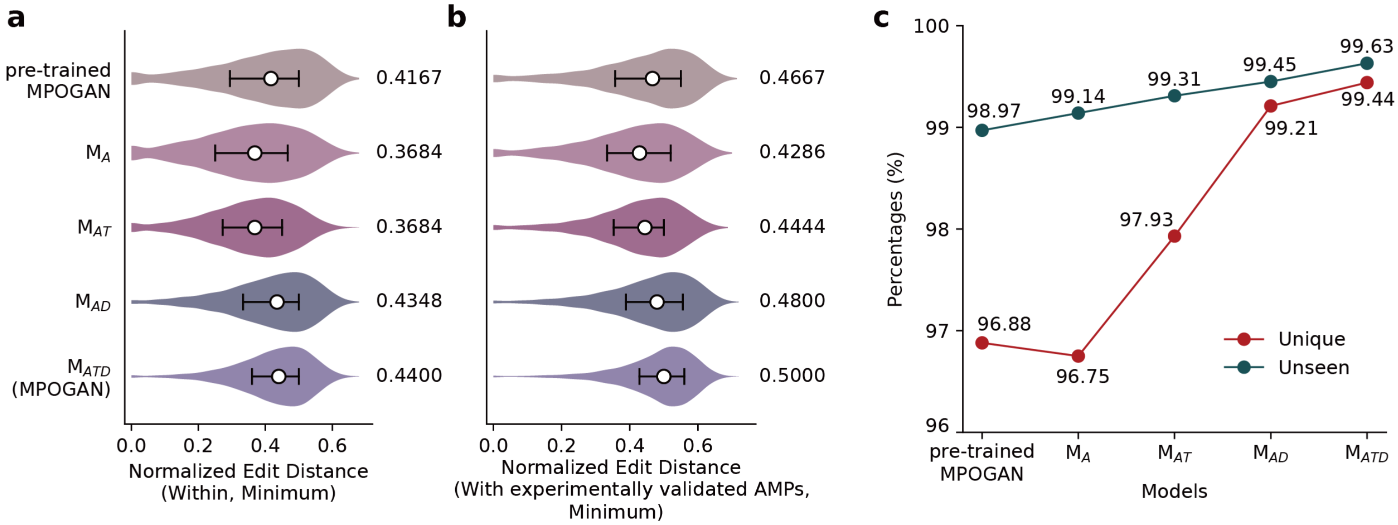

Supplementary Figure 17 Supplementary ablation studies of model-embedded evaluators for various combinations. **a** Minimum normalized within-group edit distance for AMP candidates generated by various combinatorial models. **b** Minimum normalized inter-group edit distance for AMP candidates generated by various combinatorial models with respect to experimentally validated AMPs. **c** Percentage variation in the size of unique and unseen AMP candidate sets relative to the size of the AMP candidate set generated by the generator.

18 Supplementary ablation studies for the RTKU strategy

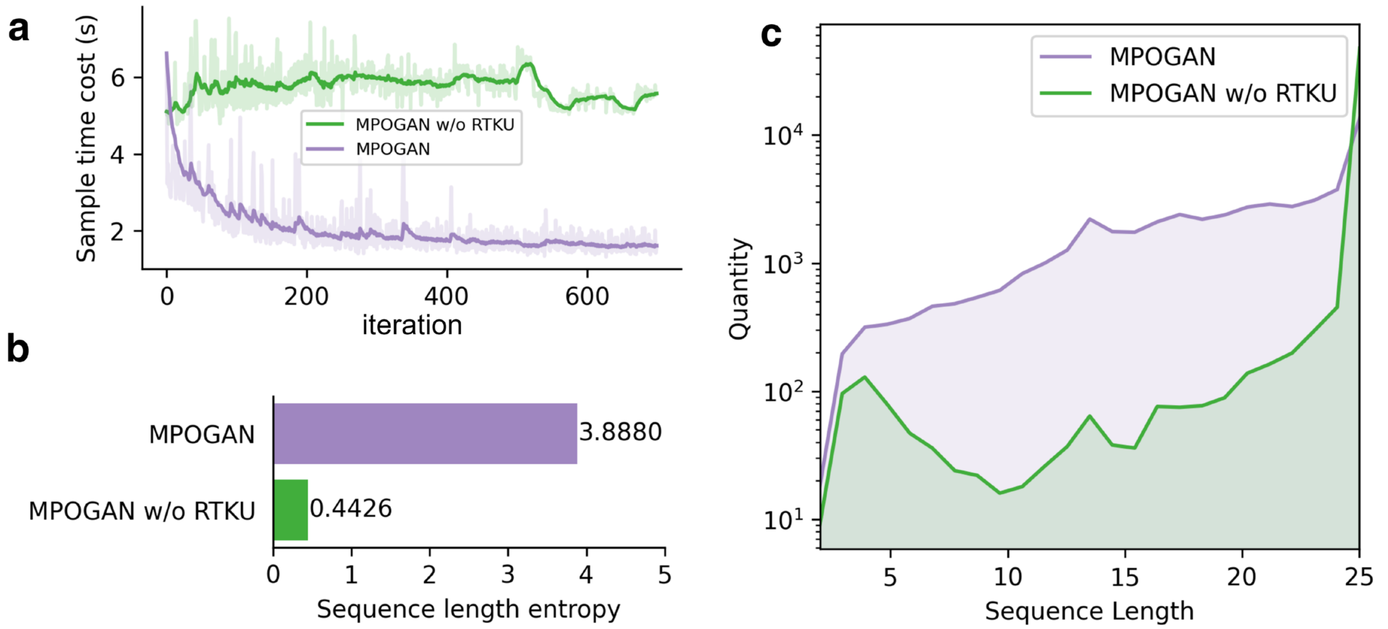

Supplementary Figure 18 Supplementary ablation studies for the RTKU strategy. **a** Comparison of the time required for sampling sequences from the generator during the MPO stage. The x-axis represents the number of iterations, and the y-axis represents the sampling time. MPOGAN w/o RTKU refers to the variant of MPOGAN that does not use the RTKU strategy but instead updates the dynamic dataset in a first-in-first-out manner with sequences output by the model-embedded screening pipeline (trained with the same hyperparameters as MPOGAN). **b-c** Comparison of sequence length entropy (**b**) and distributions (**c**) between sequences generated by MPOGAN and those generated by MPOGAN w/o RTKU.

19 Alpha-helical wheels of 12 synthesized peptides

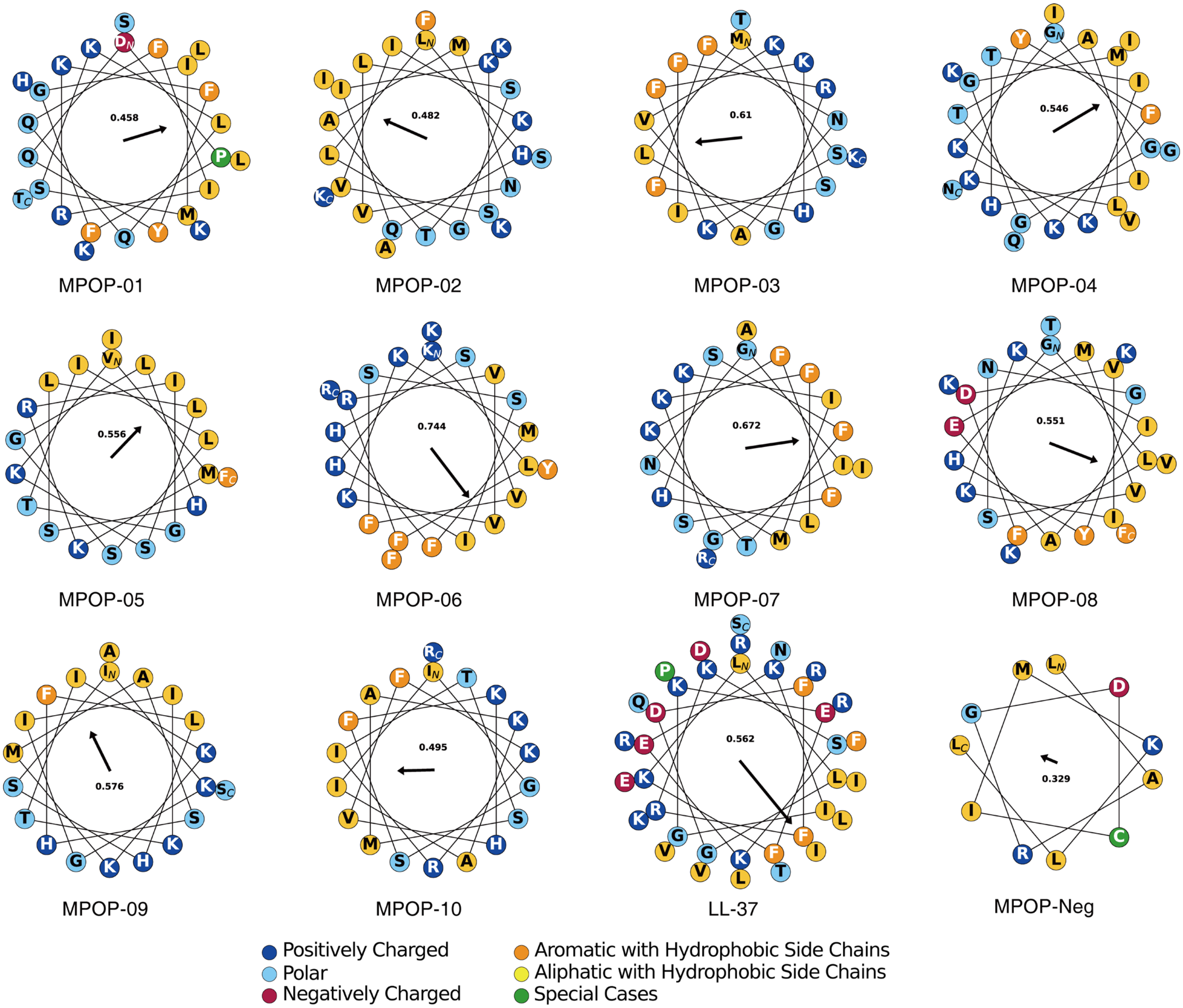

Supplementary Figure 19 Alpha-helical wheels of 12 synthesized peptides. The numbers in the wheels represent Eisenberg hydrophobic moment.

20 3D structures of synthesized peptides predicted using AlphaFold3

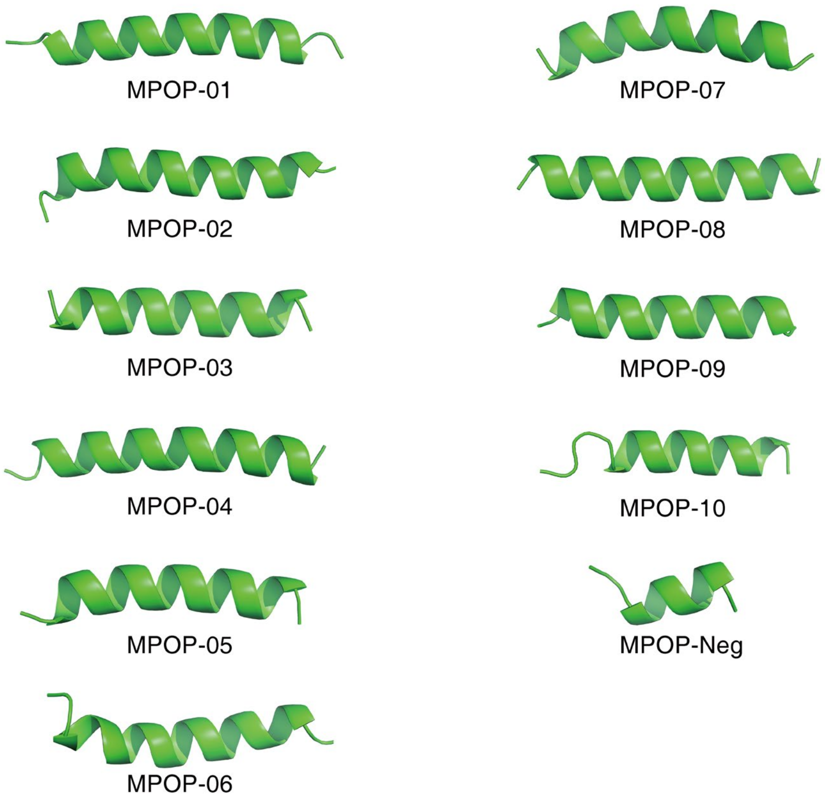

Supplementary Figure 20 3D structures of synthesized peptides predicted using AlphaFold3.

21 Density changes along the Z-axis during peptide-membrane molecular dynamics simulations of MPOP-03 and MPOP-07

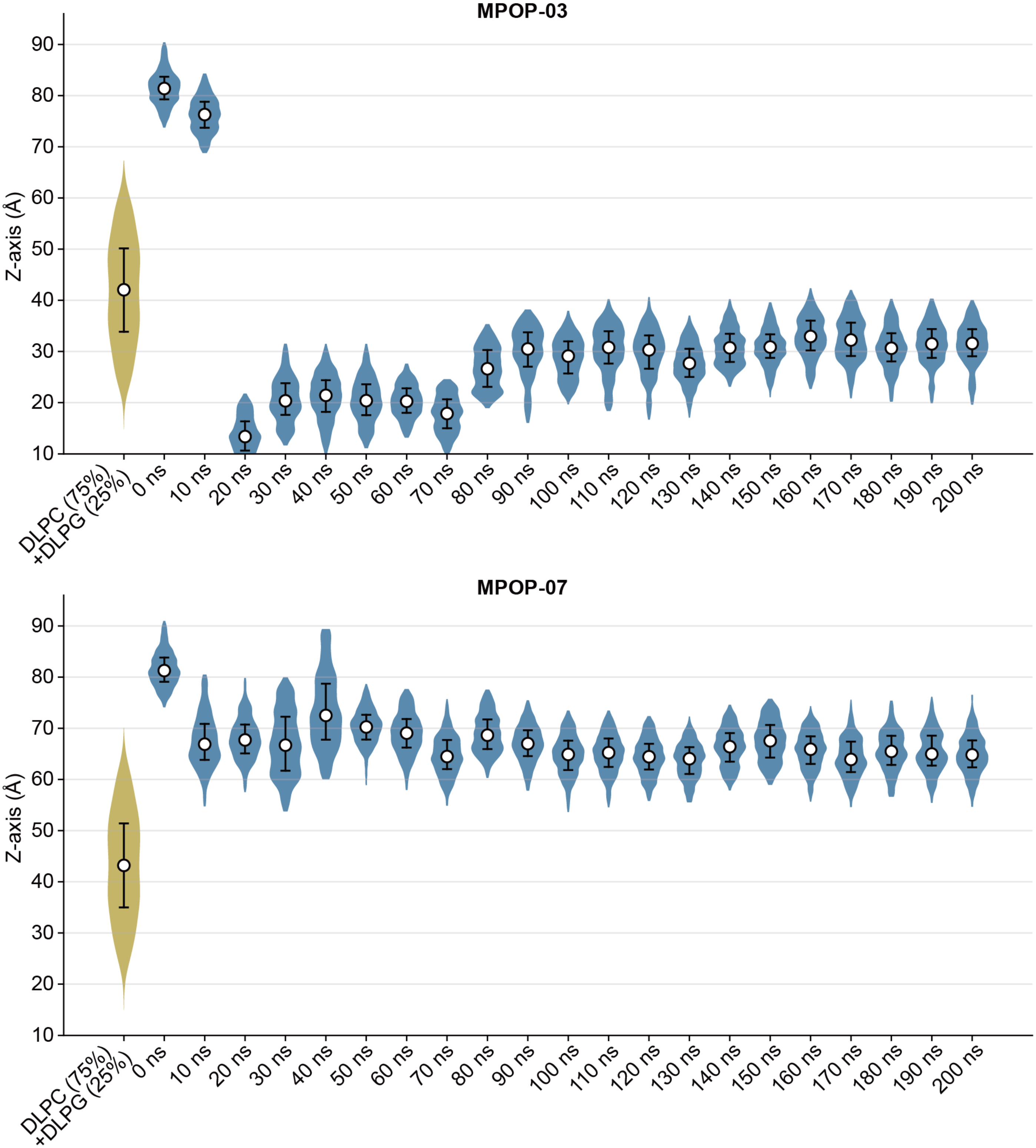

Supplementary Figure 21 Density changes along the Z-axis during peptide-membrane molecular dynamics simulations of MPOP-03 and MPOP-07.

22 Cell viability of MPOPs on MC3T3-E1 at various concentrations

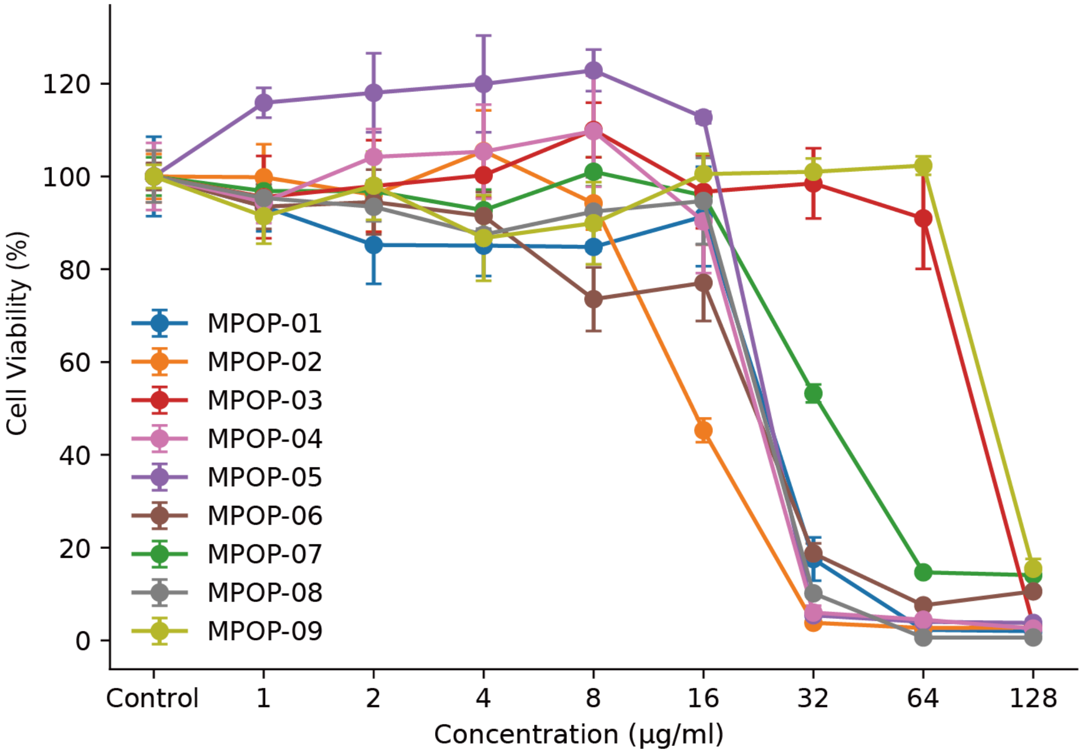

Supplementary Figure 22 Cell viability (%) of MPOPs on MC3T3-E1 cells at various concentrations (μg/mL) (n=3 independent experiments, mean $\pm$s.d.). The data were plotted as AMP concentration versus the percentage of living cells.

23 MIC values of 12 synthesized peptides

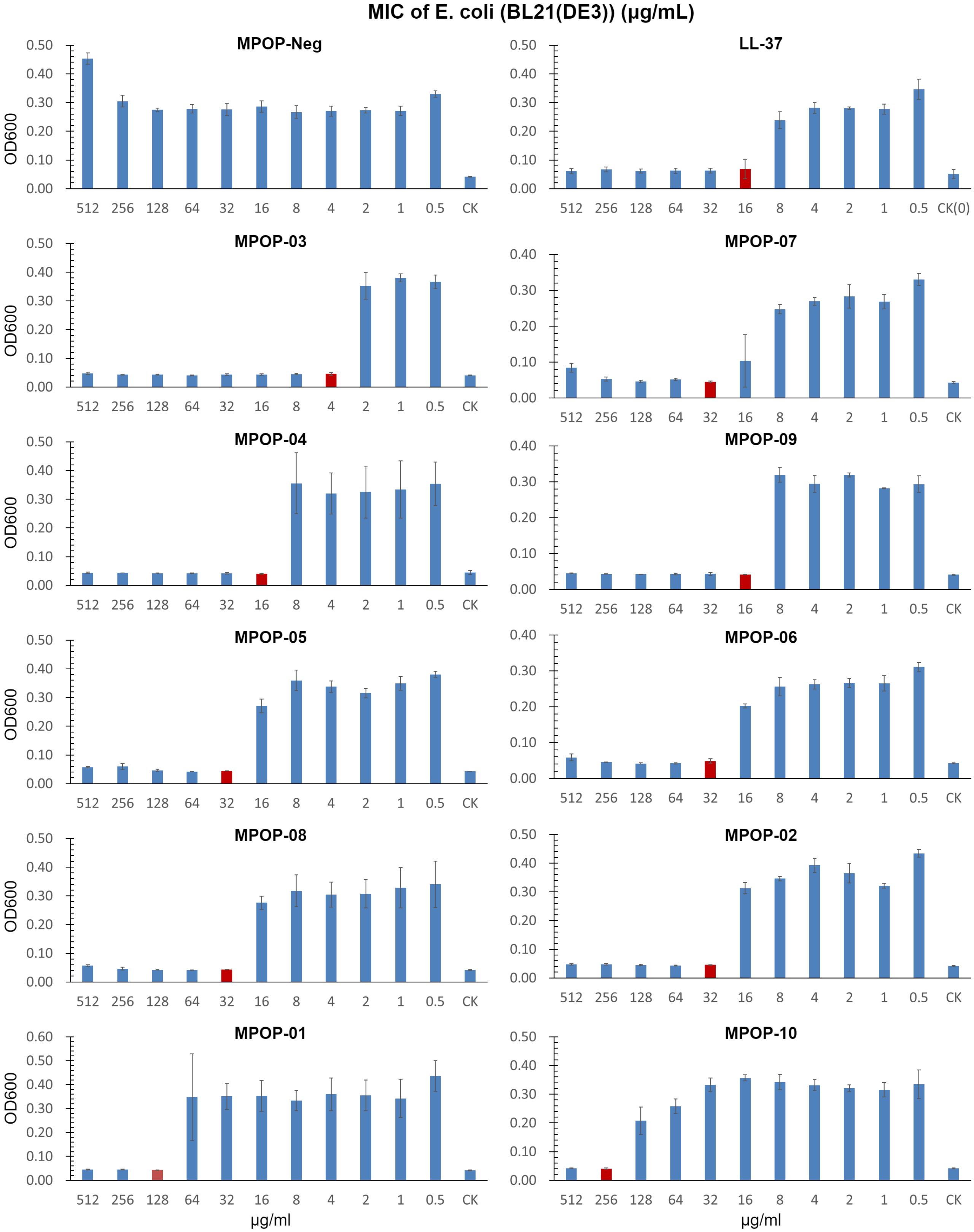

Supplementary Figure 23 MIC values of synthesized peptides against *E. coli*.

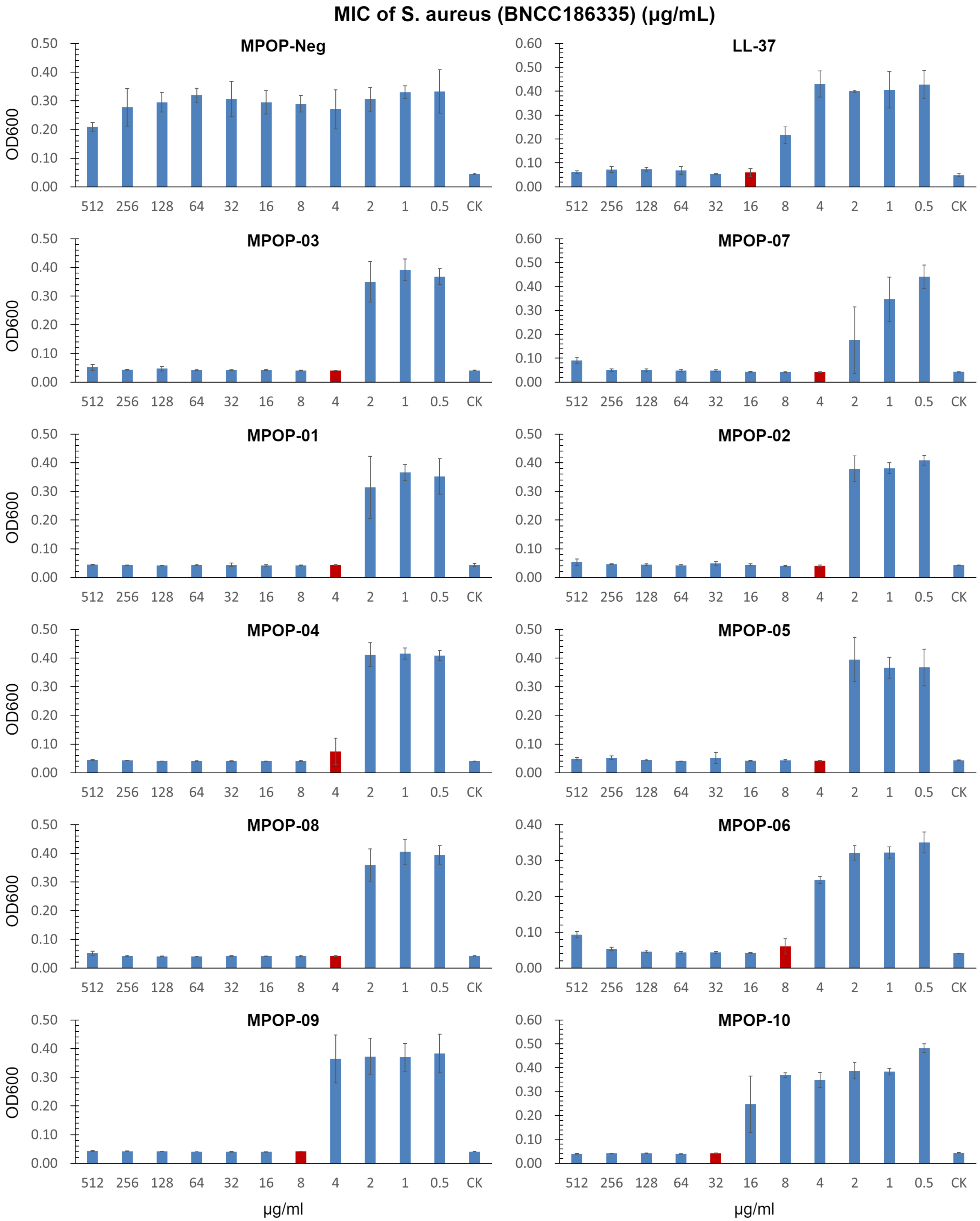

Supplementary Figure 24 MIC values of synthesized peptides against *S. aureus*.

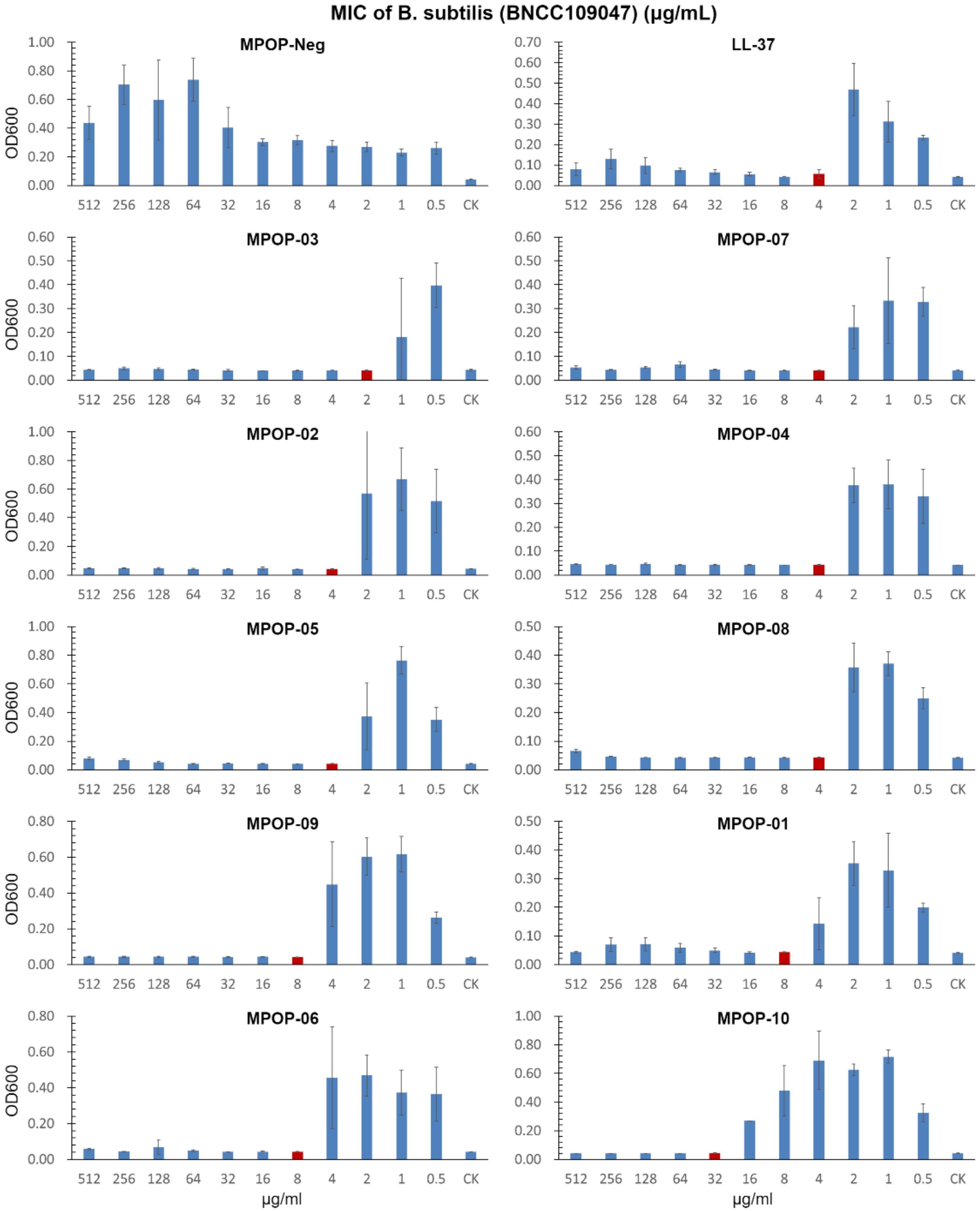

Supplementary Figure 25 MIC values of synthesized peptides against *B. subtilis*.

References

1. N. Sharma, L. D. Naorem, S. Jain, G. P. S. Raghava, ToxinPred2: an improved method for predicting toxicity of proteins. *Brief. Bioinform.* **23**, bbac174 (2022).

2. S. Gupta, P. Kapoor, K. Chaudhary, A. Gautam, R. Kumar, O. S. D. D. Consortium, G. P. S. Raghava, In Silico Approach for Predicting Toxicity of Peptides and Proteins. *PLOS ONE* **8**, e73957 (2013).

3. L. Fu, B. Niu, Z. Zhu, S. Wu, W. Li, CD-HIT: accelerated for clustering the next-generation sequencing data. *Bioinformatics* **28**, 3150–3152 (2012).

4. Y. Liu, X. Zhang, Y. Liu, Y. Su, X. Zeng, G. G. Yen, Evolutionary multi-objective optimization in searching for various antimicrobial peptides [feature]. *IEEE Comput. Intell. Mag.* **18**, 31–45 (2023).

5. S. N. Dean, S. A. Walper, Variational Autoencoder for Generation of Antimicrobial Peptides. *ACS Omega* **5**, 20746–20754 (2020).

6. D. Nagarajan, T. Nagarajan, N. Roy, O. Kulkarni, S. Ravichandran, M. Mishra, D. Chakravortty, N. Chandra, Computational antimicrobial peptide design and evaluation against multidrug-resistant clinical isolates of bacteria. *J. Biol. Chem.* **293**, 3492–3509 (2018).

7. P. Das, K. Wadhawan, O. Chang, T. Sercu, C. D. Santos, M. Riemer, V. Chenthamarakshan, I. Padhi, A. Mojsilovic, PepCVAE: Semi-Supervised Targeted Design of Antimicrobial Peptide Sequences. arXiv arXiv:1810.07743 [Preprint] (2018). https://doi.org/10.48550/arXiv.1810.07743.

8. P. Szymczak, M. Możejko, T. Grzegorzek, R. Jurczak, M. Bauer, D. Neubauer, K. Sikora, M. Michalski, J. Sroka, P. Setny, W. Kamysz, E. Szczurek. *Nat. Commun.* **14**, 1453 (2023).

9. A. T. Müller, J. A. Hiss, G. Schneider, Recurrent Neural Network Model for Constructive Peptide Design. *J. Chem. Inf. Model.* **58**, 472–479 (2018).

10. A. Tucs, D. P. Tran, A. Yumoto, Y. Ito, T. Uzawa, K. Tsuda, Generating Ampicillin-Level Antimicrobial Peptides with Activity-Aware Generative Adversarial Networks. *ACS Omega* **5**, 22847–22851 (2020).

11. T.-T. Lin, L.-Y. Yang, C.-T. Wang, G.-W. Lai, C.-F. Ko, Y.-H. Shih, S.-H. Chen, C.-Y. Lin, Discovering novel antimicrobial peptides in generative adversarial network. *BioRxiv*, 2021–11 (2021).

12. A. Gupta, J. Zou, Feedback Gan for Dna Optimizes Protein Functions. *Nat. Mach. Intell.* **1**, 105–111 (2019).

13. J. Wang, J. Feng, Y. Kang, P. Pan, J. Ge, Y. Wang, M. Wang, Z. Wu, X. Zhang, J. Yu, others, Discovery of antimicrobial peptides with notable antibacterial potency by an LLM-based foundation model. *Sci. Adv.* **11**, eads8932 (2025).

14. Y. Wang, X. Liu, F. Huang, Z. Xiong, W. Zhang, "A multi-modal contrastive diffusion model for therapeutic peptide generation" in *Proceedings of the AAAI Conference on Artificial Intelligence* (2024)vol. 38, pp. 3–11.

15. J. Luo, X. Liu, J. Li, Q. Chen, J. Chen, Flexible and Controllable Protein Design by Prefix-tuning Large-Scale Protein Language Models. *bioRxiv*, 2023–12 (2023).

16. C. Li, D. Sutherland, S. A. Hammond, C. Yang, F. Taho, L. Bergman, S. Houston, R. L. Warren, T. Wong, L. M. N. Hoang, C. E. Cameron, C. C. Helbing, I. Birol, AMPlify: attentive deep learning model

for discovery of novel antimicrobial peptides effective against WHO priority pathogens. *BMC Genomics* **23**, 77 (2022).

17. D. Veltri, U. Kamath, A. Shehu, Deep learning improves antimicrobial peptide recognition. *Bioinformatics* **34**, 2740–2747 (2018).

18. C. Keylock, Simpson diversity and the Shannon–Wiener index as special cases of a generalized entropy. *Oikos* **109**, 203–207 (2005).

19. C. D. Santos-Junior, S. Pan, X.-M. Zhao, L. P. Coelho, MACREL: antimicrobial peptide screening in genomes and metagenomes. bioRxiv [Preprint] (2020). https://doi.org/10.1101/2019.12.17.880385.

20. T. J. Lawrence, D. L. Carper, M. K. Spangler, A. A. Carrell, T. A. Rush, S. J. Minter, D. J. Weston, J. L. Labbé, amPEPpy 1.0: a portable and accurate antimicrobial peptide prediction tool. *Bioinforma. Oxf. Engl.* **37**, 2058–2060 (2021).

21. Y. Ma, Z. Guo, B. Xia, Y. Zhang, X. Liu, Y. Yu, N. Tang, X. Tong, M. Wang, X. Ye, J. Feng, Y. Chen, J. Wang, Identification of antimicrobial peptides from the human gut microbiome using deep learning. *Nat. Biotechnol.* **40**, 921–931 (2022).

22. W. F. Porto, K. C. V. Ferreira, S. M. Ribeiro, O. L. Franco, Sense the Moment: a highly sensitive antimicrobial activity predictor based on hydrophobic moment. bioRxiv [Preprint] (2021). https://doi.org/10.1101/2020.07.15.205419.

23. D. Veltri, U. Kamath, A. Shehu, Deep learning improves antimicrobial peptide recognition. *Bioinformatics* **34**, 2740–2747 (2018).

24. H. Lee, S. Lee, I. Lee, H. Nam, AMP-BERT: Prediction of antimicrobial peptide function based on a BERT model. *Protein Sci.* **32**, e4529 (2023).

25. AmPEP: Sequence-based prediction of antimicrobial peptides using distribution patterns of amino acid properties and random forest | Scientific Reports. https://www.nature.com/articles/s41598-018-19752-w.

26. J. Abramson, J. Adler, J. Dunger, R. Evans, T. Green, A. Pritzel, O. Ronneberger, L. Willmore, A. J. Ballard, J. Bambrick, others, Accurate structure prediction of biomolecular interactions with AlphaFold 3. *Nature* **630**, 493–500 (2024).

27. M. J. Abraham, T. Murtola, R. Schulz, S. Páll, J. C. Smith, B. Hess, E. Lindahl, GROMACS: High performance molecular simulations through multi-level parallelism from laptops to supercomputers. *SoftwareX* **1**, 19–25 (2015).

28. B. Hess, H. Bekker, H. J. Berendsen, J. G. Fraaije, LINCS: a linear constraint solver for molecular simulations. *J. Comput. Chem.* **18**, 1463–1472 (1997).

29. U. Essmann, L. Perera, M. L. Berkowitz, T. Darden, H. Lee, L. G. Pedersen, A smooth particle mesh Ewald method. *J. Chem. Phys.* **103**, 8577–8593 (1995).

30. A. Pandi, D. Adam, A. Zare, V. T. Trinh, S. L. Schaefer, M. Burt, B. Klabunde, E. Bobkova, M. Kushwaha, Y. Foroughijabbari, P. Braun, C. Spahn, C. Preußer, E. Pogge von Strandmann, H. B. Bode, H. von Buttlar, W. Bertrams, A. L. Jung, F. Abendroth, B. Schmeck, G. Hummer, O. Vázquez, T. J. Erb, Cell-free biosynthesis combined with deep learning accelerates de novo-development of antimicrobial peptides. *Nat. Commun.* **14**, 7197 (2023).

31. H. J. Berendsen, J. van Postma, W. F. Van Gunsteren, A. DiNola, J. R. Haak, Molecular dynamics with coupling to an external bath. *J. Chem. Phys.* **81**, 3684–3690 (1984).

32. M. Parrinello, A. Rahman, Polymorphic transitions in single crystals: A new molecular dynamics method. *J. Appl. Phys.* **52**, 7182–7190 (1981).

33. G. Bussi, D. Donadio, M. Parrinello, Canonical sampling through velocity rescaling. *J. Chem. Phys.* **126** (2007).

34. W. L. DeLano, others, Pymol: An open-source molecular graphics tool. *CCP4 Newsl Protein Crystallogr* **40**, 82–92 (2002).

35. J. Huang, A. D. MacKerell Jr, CHARMM36 all-atom additive protein force field: Validation based on comparison to NMR data. *J. Comput. Chem.* **34**, 2135–2145 (2013).

36. S. Jo, T. Kim, V. G. Iyer, W. Im, CHARMM-GUI: a web-based graphical user interface for CHARMM. *J. Comput. Chem.* **29**, 1859–1865 (2008).

37. P. Das, T. Sercu, K. Wadhawan, I. Padhi, S. Gehrmann, F. Cipcigan, V. Chenthamarakshan, H. Strobelt, C. dos Santos, P.-Y. Chen, Y. Y. Yang, J. P. K. Tan, J. Hedrick, J. Crain, A. Mojsilovic, Accelerated antimicrobial discovery via deep generative models and molecular dynamics simulations. *Nat. Biomed. Eng.* **5**, 613–623 (2021).

38. A. S. Rathore, N. Kumar, S. Choudhury, N. K. Mehta, G. P. Raghava, Prediction of hemolytic peptides and their hemolytic concentration. *Commun. Biol.* **8**, 176 (2025).

39. J. Witten, Z. Witten, Deep learning regression model for antimicrobial peptide design. bioRxiv [Preprint] (2019). https://doi.org/10.1101/692681.
